# Mechanism Study of Xiaoyao San against Nonalcoholic Steatohepatitis-Related Liver Fibrosis Based on a Combined Strategy of Transcriptome Analysis and Network Pharmacology

**DOI:** 10.3390/ph17091128

**Published:** 2024-08-27

**Authors:** Di Yan, Xiaoling Zhang, Chengmei Ma, Wenting Huang, Mimi Hao, Lan Xie

**Affiliations:** 1 School of Basic Medical Sciences, Chengdu University of Traditional Chinese Medicine, Chengdu 611137, China; yandi1@stu.cdutcm.edu.cn; 2 Medical Systems Biology Research Center, School of Medicine, Tsinghua University, Beijing 100084, China; hwt17@mails.tsinghua.edu.cn; 3 National Engineering Research Center for Beijing Biochip Technology, Beijing 102206, China; xiaolingzhang@capitalbio.com (X.Z.); chengmeima@capitalbio.com (C.M.); mimihao@capitalbio.com (M.H.)

**Keywords:** Xiaoyao San, NASH, liver fibrosis, transcriptome analysis, network pharmacology

## Abstract

Nonalcoholic fatty liver disease (NAFLD) is the leading cause of liver disease worldwide. Nonalcoholic steatohepatitis (NASH) is an advanced form of NAFLD. The livers of patients with NASH are more likely to develop fibrosis. Xiaoyao San (XYS) is a classic traditional Chinese medicine (TCM) formula that has been widely used in treating liver diseases. In this study, we elucidated the effects and mechanism of XYS in treating NASH-related liver fibrosis by combining high-throughput sequencing-based high-throughput screening with network pharmacology analysis. Our work revealed that XYS may play a role in preventing NASH-related liver fibrosis by regulating biological functions related to the extracellular matrix (ECM), inflammation, and metabolism. Additionally, *Bupleuri Radix*, *Poria*, *Zingiberis Rhizoma Recens*, and *Paeoniae Radix Alba* are the key herbs of XYS that could partially represent the functions of XYS. These regulatory effects are mediated by targeting signal transducer and activator of transcription 3 (STAT3), nuclear factor kappa B (NFκB), and peroxisome proliferator-activated receptor gamma (PPARγ) signaling. Narcissin, casuarictin, and γ-sitosterol were identified as representative active compounds in XYS targeting STAT3, NFκB, and PPARγ, respectively. Taken together, our findings provide a novel strategy for investigating the pharmacological effects and biological mechanisms of a TCM formula.

## 1. Introduction

Liver fibrosis is a reversible wound-healing process that results from chronic liver damage and commonly occurs in patients with chronic liver diseases of most etiologies, such as hepatitis B virus (HBV) and hepatitis C virus (HCV) infection, nonalcoholic fatty liver disease (NAFLD), and alcohol abuse [1]. Currently, NAFLD is the most common chronic liver disease, with a global prevalence of 32.4% and an incidence of 46.9 cases per 1000 person-years [2]. Nonalcoholic steatohepatitis (NASH) is an advanced form of NAFLD that is strongly associated with liver fibrosis, which can progress to more serious complications, such as cirrhosis and hepatocellular carcinoma. Surgical resection and liver translation are the only methods available for prolonging the survival of patients with hepatocellular carcinoma. Thus, early management of NASH and liver fibrosis is critical for reducing the morbidity and mortality of chronic liver diseases.

In the last decades, the treatment of NASH and liver fibrosis is generally etiological. Although many anti-NASH and antifibrotic medicines are under investigation, none have been licensed [3,4]. Not until recently did the FDA approve the first drug, resmetirom (Rezdiffra), for the management of NASH with fibrosis. Resmetirom provides an effective therapeutic strategy for NASH-related liver fibrosis; however, the side effects of drug-induced liver toxicity and gallbladder issues should not be ignored [5]. Traditional Chinese medicine (TCM) is a cultural treasure of China, and many TCM formulae have been widely used for treating NAFLD and liver fibrosis, with numerous clinical trials and studies showing their notable curative effects, such as Chaihu Shugan San, Fuzhenghuayu capsule, etc. [6,7].

Xiaoyao San (XYS) is a classic prescription that was first documented in *Prescriptions from the Taiping Benevolent Pharmaceutical Bureau* (Song Dynasty, 960–1127 A.D.). XYS is composed of eight herbs, namely, *Bupleuri Radix* (BR, Chai Hu), *Angelicae Sinensis Radix* (ASR, Dang Gui), *Paeoniae Radix Alba* (PRA, Bai Shao), *Atractylodis Macrocephalae Rhizoma* (AMR, Bai Zhu), *Poria* (Fu Ling), *Zingiberis Rhizoma Recens* (ZRR, Sheng Jiang), *Menthae Haplocalycis Herba* (MHH, Bo He), and *Glycyrrhizae Radix Et Rhizoma Praeparata Cum Melle* (GRR, Zhi Gan Cao). In ancient times, XYS was used to treat liver depression syndrome, blood deficiency, and spleen qi deficiency syndrome. TCM theory believes that liver qi depression is the etiology of breast cancer and depressive disorder. Hence, XYS is widely used to treat breast cancer and depression. A meta-analysis involving 17 randomized controlled trial (RCT) studies, with 1207 patients, evaluated the efficacy of modified XYS combined with chemotherapy in treating breast cancer and concluded that the combination increased the efficacy and reduced the side effects caused by chemotherapy [8]. In addition, an RCT study of orally administered XYS in 41 patients with depression, showed that XYS effectively alleviated depressive disorder symptoms in mild and moderate depression patients [9].

In addition, XYS has been used for various chronic liver diseases through dispersing stagnated liver qi to relieve qi stagnation. The clinical efficacy study of XYS combined with interferon α in treating 193 chronic hepatitis B patients revealed that the combination improved the anti-viral effect and reduced the adverse reactions of interferon α [10]. Liu et al. reviewed 12 RCT studies with 1012 patients and assessed the efficacy of XYS for treating NAFLD, concluding that XYS can ameliorate NAFLD by regulating blood lipids and protecting liver function [11]. An animal study showed that XYS decreased hepatic steatosis and triglyceride levels in ovariectomized apolipoprotein E knockout (ApoE−/−) mice [12]. In addition, another animal study suggested that XYS significantly alleviated the histopathological fibrotic changes and improved the liver function of carbon tetrachloride-induced liver fibrosis rats [13]. These studies provide rich clinical and preclinical evidence for XYS in treating NASH-related liver fibrosis.

However, TCM formulae are composed of multiple herbs that contain multiple active ingredients that usually regulate multiple targets and pathways, and previous studies of XYS in treating NAFLD and liver fibrosis were mainly focused on the pharmacology of the XYS formula and lack of investigation of the pharmacology of XYS constituent herbs [12,13]. Thus, fully elucidating the molecular mechanism of XYS and better understanding the role of constituent herbs in XYS is necessary.

**Figure 1:**
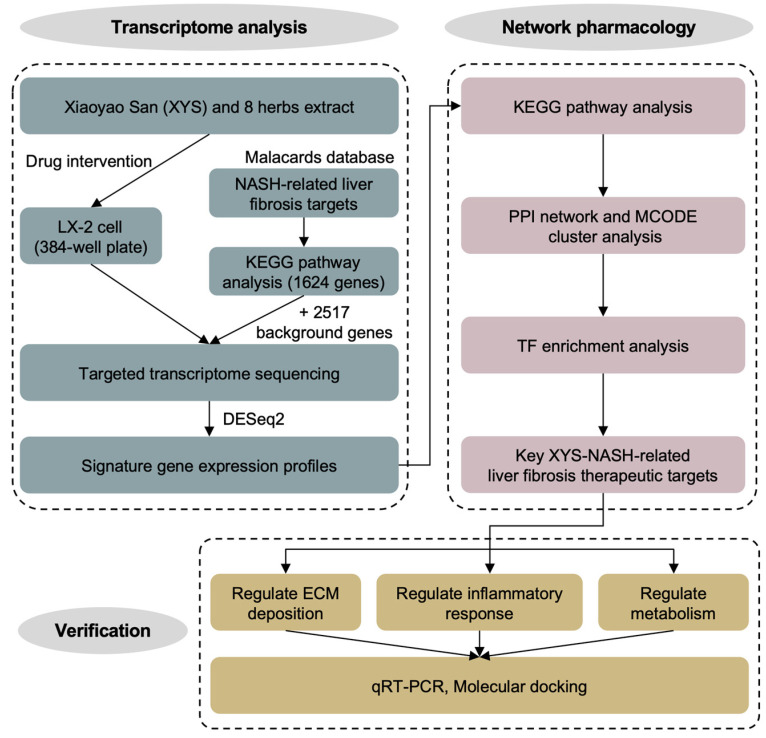
Research technical roadmap.

Hepatocyte lipotoxicity, inflammation, and hepatic stellate cell (HSC) activation are the key pathological mechanisms in NASH-related liver fibrosis [14]. It is believed that the activation of HSC is responsible for the fibrotic histological changes in NASH liver, especially the deposition of the type I collagen-rich extracellular matrix (ECM) [15]. Therefore, the human LX-2 (Lieming Xu-2) hepatic stellate cell line was used in this work. Firstly, we established targeted transcriptome profiles of LX-2 cells treated with XYS and its constituent herbs via high-throughput sequencing-based high-throughput screening (HTS^2^). HTS^2^ is a high-throughput targeted transcriptome analysis platform based on RNA-mediated oligonucleotide annealing, selection, and ligation with next-generation sequencing (RASL-seq). It can be used to detect changes in gene expression in response to thousands of drug treatments in parallel. Integrated transcriptome profiling and network pharmacology analysis revealed that XYS has strong potential for treating NASH-related liver fibrosis through the regulation of signal transducer and activator of transcription 3 (STAT3), nuclear factor kappa B (NFκB), and peroxisome proliferator-activated receptor gamma (PPARγ) signaling and subsequently exerts antifibrotic, anti-inflammatory and metabolic regulatory effects. Additionally, molecular docking analysis revealed that narcissin, casuarictin, and γ-sitosterol are the representative active compounds in XYS and target STAT3, NFκB, and PPARγ, respectively (Research flowchart, Figure 1).

## 2. Results

### 2.1. Construction of Signature Gene Expression Profiles for the Response to Treatment with XYS and Its Constituent Herbs

**Figure 2:**
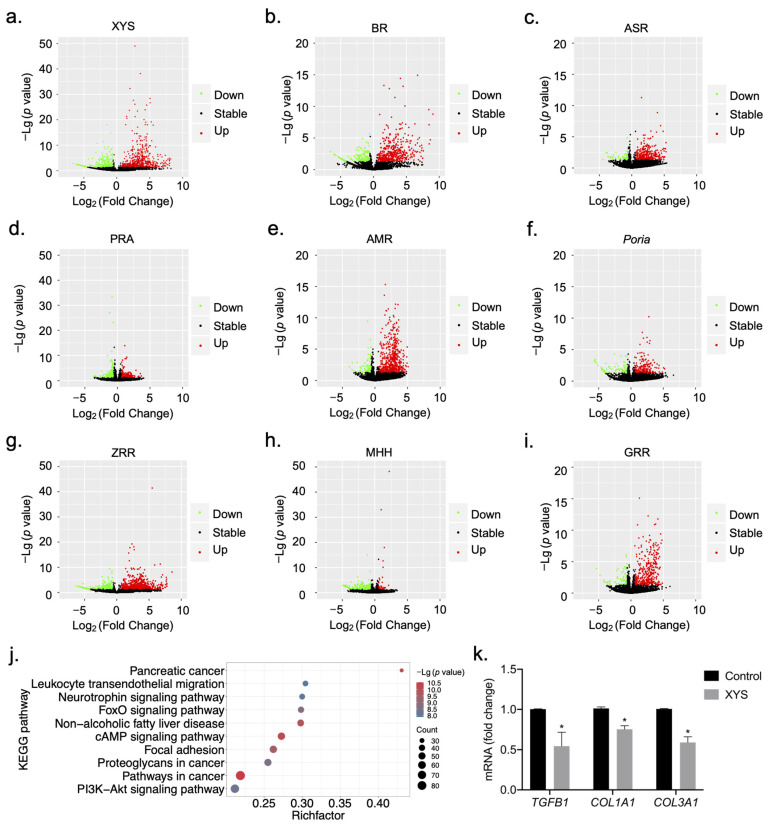
Volcano plot of DEGs and biological functional analysis of XYS: (**a**–**i**) Volcano plot of the DEGs of XYS and its 8 constituent herbs. Red plot, upregulated gene; green plot, downregulated gene; black plot, stable gene. (**j**) Top 10 significantly enriched KEGG pathways in the 916 DEGs in response to XYS treatment. (**k**) *TGFB1*, *COL1A1*, and *COL3A1* mRNA expression in LX-2 cells treated with 400 μg/mL XYS for 24 h. The data are shown as the mean ± SD. Statistical analyses were conducted using paired Student’s *t* tests. * *p* < 0.05 vs. the control group.

To construct signature gene expression profiles for the response to XYS and its eight constituent herbs, the following steps were performed. First, extracts of XYS and the eight constituent herbs were prepared with 90% (*v*/*v*) ethanol. LX-2 cells were treated with extracts of XYS and the eight constituent herbs at the concentration of 100 μg/mL (cell viability was greater than 80%, Figure S1) for 24 h. By searching for NAFLD, NASH, and liver fibrosis-related genes in the human disease database Malacards [16], we identified 188 disease-related target genes. KEGG pathway enrichment was subsequently conducted, and 17 KEGG pathways were enriched in the 188 genes related to NAFLD, NASH, and liver fibrosis. A total of 1624 genes from these pathways were designed as probes. In addition, 1565 genes related to immunity, metabolism, digestion, cellular progress, and endocrine processes and 952 housekeeping genes were used as background genes; these genes were also used as probes for further research. Eventually, a set of 4141 genes was identified by HTS^2^. After data quality control and differential gene expression analysis, signature gene expression profiles related to the response to stimulation with XYS and its eight constituent herbs were established (Figure 2a–i, Tables S2 and S3). The numbers of genes differentially expressed in LX-2 cells in response to stimulation with XYS and the eight herbs are shown in Table 1. A total of 916 genes were differentially expressed in response to XYS stimulation, including 574 upregulated genes and 342 downregulated genes. Among the eight herbs, ZRR, AMR, and BR were associated with the largest numbers of DEGs.

### 2.2. Biological Functional Analysis of XYS and Its Constituent Herbs

As shown in Figure 2a, 574 genes were upregulated and 342 genes were downregulated in response to XYS stimulation.

NASH-related liver fibrosis involves serious pathological changes, including architectural changes (deposition of type I collagen-rich ECM), increased lipid accumulation, and immune cell infiltration [15]. To investigate the biological functions of the genes differentially regulated by XYS, KEGG pathway enrichment analysis was conducted. KEGG pathway enrichment analysis revealed that the top 10 pathways associated with the DEGs in the XYS treatment group were involved mainly in ECM-, inflammation-, and metabolism-related pathways, such as the Focal adhesion, Leukocyte transendothelial migration, NAFLD, and FoxO signaling pathways (Figure 2j). These results indicated that XYS may regulate the characteristic pathological processes involved in liver fibrosis, such as ECM deposition, metabolic dysregulation, and chronic inflammation. Next, we evaluated the antifibrotic effect of XYS on liver fibrosis through its regulation of several typical profibrotic genes. We treated LX-2 cells with XYS and evaluated the mRNA expression of *TGFB1*, *COL1A1*, and *COL3A1*, which play essential roles in the activation of HSCs and deposition of the ECM. qRT-PCR revealed that the mRNA expression of *TGFB1*, *COL1A1*, and *COL3A1* was significantly downregulated by XYS (Figure 2k).

**Figure 3:**
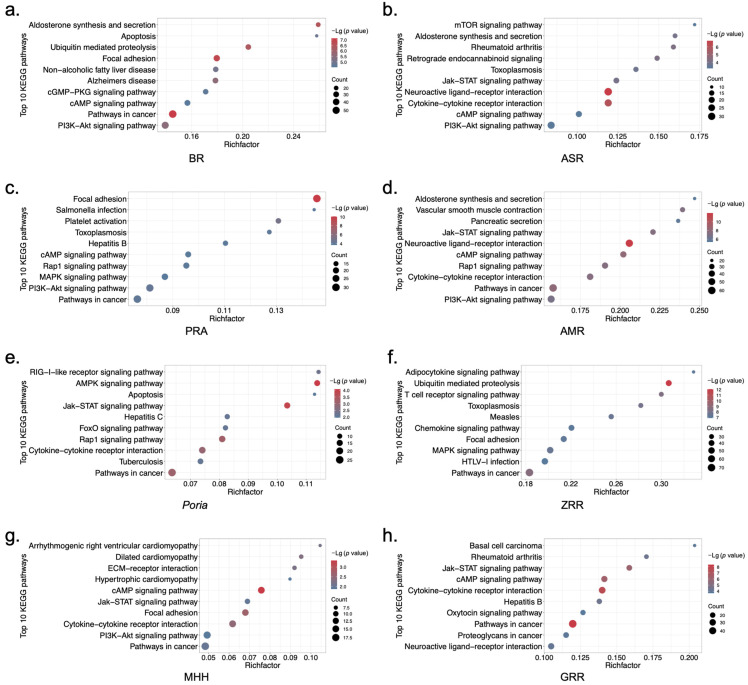
Biological functional analysis of the 8 constituent herbs of XYS: (**a**–**h**) Top 10 significantly enriched KEGG pathways in DEGs in response to treatment with BR, ASR, PRA, AMR, *Poria*, ZRR, MHH, or GRR.

**Figure 4:**
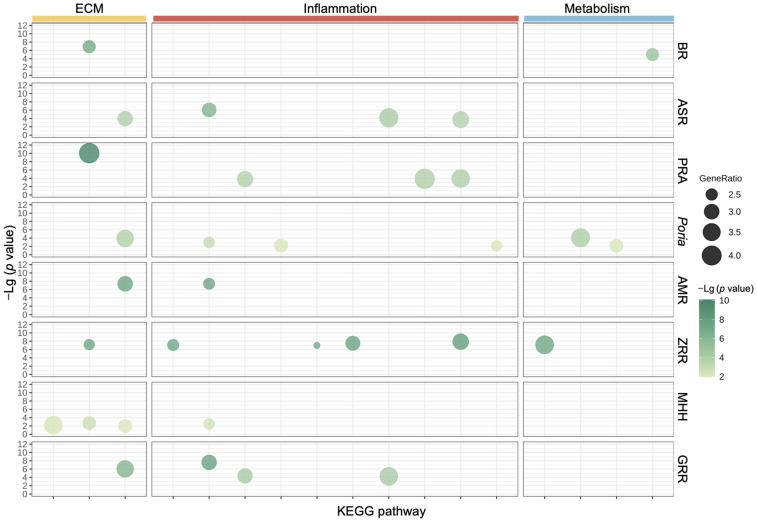
Bubble graph for the top 10 significantly enriched KEGG pathways of 8 herbs related to the ECM, inflammation, or metabolism. Each bubble represents one KEGG pathway. The bubble size correlates with the relative ratio of DEGs affecting each pathway among the total DEGs. Larger bubbles and greater Lg (*p* value) indicate greater significance.

To further explore the biological function relationships of the eight herbs in XYS, KEGG pathway enrichment analysis was subsequently conducted for each constituent herb. The analysis of the top 10 enriched KEGG pathways associated with all eight herbs revealed that the genes differentially regulated by all eight herbs were involved in biological functions related to the ECM, with enrichment of pathways such as the Focal adhesion, ECM–receptor interaction, and JAK-STAT signaling pathways. PRA, ASR, *Poria*, ZRR, and GRR are involved in mediating biological functions related to inflammation, with enrichment of various infectious pathways, such as the hepatitis B, hepatitis C, toxoplasmosis, measles, tuberculosis, and Salmonella infection pathways. The genes differentially regulated by BR, *Poria*, and ZRR were involved in the biological function of metabolism, with the enrichment of pathways such as the NAFLD, adipocytokine, FoxO, and AMPK signaling pathways (Figure 3). The abovementioned conclusion is visualized in Figure 4, and we hypothesized that the different constituent herbs in XYS contribute coordinately to regulating biological functions related to the ECM, inflammation, and metabolism.

### 2.3. Identification of Hub Genes and Clusters of XYS via PPI Network Analysis

As mentioned above, after analysis of the biological functions of the genes differentially regulated by XYS, we concluded that XYS has strong potential for treating NASH-related liver fibrosis by regulating processes related to the ECM, inflammation, and metabolism. We subsequently used STRING, a database of known and predicted PPIs, to further investigate the hub genes and gene clusters as targets of XYS.

**Figure 5:**
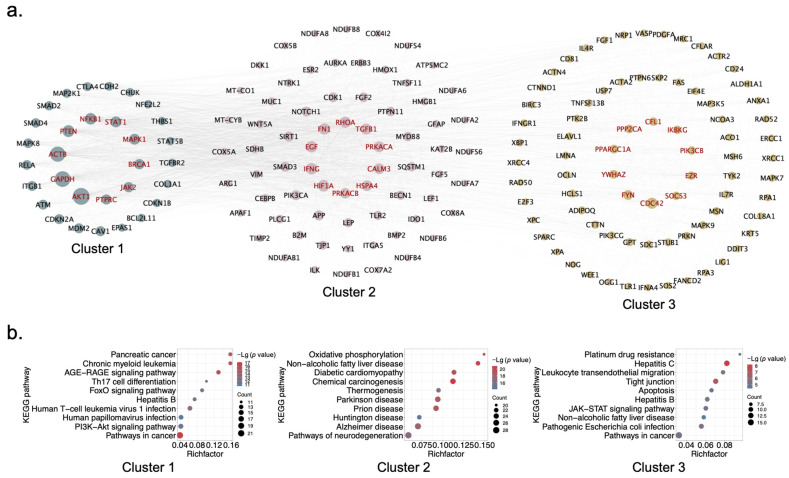
MCODE cluster analysis of the PPI network based on the DEGs of XYS: (**a**) The top 3 highest-scoring clusters of the PPI network. (**b**) The top 10 significantly enriched KEGG pathways of the 3 clusters.

A total of 916 DEGs in the XYS treatment group were mapped to the STRING database, and by setting the confidence score > 0.4, a PPI network that contained 903 interactive targets was ultimately constructed (Figure 5a). To further identify the hub targets in this network, cluster analysis was conducted with MCODE, a graph-theoretic clustering algorithm that can detect dense regions in a PPI network [17]. A total of 29 clusters were generated in the network, and the 3 highest-scoring clusters are shown in Figure 5a. Then, topological analysis was conducted by using the Network Analyzer plug-in in Cytoscape, and the size of each node was related to its degree centrality in the PPI network. The top 10 nodes in each cluster according to degree centrality are represented in red font and were considered the hub genes in each cluster.

As shown in Figure 5a, the identified hub genes were typical genes that participate in biological functions related to the ECM, inflammation, and metabolism and included nuclear factor kappa B subunit 1 (NFKB1), signal transducer and activator of transcription 1 (STAT1), transforming growth factor beta 1 (TGFB1), SMAD family member 3 (SMAD3), and peroxisome proliferator-activated receptor gamma coactivator 1 alpha (PPARGC1A).

KEGG pathway enrichment analysis was subsequently conducted to further understand the biological functions of the three clusters. The results showed that all three clusters were involved in metabolism-related pathways, such as the FoxO signaling pathway and NAFLD. Cluster 1 and cluster 3 were also involved in inflammation-related pathways, such as the hepatitis B, hepatitis C, and human papillomavirus infection pathways. Cluster 3 included ECM-related pathways, such as the tight junction and JAK-STAT signaling pathways (Figure 5b). These results support the idea that XYS may regulate processes related to the ECM, inflammation, and metabolism, which are characteristic of liver fibrosis and NASH pathogenesis.

### 2.4. Identification of Key TFs Regulated by XYS

**Figure 6:**
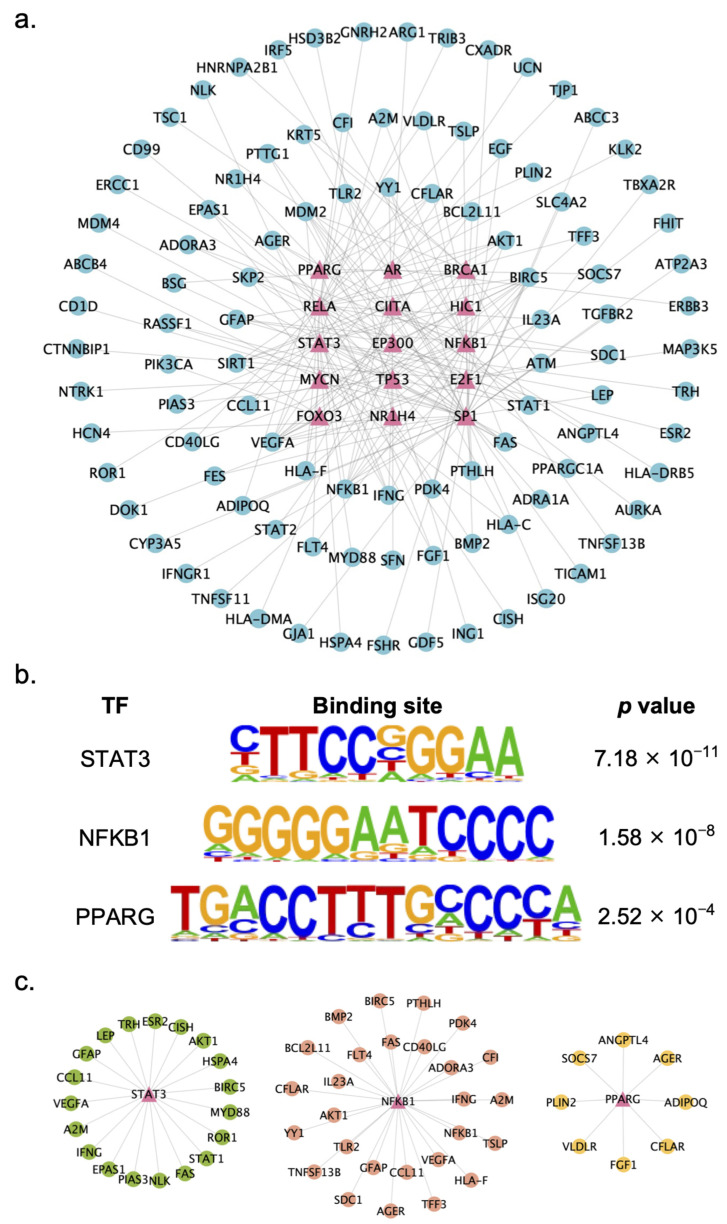
Identification of key transcription factors of XYS: (**a**) Gene-transcription factor regulatory interaction network of XYS. The pink triangle nodes represent transcription factors, and the blue round nodes represent downstream genes. (**b**) The motif binding sequences of STAT3, NFKB1, and PPARG. (**c**) Gene-transcription factor regulatory interaction network of STAT3, NFKB1, and PPARG and their downstream DEGs.

TFs play vital roles in the process of transcriptional regulation. Thus, to further explore the potential of XYS in treating liver fibrosis, TF enrichment analysis was conducted. By setting the criterion of |log_2_ fold change| ≥ 3, a total of 483 DEGs with greater expression fold changes among the 916 DEGs in the XYS group were mapped to the TRRUST database for TF enrichment. Significant enrichment of 72 TFs among the 483 DEGs was subsequently found. Finally, a gene–TF regulatory interaction network that included the 15 most significantly enriched TFs and their downstream DEGs was constructed and visualized with Cytoscape (Figure 6a).

Among the top 15 TFs, three TFs were closely related to the pathology of NASH and liver fibrosis, i.e., STAT3, NFκB, and PPARγ. STAT3 is a JAK signal transducer and is closely related to the occurrence and development of liver fibrosis [18]. Several studies have indicated that the use of STAT3 inhibitors in liver injury models has promising antifibrotic effects [19]. NFκB is a critical transcriptional regulator of the inflammatory response and plays a crucial role in mediating inflammatory responses in the liver [20]. PPARγ is a TF that has been implicated in the pathology of obesity, diabetes, and NAFLD [21]. Since STAT3, NFκB, and PPARγ are closely related to the pathology of NASH and liver fibrosis, we hypothesized that XYS may exert antifibrotic effects through these three TFs. The motif binding sites of each TF are shown in Figure 6b.

Then, we constructed gene–TF regulatory interaction networks of these three TFs and their downstream DEGs. As shown in Figure 6c, each network included the TF and its regulated DEGs. Among these three subnetworks, we identified several genes strongly associated with the pathology of NASH-related liver fibrosis. STAT1 and AKT serine/threonine kinase 1 (AKT1), which are regulated by STAT3, are involved in biological functional changes associated with the ECM and fibrosis. Activated HSCs are the major source of the ECM, and the present study indicated that the inhibition of STAT1 reverses the activation of HSCs [22]. A study demonstrated that the activation of AKT1 is associated with the remodeling processes of fibrosis [23]. BCL2-like 11 (BCL2L11) and C-C motif chemokine ligand 11 (CCL11), which are regulated by NFκB, are involved in liver inflammatory responses. Another study demonstrated that BCL2L11 is an inflammation amplifier [24]. CCL11 is involved in chronic inflammation, which facilitates the development of liver cirrhosis and fibrosis [25]. Very low-density lipoprotein receptor (VLDLR) and angiopoietin-like 4 (ANGPTL4), which are regulated by PPARγ, participate in lipid metabolism. VLDLR plays a crucial role in the clearance of triglyceride-rich lipoproteins from the circulation [26]. ANGPTL4 deficiency reportedly increases free cholesterol levels and promotes liver fibrosis in patients with NASH [27]. These genes were chosen as potential therapeutic targets for further validation.

### 2.5. Validation of Potential Therapeutic Targets of XYS and Its Eight Constituent Herbs

**Figure 7:**
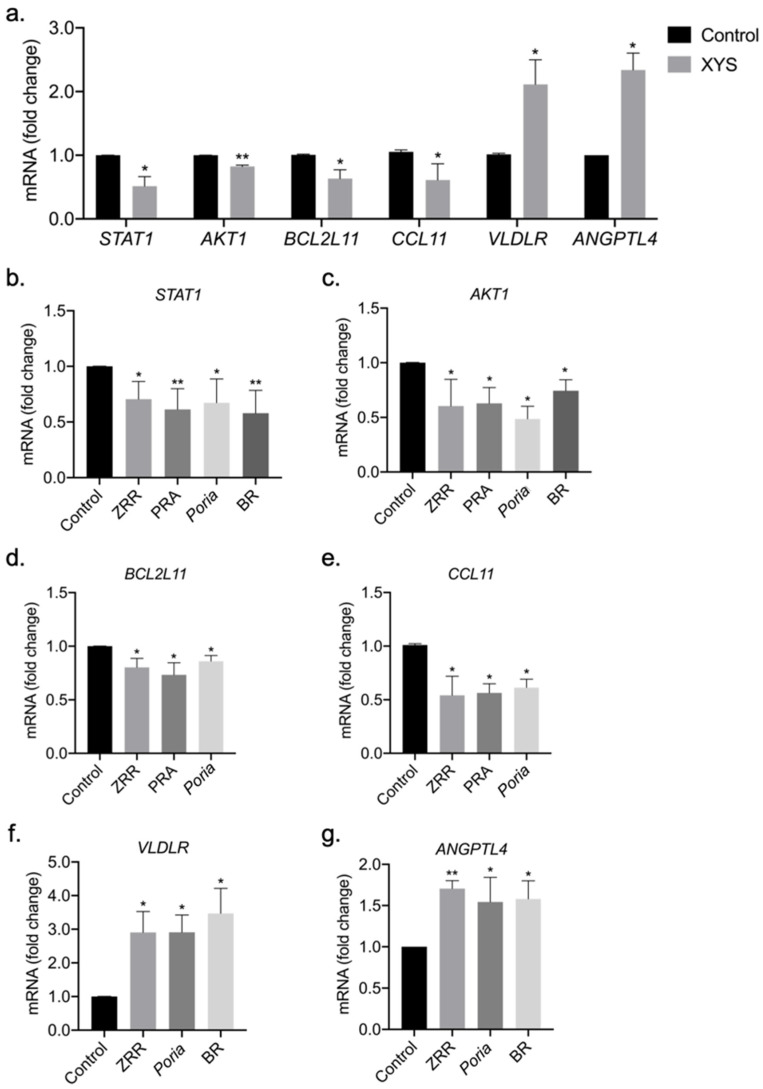
Validation of potential therapeutic targets of XYS and its constituent herbs: (**a**) *STAT1*, *AKT1*, *BCL2L11*, *CCL11*, *VLDLR*, and *ANGPTL4* mRNA expression in LX-2 cells treated with 400 μg/mL XYS for 24 h. (**b**,**c**) *STAT1* and *AKT1* mRNA expression in LX-2 cells treated with 100 μg/mL ZRR, PRA, *Poria*, or BR for 24 h. (**d**,**e**) *BCL2L11* and *CCL11* mRNA expression in LX-2 cells treated with 100 μg/mL ZRR, PRA, or *Poria* for 24 h. (**f**,**g**) *VLDLR* and *ANGPTL4* mRNA expression in LX-2 cells treated with 100 μg/mL ZRR, *Poria*, or BR for 24 h. The data are shown as the mean ± SD. Statistical analyses were conducted using paired Student’s *t* tests. * *p* < 0.05, ** *p* < 0.01 vs. the control group.

We treated LX-2 cells with XYS extract at 400 µg/mL, and then measured the mRNA levels of these potential therapeutic targets. The qRT-PCR results showed that XYS extract significantly downregulated the mRNA expression of *STAT1*, *AKT1*, *BCL2L11*, and *CCL11* and upregulated the mRNA expression of *VLDLR* and *ANGPTL4* (Figure 7a).

Based on Figure 3 and Figure 4, we aimed to further test the regulatory effects of BR, *Poria*, ZRR, and PRA, which can partially represent the function of XYS in the ECM, inflammation, and metabolism. Then, we treated LX-2 cells with 100 µg/mL BR, *Poria*, ZRR, or PRA extract and evaluated the ability of these extracts to regulate the expression of these genes. The qRT-PCR results showed that BR, *Poria*, ZRR, and PRA all significantly downregulated the mRNA expression of the profibrogenic genes *STAT1* and *AKT1* (Figure 7b,c). According to pathway enrichment analysis, *Poria*, ZRR, and PRA are strongly associated with inflammation, and we found that these three herbs significantly downregulated the mRNA expression of *BCL2L11* and *CCL11*, which are proinflammatory genes (Figure 7d,e). ZRR, *Poria*, and BR were shown to be related to metabolism via pathway enrichment analysis. Our results showed that these three herbs significantly upregulated the mRNA expression of *VLDLR* and *ANGPTL4*, which are lipid-regulating factors (Figure 7f,g). These results revealed that XYS exerts its therapeutic effect on NASH-related liver fibrosis by regulating the expression of profibrogenic genes, proinflammatory genes, and metabolism-related genes.

### 2.6. Screening of Potential Active Compounds in XYS That Inhibit NASH-Related Liver Fibrosis

To further investigate the potential active compounds in XYS, molecular docking was performed to predict the candidate effective compounds. First, we obtained the data for the compounds in BR, PRA, *Poria*, and ZRR from the online TCM databases TCMSP, ETCM, TCMID, and HIT 2.0. To ensure the accuracy of the results, compounds for which the 3D conformer could not be determined were excluded, and 487 compounds in BR, 153 compounds in PRA, 81 compounds in *Poria*, and 609 compounds in ZRR were assessed for their ability to bind to STAT3, NFκB, and PPARγ, respectively. A ligand with a lower binding energy score has a greater binding capability with the active site of the target protein.

**Figure 8:**
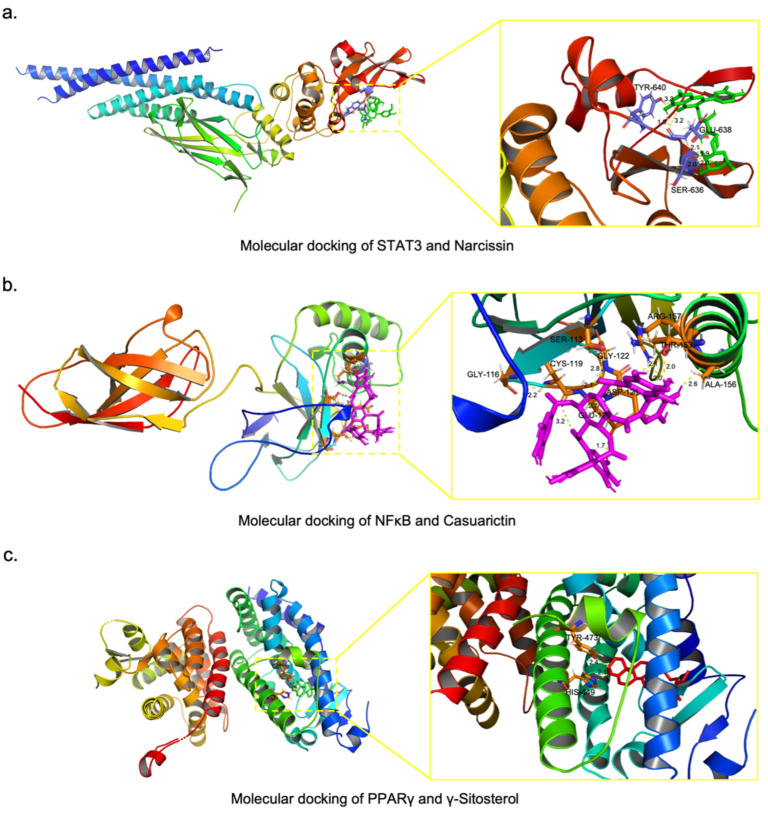
Molecular docking models of the target proteins and compounds: (**a**) Model of narcissin binding to STAT3. The green stick represents narcissin. (**b**) Model of casuarictin binding to NFκB. The pink stick represents casuarictin. (**c**) Model of γ-sitosterol binding to PPARγ. The red stick represents γ-sitosterol. All the coordinate bonds are indicated as yellow dotted lines.

First, we calculated the binding energies of the compounds in BR, PRA, *Poria*, and ZRR to STAT3. A compound with a lower binding energy than that of the positive control was considered to have a good binding affinity for STAT3. Table 2 lists the compounds in BR and PRA with the five lowest binding energies and a total of four compounds in ZRR with lower binding energies than the positive control. The compounds in *Poria* showed no binding ability to STAT3. The top 5 compounds with the lowest binding energies in the BR treatment were narcissin, rutin, saikosaponin B, saikosaponin A, and stigmasterol glucoside. The top 5 compounds with the lowest binding energies in PRA were 1,2,4,6-tetragalloylglucose, tellimagrandin II, tellimagrandin I, 1,2,6-tri-O-galloyl-β-D-glucose, and 1,2,3-tri-O-galloyl-β-D-glucose. A total of four compounds in ZRR, including theaflavin 3,3′-digallate, geraniin, eudesobovatol A, and isoginkgetin, exhibited good binding to STAT3. Taken together, these findings revealed that the narcissin in BR, 1,2,4,6-tetragalloylglucose in PRA, and theaflavin 3,3′-digallate in ZRR are the most potentially active compounds responsible for the therapeutic effects against liver fibrosis by targeting STAT3. Among these compounds, narcissin has the lowest binding energy to STAT3. The binding model of narcissin and STAT3 is shown in Figure 8a. The structure of narcissin forms three hydrogen bonds with TYR-640, GLU-638, and SER-636 in STAT3.

Next, we calculated the binding energies of the compounds in PRA, ZRR, and *Poria* to NFκB. With the same standard, the compounds in each herb with the five lowest binding energies are listed in Table 3. The top 5 compounds with the lowest binding energies in PRA were casuarictin, eugeniin, 1,2,6-trigalloylglucose, peonidin-3,5-O-di-beta-glucopyranoside, and 1,2,3-tri-O-galloyl-beta-D-glucose. The top 5 compounds with the lowest binding energies in ZRR were geraniin, theaflavin 3,3′-digallate, rutin, eudesobovatol A, and isoginkgetin. The top 5 compounds with the lowest binding energies in *Poria* were 25-hydroxy-3-epidehydrotumulosic acid, 3-epidehydrotumulosic acid, avicularin, dehydrotumulosic acid, and tumulosic acid. Overall, these results indicated that, among the putative active compounds, casuarictin in PRA, geraniin in ZRR, and 25-hydroxy-3-epidehydrotumulosic acid in *Poria*, which have the lowest binding energy, have anti-inflammatory effects by targeting NFκB. Among these compounds, casuarictin has the lowest binding energy to NFκB. The binding model of casuarictin and NFκB is shown in Figure 8b. NFκB and casuarictin bind to ALA-156, GLY-116, CSY-119, ASP-121, GLY-122, SER-113, GLU-120, ARG-157, and THR-153 through nine hydrogen bonds.

Finally, we calculated the binding energies of the compounds in BR, *Poria*, and ZRR to PPARγ. As shown in Table 4, a total of four compounds in BR and two compounds in *Poria* had a lower binding energy to PPARγ compared to the positive controls; these included γ-sitosterol, poriferasterol, baicalin, and cubebin in BR and turanose and methyl dehydroabietate in *Poria*. The top 5 compounds with the lowest binding energies in ZRR were γ-sitosterol, β-sitosterol, gingerenone B, dihydrocurcumin, and gingerenone A. According to these results, we considered γ-sitosterol in BR and ZRR and turanose in *Poria* as the most likely active compounds that exert metabolic regulatory effects by targeting PPARγ. γ-Sitosterol has the lowest binding energy among these compounds. Figure 8c shows the binding model of γ-sitosterol and PPARγ, and γ-sitosterol formed two hydrogen bonds with TYR-473 and HIS-449 in PPARγ.

## 3. Discussion

In the present study, the mechanism by which XYS and its constituent herbs treat NASH-related liver fibrosis was studied in a distinct way via integrated network pharmacology and transcriptome analyses. Our study proposes a new strategy to elucidate the mechanism of a TCM formula, enhancing our comprehension of TCM theory.

Different from previous studies, we first reported the action mechanism of XYS constituent herbs. Our research revealed the therapeutic effect of XYS on NASH-related liver fibrosis, which involves multiple pathways associated with the ECM, inflammation, and metabolism, and the eight constituent herbs of XYS work in concert to exert its therapeutic effects. Subsequently, STAT3, NFκB, and PPARγ were identified as the potential therapeutic targets of XYS. XYS and its constituent herbs ZRR, PRA, *Poria*, and BR showed strong regulatory effects on these three targets and regulated the expression of their downstream genes.

ZRR has been widely used as an herb and spice, which has rich bioactivities. Studies found that ZRR and its active compounds are bioactive, inducing cancer cell apoptosis by inhibiting STAT3 and NFκB, repressing inflammation by inhibiting NFκB, and fighting obesity by upregulating PPARγ [28]. PRA has been used to treat inflammation and immune disorders. Pharmacological studies of its main active compound, paeoniflorin, showed that paeoniflorin has anti-inflammatory and immunoregulatory effects via inhibiting NF-κB and STAT3 [29]. *Poria* is an edible herb that exhibits various biological activities. Studies indicated the bioactive compounds triterpenoid and polysaccharide of *Poria* showed an anti-inflammatory effect by reducing the secretion of pro-inflammatory factors, such as NFκB and STAT3 [30,31]. In addition, Sun et al. found oral administration of the polysaccharide of *Poria* significantly improved lipid metabolism and alleviated hepatic steatosis in ob/ob mice via activating the PPARγ pathway [32]. BR is one of the most important herbal medicines in TCM. Yuan et al. reviewed the pharmacological studies of BR and found that saikosaponins, the main active compound of BR, possess an anti-inflammatory effect by inhibiting the activity of STAT3 [33]. Saikosaponin A was reported to regulate lipid transportation by upregulating the expression of PPARγ [34]. Our study revealed that ZRR, PRA, *Poria*, and BR could treat NASH-related liver fibrosis based on their antifibrotic, anti-inflammatory, and metabolic regulatory effects.

Moreover, we predicted that narcissin from BR, casuarictin from PRA, and γ-sitosterol from BR and ZRR, are the active compounds in XYS responsible for its therapeutic effect on NASH-related liver fibrosis, through targeting STAT3, NFκB, and PPARγ, respectively.

Narcissin is a flavonol glycoside, and a study demonstrated that narcissin has antimicrobial and anti-inflammatory effects [35]. Casuarictin is an ellagitannin, and studies reported that casuarictin exhibits anti-inflammatory properties by inhibiting the NF-κB pathway [36,37]. An in vivo study demonstrated that γ-sitosterol (also known as clionasterol) has antihyperglycemic activity in an STZ-induced diabetic rat model [38]. Several in silico studies have suggested that γ-sitosterol exerts PPARγ agonistic properties, which is effective against obesity and alcoholic liver disease [39,40]. These reports support our conclusion that the three compounds may possess therapeutic effects on NASH-related liver fibrosis. The interaction between the potential active compounds with the therapeutic targets still needs further verification.

This study has some limitations as well. For example, the efficacy and mechanism of XYS in treating NASH-related liver fibrosis were only investigated at the cellular level. Animal studies and clinical trials of XYS are necessary to evaluate its efficacy and long-term use safety in future work.

## 4. Materials and Methods

### 4.1. Design of the Study

This study is mainly divided into three parts: transcriptome profile construction, network pharmacology analysis, and validation. Firstly, we treated the LX-2 cells with the extracts of XYS and its 8 constituent herbs, respectively, and conducted HTS^2^ to construct the signature gene expression profiles. Subsequently, after KEGG pathway enrichment analysis, protein–protein interaction network construction, and transcription factor enrichment analysis, the potential therapeutic targets were identified. Finally, the qRT-PCR and molecular docking analysis were performed to verify the potential therapeutic targets and identify the potential active compounds in XYS.

### 4.2. Cell Culture

The LX-2 cell line was purchased from Procell (Wuhan, China). LX-2 cells were cultured in high-glucose Dulbecco’s modified Eagle’s medium (DMEM) obtained from HyClone (Logan, UT, USA) supplemented with 10% (*v*/*v*) fetal bovine serum (FBS) obtained from ExCell Bio (Suzhou, China), 100 units/mL penicillin, and 100 μg/mL streptomycin (HyClone). The cells were incubated at 37 °C in a humidified atmosphere with 5% CO_2_.

### 4.3. Extraction of XYS and Its Constituent Herbs

The constituent herb of XYS was purchased from Anguo Changda Chinese Herbal Pieces Ltd. (Anguo, China). The TCMs were ground into powder, weighed (10 g), extracted by the heating reflux method with 150 mL of 90% (*v*/*v*) ethanol (3 h), concentrated (20 min), and lyophilized (24 h). The amount of each herb in XYS was normalized to a total weight of 10 g while keeping the ratio of each constituent unchanged, and these herbs were also extracted in accordance with the protocol described above. Finally, the lyophilized powders were dissolved in dimethyl sulfoxide (DMSO) to a concentration of 50 mg/mL.

### 4.4. HTS^2^

LX-2 cells were seeded in 384-well plates (Corning, Inc., Corning, NY, USA) at a density of 2500 cells per well and incubated for 24 h. Then, the cells were treated with 100 μg/mL XYS, each herbal extract or DMSO for 24 h. The cells were lysed, and RASL was subsequently added as previously described [41]. In brief, total RNA in the samples was denatured by heating at 65 °C for 8 min, and the designed probes were subsequently annealed to the RNA at 45 °C for 60 min. A pair of probes for each of the 4141 genes was designed based on the exon–exon junction near the 3′ end of each gene as previously described. The corresponding mRNAs were captured by magnetic beads with a biotinylated oligo (dT) strand. After being washed, the probe pairs were ligated with T4 DNA ligase at 37 °C for 60 min, after which the target sequences were amplified via polymerase chain reaction (PCR) with a set of barcoded primers. The amplification products were collected, purified, quantified, and subjected to high-throughput PE150 sequencing using the HiSeq Xten platform (Illumina, San Diego, CA, USA). To assess the repeatability and reliability of the HTS^2^ assay data, 3 replicates of each herb and XYS and 16 replicates of DMSO were included in a single 384-well plate. The sequencing reads were mapped with Bowtie2, after which quality control was conducted with a cutoff correlation coefficient of ≥ 0.8 for the herbs and XYS and 0.9 for the solvent controls (DMSO). Next, the differentially expressed genes (DEGs) were identified using DESeq2 with the criteria of a fold change ≥ 1.5 or ≤ 0.67 and *p* < 0.05.

### 4.5. Kyoto Encyclopedia of Genes and Genomes (KEGG) Pathway Enrichment Analysis

KEGG pathway enrichment analysis was subsequently performed on the genes differentially expressed in response to XYS and herb treatment. KEGG pathway enrichment analysis was performed via the Database for Annotation, Visualization, and Integrated Discovery (DAVID) tool [42]. The significantly enriched terms were identified with the criterion of *p* < 0.05. A bubble plot was generated with the ggplot2 package of R 4.2.1.

### 4.6. Construction of the Protein–Protein Interaction (PPI) Network

A PPI network of the proteins encoded by the genes differentially expressed in response to XYS stimulation was constructed by using the Search Tool for the Retrieval of Interacting Genes/Proteins (STRING) 12.0 database [43].

### 4.7. Construction of the Gene–Transcription Factor (TF) Regulatory Interaction Network

TF enrichment analysis was performed by using the Transcriptional Regulatory Relationships Unraveled by Sentence-based Text mining (TRRUST) database [44]. The resulting gene–TF regulatory interaction network was visualized with Cytoscape 3.8.0 [45]. The TF binding motif sequence logos were obtained in HOMER [46].

### 4.8. Cell Viability Assay

LX-2 cells were seeded in 96-well plates at a density of 1.0 × 10^4^ cells per well and treated with different concentrations of XYS, herbs, or DMSO for 24 h. Then, 100 μL of CCK-8 reagent (Dojindo Molecular Technologies, Kumamon, Japan) was added to each well, and the cells were incubated for 2 h at 37 °C. The absorbance was measured at 450 nm using a SpectroMax spectrophotometer (Spectromax Solutions Ltd., London, UK). The cell viability rate was calculated as follows: (OD__Experiment_ − OD__Blank_)/(OD__Control_ − OD__Blank_) × 100%.

### 4.9. Quantitative Reverse Transcription Polymerase Chain Reaction (qRT-PCR)

Total RNA was isolated using an RNA Mini Kit (QIAGEN, Dusseldorf, Germany) and reverse transcribed using a High-Capacity RNA-to-cDNA Kit (Thermo Fisher Scientific, Rochester, NY, USA). qRT-PCR was conducted using the KAPA SYBR FAST qPCR Master Mix (2×) Kit (Kapa Biosystems, Woburn, MA, USA) on an Applied Biosystems 7500 Real-Time PCR system (Thermo Fisher). Each measurement was repeated in triplicate, and target mRNA levels were normalized to the mRNA level of glyceraldehyde 3-phosphate dehydrogenase (GAPDH). Relative gene expression was calculated by the following equation: fold change = 2^−ΔΔCT^, where ΔΔCT = ΔCT__Sample_ − ΔCT__Control_ and ΔCT = average CT__Test gene_ − average CT__GAPDH_ (CT, cycle threshold). The primers used in this study are listed in Table S1.

### 4.10. Molecular Docking

The crystal structures of STAT3 (PDB ID: 6NJS), NFκB (PDB ID: 1SVC), and PPARγ (PDB ID: 4EMA) were obtained from the Protein Data Bank https://www.pdbus.org/ (accessed on 31 October 2023). The active chemical ingredients in BR, PRA, *Poria*, and ZRR were obtained from online TCM databases, including the Traditional Chinese Medicine Systems Pharmacology (TCMSP) database, the Encyclopedia of Traditional Chinese Medicine (ETCM), the Traditional Chinese Medicine Integrated Database (TCMID) and the HIT 2.0 [47,48,49,50]. AutoDock Vina software 4.2.6 was used to identify the candidate STAT3 inhibitor, NFκB inhibitor, and PPARγ agonist among the chemical ingredients [51].

### 4.11. Statistical Analysis

All data were expressed as the mean ± standard deviation (SD), and paired, two-tailed Student’s *t*-test analysis was conducted using GraphPad Prism 9.0 software (GraphPad Software, San Diego, CA, USA). A *p* value < 0.05 was considered statistically significant.

## 5. Conclusions

In summary, we used a high-throughput drug screening platform to elucidate the therapeutic mechanism of XYS in NASH-related liver fibrosis. By combining transcriptome analysis with network pharmacology analysis, we demonstrated that the therapeutic mechanism of XYS in NASH-related liver fibrosis involves the regulation of STAT3, NFκB, and PPARγ and their downstream genes and the intervention of the ECM, inflammation, and metabolism. We predicted that the potential active compounds in XYS exert therapeutic effects by targeting STAT3, NFκB, and PPARγ based on molecular docking. This study provided a new research template for understanding the mechanisms of a complex TCM formula.

## Figures and Tables

**Table 1:** DEGs number of LX-2 cells in response to the treatment of XYS and 8 constituent herbs.

Pinyin Name	Latin Name	Number of DEGs		
		Up- Regulated	Down- Regulated	Total
XYS	\	574	342	916
Chai Hu	*Bupleuri Radix*	428	170	598
Dang Gui	*Angelicae Sinensis Radix*	311	53	364
Bai Shao	*Paeoniae Radix Alba*	184	119	303
Bai Zhu	*Atractylodis Macrocephalae Rhizoma*	558	69	627
Fu Ling	*Poria*	182	77	259
Sheng Jiang	*Zingiberis Rhizoma Recens*	515	250	765
Bo He	*Menthae Haplocalycis Herba*	31	180	211
Zhi Gan Cao	*Glycyrrhizae Radix Et Rhizoma Praeparata Cum Melle*	342	69	411

**Table 2:** Molecular docking results of compounds to STAT3.

Source Herb	Chemical Name	CAS No.	Binding Energy (Kcal·moL^−1^)
\	Positive control	\	−7.79
BR	Narcissin	604-80-8	−13.53
BR	Rutin	153-18-4	−10.67
BR	Saikosaponin B	58558-08-0	−10.49
BR	Saikosaponin A	20736-09-8	−10.48
BR	Stigmasterol glucoside	19716-26-8	−10.19
PRA	1,2,4,6-Tetragalloylglucose	84297-49-4	−12.06
PRA	Tellimagrandin II	81571-72-4	−12.04
PRA	Tellimagrandin I	79786-08-6	−11.61
PRA	1,2,6-Tri-O-galloyl-β-D-glucose	79886-49-0	−11.55
PRA	1,2,3-Tri-O-galloyl-β-D-glucose	84415-91-8	−11.51
ZRR	Theaflavin 3,3′-digallate	30462-35-2	−10.81
ZRR	Geraniin	60976-49-0	−10.45
ZRR	Eudesobovatol A	125196-77-2	−9.42
ZRR	Isoginkgetin	548-19-6	−8.74

**Table 3:** Molecular docking results of compounds to NFκB.

Source Herb	Chemical Name	CAS No.	Binding Energy (Kcal·moL^−1^)
\	Positive control	\	−6.15
PRA	Casuarictin	79786-00-8	−12.64
PRA	Eugeniin	58970-75-5	−10.97
PRA	1,2,6-Trigalloylglucose	79886-49-0	−10.85
PRA	Peonidin-3,5-O-di-beta-glucopyranoside	47851-83-2	−10.66
PRA	1,2,3-Tri-O-galloyl-beta-D-glucose	84415-91-8	−10.61
ZRR	Geraniin	60976-49-0	−12.03
ZRR	Theaflavin 3,3′-digallate	30462-35-2	−10.71
ZRR	Rutin	153-18-4	−10.63
ZRR	Eudesobovatol A	125196-77-2	−9.30
ZRR	Isoginkgetin	548-19-6	−8.76
*Poria*	25-hydroxy-3-epidehydrotumulosic acid	167775-55-5	−7.91
*Poria*	3-Epidehydrotumulosic acid	167775-54-4	−7.87
*Poria*	Avicularin	572-30-5	−7.47
*Poria*	Dehydrotumulosic acid	6754-16-1	−7.42
*Poria*	Tumulosic acid	508-24-7	−7.35

**Table 4:** Molecular docking results of compounds to PPARγ.

Source Herb	Chemical Name	CAS No.	Binding Energy (Kcal·moL^−1^)
\	Positive control	\	−7.84
BR	γ-Sitosterol	83-47-6	−9.60
BR	Poriferasterol	481-16-3	−9.51
BR	Baicalin	21967-41-9	−8.61
BR	Cubebin	18423-69-3	−8.09
*Poria*	Turanose	547-25-1	−9.45
*Poria*	Methyl dehydroabietate	1235-74-1	−8.12
ZRR	γ-Sitosterol	83-47-6	−9.60
ZRR	β-Sitosterol	83-46-5	−9.26
ZRR	Gingerenone B	128700-98-1	−8.67
ZRR	Dihydrocurcumin	76474-56-1	−8.57
ZRR	Gingerenone A	128700-97-0	−8.48

## Data Availability

Data are contained within the article and Supplementary Materials.

## Supplementary Materials

The following supporting information can be downloaded at: https://www.mdpi.com/article/10.3390/ph17091128/s1, Figure S1: Cell viability assay; Table S1: Primers used for quantitative Real-Time PCR; Table S2: DEGs of XYS vs. DMSO treatment; Table S3: DEGs of XYS composed 8 herbs vs. DMSO treatment.
