# Supplementary material for: Mechanism Study of Xiaoyao San against Nonalcoholic Steatohepatitis-Related Liver Fibrosis Based on a Combined Strategy of Transcriptome Analysis and Network Pharmacology
Source: Pharmaceuticals (Basel). 2024 Aug 27;17(9):1128. doi: 10.3390/ph17091128 (PMC11434732; doi:10.3390/ph17091128)
Supplement: Supplementary file 1 [file pharmaceuticals-17-01128-s001.zip › pharmaceuticals-3116660-supplementary.pdf]

Supplementary Materials for

Mechanism Study of Xiaoyao San Against Nonalcoholic Steatohepatitis-related Liver Fibrosis Based on a Combined Strategy of Transcriptome Analysis and Network Pharmacology

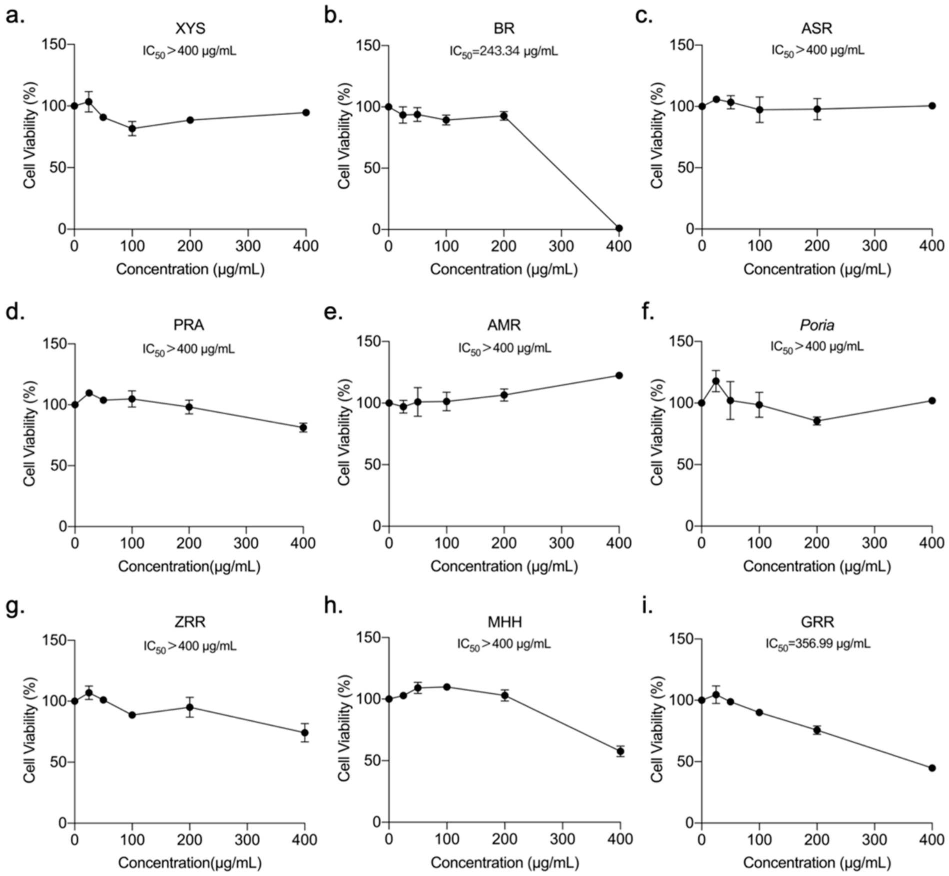

Figure S1. Cell viability assay. **(a-i)** Cell viability of LX-2 cells treated with XYS, BR, ASR, PRA, AMR, *Poria*, ZRR, MHH, or GRR, for 24 h.

Table S1. Primers used for quantitative Real-Time PCR

mRNAs	Forward primer (5'-3')	Reverse primer (3'-5')
ANGPTL4	GGCTCAGTGGACTTCAACCG	CCGTGATGCTATGCACCTTCT
AKT1	GTCATCGAACGCACCTTCCAT	AGCTTCAGGTACTCAAACTCGT
BCL2L11	TAAGTTCTGAGTGTGACCGAGA	GCTCTGTCTGTAGGGAGGTAGG
CCL11	CCCCTTCAGCGACTAGAGAG	TCTTGGGGTCGGCACAGAT
COL1A1	GAGGGCCAAGACGAAGACATC	CAGATCACGTCATCGCACAAC
COL3A1	GCCAAATATGTGTCTGTGACTCA	GGGCGAGTAGGAGCAGTTG
GAPDH	GGAGCGAGATCCCTCCAAAAT	GGCTGTTGTCATACTTCTCATGG
STAT1	CAGCTTGACTCAAAATTCCTGGA	TGAAGATTACGCTTGCTTTTCCT
TGFB1	CAATTCCTGGCGATACCTCAG	GCACAACTCCGGTGACATCAA
VLDLR	AGAAAAGCCAAATGTGAACCCT	CACTGCCGTCAACACAGTCT

Table S2. DEGs of XYS vs. DMSO treatment.

Gene_Symbol	Log_2FC
MFSD11	3.903
SIRT2	3.237
MYD88	3.753
C6	4.283
EOMES	5.858
PTPRC	5.085
XPA	3.536
SFN	4.951
G6PC3	4.636
FDFT1	3.647
TBXA2R	3.934
CACNG5	5.888
TCL1A	5.070
IL2RB	2.521
PRSS1	2.254
GPR35	4.169
BHLHE40	5.242
GPT	5.016
MTARC1	5.888
HTR1D	3.357
ASPH	3.962
TTN	3.842
GABRA3	3.993
IRF6	4.515
SLC4A2	3.565
ACVR2A	2.834
ADCY4	4.282
FHIT	4.508
CHRNA10	3.098

LPAR4	4.496
PVRL2	2.406
CISH	3.514
VEGFA	3.409
DSG2	4.537
A2M	3.585
ADCY5	3.147
HTR1E	3.215
PLA2G10	5.070
HCLS1	4.568
TSLP	3.895
APOD	4.377
AQP4	3.027
G6PC2	3.612
TUBA8	3.716
TNR	3.112
TRIM32	4.297
ATRX	4.290
QPRT	5.519
GHSR	5.303
PRDM2	3.934
PTGDR2	3.564
NPBWR2	4.883
MAP2K7	4.729
GALNT6	4.644
VLDLR	3.882
RAPGEF3	3.967
FCER1A	3.839
HCRTR2	4.652
HSD3B2	4.422
CCL11	4.513
TLR2	7.199
SCN4B	3.535
TJP3	4.763
FGF14	2.583
NPPC	3.856
PDE4D	2.656
PYDC1	2.527
IFNA10	2.648
CD1C	3.371
TJAP1	2.922
DTX3L	5.017
RAD52	3.155
RAB11B	0.870

CRHR1	0.857
ATRIP	1.577
DCTN1	1.310
CYTB	1.491
THBS1	1.415
CEBPB	1.152
FTH1	1.354
SOCS3	-1.019
NRP1	0.831
COX5B	0.997
HEXB	0.876
INHBA	0.964
DDIT3	-1.762
GBP2	3.649
ATP2A3	3.305
CD79A	7.126
APOL3	8.006
GDF5	7.371
TLX3	4.853
TLR1	3.867
ITGA7	4.591
PIK3CG	3.949
SPHK2	4.520
PRLHR	5.108
TICAM1	3.675
HLA-F	4.557
CACNG3	4.291
CHRM2	3.267
AK4	4.331
DTX1	3.965
ISL1	3.239
TAAR1	7.027
RYR3	7.755
MMP8	7.077
TNFSF8	5.947
FSHR	4.822
PRKN	4.535
ENAH	3.982
GHRL	4.272
TRPV5	3.282
SSTR3	4.739
FLG	4.339
FAS	4.560
PPP5D1	4.112

IFNGR1	3.744
PTK2B	3.877
CD81	2.868
PLD3	2.421
RCHY1	3.069
CAMSAP1	3.659
GNAQ	3.127
IKBKG	2.909
BNIP3	3.164
TAOK2	3.415
LEF1	1.367
PIK3R4	2.392
PLCE1	2.452
CAV2	2.354
PTEN	2.456
RPS6KA3	2.654
MYBPH	3.887
SNW1	4.199
MTFP1	2.672
PRLR	3.464
ARHGEF11	4.439
PTPN6	2.878
NEIL3	3.257
NFAT5	3.901
PTGFR	2.592
NDUFC2-KCTD14	2.956
C3AR1	3.201
SUFU	1.580
AGTRAP	1.356
SCN1B	2.513
SLC9A3	3.769
PLIN4	3.978
LEPR	3.062
ACSM3	4.004
MDM2	3.968
TOX	2.911
ADRA1A	4.397
CTSW	2.893
CMTM4	4.224
P2RY8	4.101
HAS2	2.763
RDH10	3.996
EEF2K	3.116
NR1H4	3.602

CHIA	2.329
GPNMB	2.487
FBXO2	4.346
IFNA4	3.837
ATP6V1B1	3.870
DUSP8	3.009
OGDHL	4.132
KAT2B	3.605
ALDH18A1	2.892
MGAT4B	3.697
MAP4K2	2.731
TNNT2	3.630
TSC22D1	4.310
IL22RA2	5.048
TNFRSF10C	3.779
IL23A	4.312
CALB1	4.443
SPINK5	4.618
ALDH1A2	3.829
PPARGC1A	3.799
CSNK1G3	4.542
FSHB	3.962
APBB1IP	3.396
IFNG	3.889
PAG1	2.762
GRIA2	3.476
RIMS2	3.412
TNFSF13B	3.830
TXNL4B	2.691
IL20RB	3.067
TNFSF18	2.384
F5	3.318
PTGER3	3.846
IL18R1	2.903
CACNA1I	3.448
CACNA1S	3.074
CD40LG	3.515
ADORA3	3.853
IL20RA	2.716
LDHAL6A	3.715
NOSTRIN	3.111
DAPK1	3.452
SGCD	4.854
IDO1	3.323

MAML3	4.107
HCRTR1	5.054
P2RY13	4.694
F2RL1	3.855
MC4R	4.615
CD1B	4.991
RORC	3.150
LEP	4.480
GRM1	3.497
PDK4	3.107
MDH1B	2.825
PPP1R14A	2.416
DET1	4.635
SCD	3.126
CD47	3.016
GNGT2	2.366
BIRC3	2.351
PRKD2	2.052
ZFP91	1.513
TIMP2	2.393
STK24	2.491
HMGB1	3.134
HSPA4	3.590
JAK2	2.700
TSNAX	2.439
CST3	2.516
ATP6V1A	2.367
GNAI1	2.751
GANAB	2.752
TRIP12	2.342
ENO3	3.671
LMNA	1.697
NFYA	2.651
FLT4	3.634
NTRK1	3.624
LDHA	2.480
PLCG1	4.145
MRC1	5.475
GCG	4.945
GUCY1A2	2.760
IFIT1	3.344
HMOX1	2.776
ITGA4	4.335
MASP1	2.868

GPR161	3.234
NCOA3	2.358
HOXB1	3.051
CAMK1D	4.543
GFAP	4.332
ZNF281	3.955
AOC3	3.244
PSTS	4.669
HCN4	3.883
ATM	3.650
PPL	2.570
BMP2	3.509
ACADM	2.068
RPA3	2.453
PDE2A	4.395
CAMK1G	3.734
RHOJ	2.885
SOCS5	4.119
CFI	3.196
SP110	3.039
PPAT	3.084
NOLC1	2.438
ANXA1	2.406
STRADA	3.171
SLC27A4	3.145
GALNT3	3.938
KIF7	3.225
ADAMTS1	2.490
TNFSF11	4.523
PSME1	3.099
WARS	4.149
CYP3A5	3.768
ENPEP	4.236
TRIM10	4.435
TADA3	4.895
GAS1	3.984
MARK2	3.094
AGL	3.909
AQP1	4.221
ADIPOQ	4.438
CXADR	7.299
SETBP1	7.973
GSTO2	4.377
ENO4	6.396

PRKCB	4.868
CNGA4	4.923
ESR2	3.804
GABRE	3.669
SLC27A2	2.965
FOXD3	5.024
GABRR1	3.874
ISG20	5.907
VAV1	5.464
APOA2	5.470
ANGPTL4	4.518
MSTN	4.101
MFNG	6.876
BLNK	5.816
CALCB	5.339
FZD9	5.051
PPM1D	4.988
CLDN4	6.208
DUSP9	6.176
NLRC5	6.078
GNRH2	4.855
NUMBL	4.820
NPFF	6.856
COX4I2	3.771
EGF	5.341
KCNMB1	4.531
DTX3	3.901
SCARF1	4.117
TRH	5.600
VIPR2	6.400
HLA-C	8.006
CD1D	8.006
NDUFB8	5.325
KCNJ8	4.413
C8A	4.685
OXCT2	4.478
PRR5	4.400
TIRAP	4.455
MYO1C	4.348
SV2A	4.366
ADRB2	4.154
SDC1	4.002
EGR2	4.681
GABRP	4.761

PPP2R2C	4.947
CHRM4	4.872
NFYB	4.827
KLK2	5.200
TGFBR2	5.490
CYP8B1	5.531
IL18RAP	5.527
CRAT	3.787
MAPK1	3.336
SLC51B	3.711
MDM4	3.750
IL4R	3.590
UCN	3.664
PTHLH	3.112
C1GALT1C1	3.017
NFE2L2	2.996
MAPKAPK5	3.483
KLHL13	3.237
DOK1	3.330
SOCS7	3.355
PVRL1	3.357
NLRX1	3.709
CD24	3.025
CYP51A1	2.428
CDK8	3.745
IRF5	5.108
FXYD4	5.127
ABCB4	6.031
GABBR2	6.659
EDN3	8.199
NPFFR2	8.006
ATP1B4	7.977
SCGB1A1	7.977
EPHA7	7.299
GRIN2B	7.421
NPPA	7.743
VAV3	7.563
FGF1	7.521
AVPR1B	7.077
PAX4	6.977
STRA6	7.006
CXCR6	6.784
ARG1	6.784
ROR1	6.687

SOCS6	6.601
GRP	5.784
CNGA3	5.755
COL9A2	5.732
GPR83	5.939
UTS2	5.873
IL17RE	5.831
HLA-DRB5	6.087
SLC27A3	6.110
TNFSF13	6.111
IL27	6.281
SH2B1	6.340
CHRNB2	5.399
PDE1A	5.542
AGGF1	3.730
VGLL4	3.917
GSTM4	2.022
LRP12	2.462
PANX2	1.793
AKR1B1	1.604
ATG4D	1.805
IFITM2	1.916
ATP6V0A1	1.849
MAPK12	1.230
WNT5A	0.958
DUSP14	1.242
CTTN	0.862
TGFB1	0.727
ACTN4	0.648
RAB4A	0.996
MAPK9	0.980
RALA	0.968
BOD1	0.967
ATP2A2	0.866
DYNC1LI1	0.916
RBM23	1.488
ACLY	1.541
MPP6	1.283
NFKBIE	1.329
AIFM1	1.345
POLD2	1.844
MLX	1.827
FTSJ1	1.658
RAC1	1.540

DNM1	2.540
APAF1	2.049
AP2B1	2.509
FAT1	2.368
ATP5G2	2.218
SDAD1	1.916
APH1A	2.179
DUSP3	2.150
SCRIB	2.133
GLRX	2.138
EPHX1	2.079
CAD	2.060
E2F4	2.330
ARHGAP35	2.435
LAMA1	2.550
MUC1	2.666
MVK	2.672
RHOBTB2	2.446
USP5	2.713
IDH3B	1.830
PDE5A	1.879
RAP1A	2.041
UBE2B	1.197
SPATA2	1.707
OCLN	2.794
ATP2B1	1.639
MIF	1.406
TYK2	1.222
NDUFS4	1.116
CNOT2	1.175
AP2M1	1.546
ILF2	1.900
MFGE8	2.154
CLDN12	1.737
CHUK	1.006
NDUFA6	0.792
BBC3	1.250
COL18A1	1.635
FER	3.326
RHOB	2.407
ATG4B	2.034
ARRB2	1.987
NDUFAB1	2.452
DUSP4	4.534

SHC2	4.409
ITGAE	3.837
AIM2	3.555
GNG7	3.120
NLK	3.326
EPB41	3.366
PRSS2	3.410
DYNC2H1	1.964
MAP3K2	2.077
PECR	2.474
ORAI1	1.586
PIK3AP1	3.183
SMAD3	1.678
PLIN2	3.367
SQSTM1	1.942
SDHB	2.778
PRC1	2.344
CARD11	3.220
FBXO4	2.543
PVR	2.467
SLC39A6	2.345
ATG4A	2.192
BECN1	1.796
EIF3M	1.648
DOCK1	1.979
PTPRF	1.802
LFNG	2.085
SORT1	2.021
NOTCH1	3.080
EFNB1	2.635
APIP	3.511
TFPI	3.294
ATG10	4.019
ALDH1A1	4.133
CLDN15	4.721
PDE10A	3.872
PDX1	5.597
HLA-DMA	4.096
SERPIND1	4.690
SPATA20	3.663
VSIG4	4.988
NMB	3.474
TFF3	4.048
C5AR1	5.606

AGER	3.232
ABCC3	3.902
PGLS	2.579
ACAD10	3.126
MMP15	5.035
CBLC	5.384
TNFRSF14	6.246
CTLA4	3.001
OAT	2.425
PTTG1	4.490
ERBB3	7.340
KRT5	5.088
RAMP1	3.201
SMAD6	3.802
LAMTOR1	3.443
ADCY7	3.840
MAP2K5	3.761
PLCD3	-2.280
IL7R	-2.239
YY1	-3.116
GNG4	-1.697
APP	-2.267
ATF1	-1.821
DYNC1I2	-1.444
PPP1R12A	-1.081
BNIP2	-1.549
ERBB2IP	-1.229
SOS2	-1.187
FGF5	-1.476
CAV1	-1.749
AMOTL2	-1.767
DLC1	-0.828
LTBP1	-1.097
BCL10	-1.097
STMN1	-1.440
KIF5B	-1.510
UBE2Q2	-1.560
EIF2S1	-2.080
SAE1	-1.426
RAB10	-1.886
MYH10	-2.222
MAPRE2	-2.308
CTNND1	-2.364
HNRNPUL1	-2.171

PNN	-2.129
PDHA1	-2.440
KCNMA1	-2.160
CASC3	-1.710
SLC25A5	-2.022
DAG1	-2.780
WNT5B	-1.822
ARID5B	-2.826
CUL4B	-2.337
ANKRD1	-1.757
PRKACA	-1.581
DKK1	-1.461
FN1	-1.396
HIF1A	-2.036
MAN1A1	-4.093
PLK2	-2.609
PRKAA1	-1.634
GNG12	-1.870
MYBL1	-1.936
UBA6	-2.746
CREG1	-2.600
GLB1	-3.818
RALB	-3.036
ATP1B3	-2.574
RARRES2	-2.186
ILK	-1.752
CSDE1	-1.910
ZHX3	-2.805
GTF2I	-1.775
CDKN1B	-2.276
IP6K2	-2.172
TCF20	-3.364
IFI6	-1.988
SET	-1.845
CKLF	-2.092
CDK5R1	-2.314
SEH1L	-1.617
EMD	-1.339
RNF8	-1.481
PPP2CA	-2.096
IFRD2	-2.244
XPC	-1.248
XBP1	-1.466
CDK1	-1.360

FEN1	-1.382
NDUFA8	-1.382
USP7	-1.998
POLR2F	-1.582
RAD1	-1.507
TERF1	-1.544
FXC1	-1.795
PES1	-1.691
HOMER3	-1.660
NDUFA2	-2.041
UBE2L6	-2.166
GBP1	-1.472
FZD2	-1.925
NOG	-2.386
PRKACB	-2.683
RBL1	-2.478
PPP1CC	-2.025
EIF4G3	-2.274
RAB2A	-3.013
SLIT2	-3.164
CLIP1	-1.960
DPYSL4	-2.429
FKBP5	-1.957
MGST3	-2.154
PIK3CB	-2.244
PHB	-2.608
SUMO2	-2.505
GABARAPL1	-2.508
DYNC1LI2	-2.955
NF2	-2.968
PRPS1	-3.172
CTNNBIP1	-3.100
FKBP1A	-3.642
ASH1L	-3.295
CFLAR	-3.177
VPS13A	-2.554
NXT2	-2.924
EPAS1	-3.010
SLK	-2.215
GFPT1	-2.248
SDC2	-1.956
STK38L	-2.091
PAK1	-2.107
DNM2	-2.499

OGG1	-2.867
BRCA1	-2.825
UBE2D1	-3.081
ATP6V1E1	-2.955
RIPK1	-2.973
GAPDH	-2.612
HRH1	-2.351
PKD2	-2.396
IFNGR2	-2.582
LAMTOR4	-2.473
SLC38A9	-2.474
LMNB2	-2.998
TAB2	-3.126
GJA1	-3.198
PTPN11	-3.372
MED1	-1.586
ZFYVE16	-1.679
TANK	-1.169
SMAD4	-0.872
PSIP1	-1.087
DYNLL1	-1.149
MAT2A	-0.738
RAB7A	-0.779
HUWE1	-0.913
PIN1	-1.047
UBE2A	-1.313
DCTN6	-1.103
TYMS	-1.491
PPP2R5A	-1.300
COX5A	-1.014
C1D	-1.716
STAT5B	-1.797
FUCA1	-1.641
RHEB	-1.640
RICTOR	-1.119
FUT8	-1.222
TAOK1	-1.801
PPP1R12B	1.492
XRCC1	2.741
CCNG2	2.240
EEA1	2.000
HOMER1	1.112
FZD6	1.686
ACVR1B	2.127

PSD3	1.890
ACAT1	1.400
E2F3	2.029
RAB5A	2.447
PRKD3	1.675
ITGA5	1.621
RNASEL	1.552
UBE2NL	0.861
GNG11	0.866
CHD4	-6.009
POFUT1	-4.196
ERCC1	-5.724
BIRC5	-5.351
RPS6KA6	-4.709
LAMC1	-4.770
UBE2E3	-4.643
PIK3CA	-4.745
NRIP1	-2.203
FYN	1.037
ITPKA	1.537
MAN2A1	1.528
HDLBP	1.274
PA2G4	1.530
DVL2	1.973
NIT2	1.664
NLN	1.539
BAD	1.095
B4GALT1	-1.401
SMURF2	-1.446
RPS5	-0.794
DOLK	0.801
ACTA2	0.726
NDUFB1	-0.622
SKP2	-5.982
CAPN2	-5.422
CS	-5.772
FES	-5.446
GNPTAB	-4.821
BCAT1	-5.786
USF1	-5.226
ACOX1	-4.158
RPA1	-3.230
SIRT1	-4.195
PCBD1	-4.291

MAP2K1	-5.623
PDPR	-4.747
COL8A1	-4.857
AURKA	-4.869
WFS1	-4.366
ING1	-4.490
TRIB3	-4.183
NDUFB4	-4.143
JAM3	-5.333
VASP	-4.310
ZFP36L1	-4.522
LIG1	-4.491
TAB3	-4.189
TBC1D7	-4.849
TLE1	-4.888
AJUBA	-5.265
ASAP1	-6.593
RASSF1	-5.997
ENO2	-5.734
STAT2	-5.845
TSC1	-4.675
UBE3B	-4.962
CSNK2B	-5.118
PGAM5	-5.696
PPP2R3B	-5.759
DAGLB	-5.436
SLC9A6	-5.195
MAN2B1	-5.287
TBC1D1	-5.576
CD99	-5.469
CYB5R3	-4.557
MAP3K5	-4.482
PDHB	-4.586
SH3GL1	-4.506
LDHB	-4.810
MSH6	-4.254
CCNO	-4.592
BID	-4.610
HNRNPA2B1	-4.551
MAZ	-4.500
PIAS3	-4.767
COX7A2	-4.911
SLC12A7	-4.981
AKT1	-4.966

PRPS2	-4.972
ALDOC	-4.731
SMAD2	-4.834
MAPK7	-4.587
ELAVL1	-4.117
STAT1	-4.328
POLD3	-5.160
PIK3CD	-4.670
GATA2	-4.221
TNFRSF12A	-4.551
PYGL	-4.776
NR3C2	-4.335
RELT	-4.478
UBA1	-4.322
TJP1	-3.909
CD59	-3.669
BCL2L11	-4.618
GUCY1B3	-5.189
LPAR1	-4.369
AHNAK	-4.045
ACTR2	-5.654
COL4A1	-5.416
SRSF1	-4.953
EIF4E	-4.895
ACADSB	-4.509
MAP3K3	1.174
SPTAN1	1.790
PPM1A	-1.074
CLTCL1	-1.888
SMAD9	-4.693
XRCC4	-4.409
SUCLA2	-3.113
MGAT5	-5.373
GABRQ	-4.445
DIABLO	-4.156
NFKB1	-4.775
MCCC2	-4.576
TGFBR3	-4.698
TUBB3	-4.790
UNG	-5.034
CASK	-5.251
LIMK1	-5.912
YWHAG	-4.420
BSG	-5.793

HERC3	-4.899
STK4	-4.448
BNIP3L	-4.658
OSMR	-4.891
RAD50	-5.391
PSMD5	-6.139
PDGFA	-4.457
JPH3	1.546
PNKP	1.862
KIF3A	1.884
EIF4G1	2.779
RPS6KB2	1.692
TNFSF9	2.098
EFNB2	1.627
MPG	2.281
SESN2	2.223
FOSL2	2.414
ACO1	2.775
COX1	-0.724
COL5A1	-0.818
TPM1	-1.334
ARHGDIB	-1.557
SPARC	-1.223
STX18	-1.505
IDI1	-1.339
B2M	-1.484
FGF2	-1.188
EZR	-0.994
GTSE1	-0.736
DAB2	-0.753
DLD	-0.850
ACTB	-1.256
MYL12A	-0.771
MSN	-0.902
HNRNPA1	-0.600
XRCC6	-0.602
PRMT1	-0.624
NDUFA7	-0.736
SUMO3	-0.693
LAMTOR5	-0.680
UBE2K	-0.680
PEA15	-0.652
NDUFS6	-0.662
RPL13	-0.667

COX8A	-0.977
NSDHL	-1.032
CALM	-1.032
YWHAE	-0.820
FANCD2	-0.854
TPM2	-0.882
CBX3	-0.872
CUEDC2	-0.866
WEE1	-0.655
MKI67	-0.645
CALM1	-0.661
STUB1	-0.800
ANAPC10	-0.852
PAK5	-0.611
SGCB	-0.949
MDK	0.642
PSEN1	0.713
RELA	0.913
NCK1	1.001
PRKCI	1.121
PLCB4	-1.252
PPM1B	-1.784
ITGB1	-0.784
YWHAQ	-0.818
VIM	-0.711
MYL9	-1.319
HK1	-1.235
PRPF19	-0.727
GNAI3	-0.920
MDH1	-0.802
RHOA	-0.735
NDUFB6	-0.757
DBN1	-0.723
CDKN2A	-0.684
MAPK8	-0.764
GSR	-0.804
ATP6AP2	-0.992
YWHAZ	-0.870
CFL1	-1.223
CDC42	-1.168
CLDN11	-1.048
CDH2	-1.072
DKK3	-0.829
COL1A1	-1.304

ACTG2	-1.170
GLT8D1	-0.939
SNAP23	-0.862
PDLIM7	-1.135
RAB8A	-1.256
PARD3	-1.348
GCLM	0.842

Table S3. DEGs of XYS composed 8 herbs vs. DMSO treatment.

Gene_Symbol	BR	ASR	PRA	AMR	*Poria*	ZRR	MHH	GRR
PANX1	0.000	0.000	0.000	0.000	0.000	1.257	0.000	0.000
PTDSS2	0.000	2.740	0.000	0.000	0.000	0.000	0.000	0.000
ADCY1	0.000	0.000	0.000	3.785	0.000	0.000	0.000	0.000
MRC1	2.887	0.000	0.000	2.248	0.000	3.565	0.000	2.394
STAT3	0.000	0.000	0.702	0.000	0.000	0.000	0.000	0.000
OLIG2	0.000	0.000	0.000	0.000	0.000	6.341	0.000	0.000
PLA2G1B	0.000	5.135	0.000	5.087	0.000	0.000	0.000	0.000
ANAPC13	1.722	0.000	0.000	0.000	0.000	0.000	0.000	0.000
TFRC	0.000	0.000	0.720	0.000	0.000	0.000	0.000	0.000
CCNE1	0.000	0.000	0.000	0.000	0.000	2.618	0.000	0.000
CDK5R1	0.000	0.000	0.000	0.000	0.000	1.126	0.000	0.000
ACOT9	-1.656	0.000	0.000	0.000	0.000	0.000	0.000	0.000
PLD3	0.000	0.000	0.000	2.221	0.000	1.898	0.000	2.438
YY1	0.000	0.000	0.000	0.000	0.000	-3.763	0.000	0.000
YES1	0.000	0.000	0.000	0.000	0.000	-3.110	0.000	0.000
TNFSF8	0.000	3.904	0.000	0.000	0.000	0.000	0.000	3.994
CAPN2	0.000	0.000	-1.161	0.000	0.000	0.000	0.000	0.000
TSC22D3	2.238	0.000	0.000	0.981	0.000	0.000	0.000	0.000
PARD3	0.000	0.000	0.000	0.000	0.000	-2.268	0.000	0.000
MIS12	0.000	0.000	0.000	0.000	0.000	0.000	0.688	0.000
HMGCS1	5.293	0.000	0.000	0.000	0.000	0.000	0.000	0.000
KCNJ8	0.000	3.804	0.000	3.012	0.000	0.000	0.000	2.726
GRIK3	0.000	0.000	0.000	0.000	0.000	5.555	0.000	0.000
KAL1	6.076	0.000	0.000	2.721	0.000	0.000	-4.199	2.103
PRKCSH	-1.792	0.000	0.000	0.000	0.000	0.864	0.000	0.000
DRAM1	-4.184	0.000	0.000	0.000	0.000	-4.443	0.000	0.000
NFAT5	0.000	1.228	1.147	1.339	1.301	0.000	-1.289	1.313
PPP2R5A	-0.990	0.000	0.000	0.000	0.000	0.000	0.000	0.000
LSP1	0.000	0.000	0.000	2.716	0.000	5.752	0.000	0.000
ARHGEF11	0.000	0.000	0.000	1.801	0.000	3.334	0.000	1.849
FZD6	1.926	0.000	0.000	0.000	0.000	1.899	0.000	0.000
PSD3	2.423	0.000	0.000	0.000	0.000	0.000	0.000	0.000
CXCL6	0.000	0.000	0.000	0.000	0.000	2.095	0.000	0.000

FLT4	2.269	0.000	0.000	2.683	0.000	0.000	0.000	1.463
CCL18	0.000	0.000	0.000	0.000	0.000	7.666	0.000	0.000
RAB8A	0.000	0.000	0.000	0.000	0.000	-2.281	0.000	0.000
LTBP2	4.390	1.845	0.000	0.000	0.000	0.000	0.000	0.000
PGAM1	0.000	0.000	0.000	0.000	0.000	2.728	0.000	0.000
RAPGEF1	0.000	0.000	0.000	0.000	0.000	4.065	0.000	0.000
STAR	3.610	0.000	0.000	0.000	0.000	0.000	0.000	0.000
DNM1L	0.000	0.000	0.000	0.000	0.000	-2.143	0.000	0.000
ADCY7	0.000	0.000	0.000	-3.545	-4.558	0.000	0.000	0.000
SUCLG2	0.000	0.000	0.000	0.000	0.000	4.102	-1.716	0.000
PNN	-0.909	0.000	0.000	0.000	0.000	-1.264	0.000	0.000
RAB14	0.000	0.000	0.000	0.000	-3.441	0.000	0.000	0.000
ATP6V1G2	0.000	0.000	0.000	0.000	0.000	-4.315	0.000	0.000
DSP	0.000	0.000	0.000	0.000	0.000	4.275	0.000	0.000
RAP1A	0.000	0.000	0.866	1.393	0.000	1.855	0.000	1.152
BMPR2	0.000	0.000	0.000	0.000	-2.400	0.000	0.000	0.000
ACOX2	0.000	0.000	0.000	2.194	0.000	0.000	0.000	0.000
GSK3A	-1.136	0.000	0.000	0.000	0.000	0.000	0.000	0.000
CTSC	0.802	0.000	0.000	0.000	0.000	0.854	0.000	0.000
PARD6B	0.000	4.847	0.000	3.151	4.344	4.777	0.000	0.000
CAB39L	0.000	0.000	1.649	2.352	0.000	3.234	0.000	1.742
HSPA6	0.000	0.000	0.000	0.000	0.000	4.874	0.000	3.161
SCAND1	2.771	0.000	0.000	0.000	0.000	0.000	0.000	0.000
ATP2B4	5.821	0.000	0.000	0.000	0.000	0.000	0.000	0.000
LNPEP	2.582	0.000	0.000	0.000	0.000	0.000	0.000	0.000
CAPN1	4.047	0.000	0.000	0.000	0.000	0.000	0.000	0.000
IGFBP4	0.000	0.000	0.000	0.000	0.000	0.000	-1.661	2.279
TNFSF4	0.000	0.000	0.000	0.000	0.000	0.000	-0.654	0.000
NBL1	0.000	0.000	0.000	0.995	0.000	0.000	0.000	0.000
YAP1	-1.338	0.000	0.000	0.000	0.000	0.000	0.000	0.000
MDH1	0.000	0.000	0.000	0.000	0.000	-0.803	0.000	0.000
RASA2	0.000	0.000	-0.841	0.000	0.000	0.000	0.000	0.000
PRPS1L1	-1.619	0.000	0.000	0.000	0.000	0.000	0.000	0.000
PDK4	4.131	0.000	2.086	3.220	0.000	4.843	0.000	0.000
FGF2	0.759	0.000	0.000	0.000	0.000	0.000	0.000	0.000
CHD4	-2.978	0.000	0.000	0.000	0.000	-2.265	0.000	0.000
MTTP	0.000	2.864	0.000	3.442	0.000	0.000	0.000	0.000
DHX58	0.000	0.000	0.000	0.000	0.000	5.157	0.000	0.000
WNT16	0.000	0.000	0.000	0.000	0.000	0.000	0.000	3.946
DEGS1	0.000	0.000	0.000	0.000	0.000	-1.040	0.000	0.000
ADAMTS1	2.436	1.185	1.429	3.087	1.397	3.647	0.000	2.369
CD83	0.000	0.000	0.000	0.000	-2.943	0.000	0.000	0.000
PPP2R1B	0.000	0.000	-0.754	-0.965	-0.765	0.000	0.000	0.000

GBE1	0.000	0.000	0.000	0.000	0.000	-4.390	0.000	0.000
PSENEN	0.000	0.000	0.000	0.000	0.000	1.223	0.000	0.000
EOMES	0.000	0.000	0.000	3.274	0.000	5.382	0.000	0.000
PDP2	0.000	0.000	0.000	0.000	-4.609	0.000	0.000	0.000
APOA2	0.000	0.000	0.000	2.731	0.000	0.000	0.000	0.000
MYCN	0.000	3.628	0.000	0.000	0.000	0.000	0.000	0.000
MAP3K14	-2.171	0.000	0.000	0.000	0.000	-2.811	0.000	-0.638
OSBPL1A	0.000	0.000	0.000	0.000	0.000	0.000	0.868	0.000
HOMER1	2.416	0.000	0.000	1.105	0.000	1.456	0.000	0.588
SUCLG1	-1.478	0.000	0.000	0.000	0.000	0.000	0.000	0.000
PRG2	7.486	0.000	0.000	0.000	0.000	0.000	0.000	0.000
ANAPC7	0.000	0.000	0.000	-0.692	0.000	0.000	0.000	0.000
PPP2R4	0.000	-1.087	0.000	0.000	0.000	-4.422	0.000	0.000
TPCN1	0.000	0.000	0.000	0.000	0.000	2.818	0.000	0.000
GUCY1B1	0.000	0.000	0.839	0.000	-0.940	0.000	0.000	0.000
LDLR	-4.421	0.000	0.000	0.000	0.000	0.000	0.000	0.000
SPHK2	0.000	0.000	0.000	0.000	0.000	0.000	0.000	3.470
ITGA2	0.000	0.000	0.000	0.000	0.000	0.000	1.621	1.444
MID1	0.000	0.000	0.000	-0.802	0.000	-5.076	0.000	0.000
OGDHL	3.355	1.780	1.464	3.103	2.517	3.300	-1.657	3.905
CHAC1	0.000	0.000	0.000	0.000	0.000	5.709	0.000	0.000
SPTAN1	0.000	0.000	0.000	0.000	0.000	-4.179	0.000	0.000
MTFP1	0.000	0.000	0.000	2.427	0.000	3.487	0.000	0.000
CHIA	4.348	1.991	0.000	3.381	3.096	3.312	-2.991	1.812
PDHA1	0.000	0.000	0.000	-0.611	0.000	-1.263	0.000	0.000
MDM2	0.000	0.000	0.000	2.497	0.000	0.000	0.000	1.629
IFNA2	4.744	2.820	0.000	0.000	0.000	0.000	0.000	0.000
RBX1	-0.632	0.000	0.000	0.000	0.000	0.000	0.000	0.000
SOX4	0.000	0.000	0.000	1.309	0.000	3.409	0.000	0.000
AADAT	0.000	0.000	0.000	0.000	0.000	0.000	-3.908	0.000
MAGI1	1.325	0.000	0.000	0.000	0.000	0.000	0.000	0.000
STUB1	-0.604	0.000	0.000	0.000	0.000	0.000	0.000	0.000
HSPA8	0.000	0.000	-0.975	0.000	0.000	1.800	0.000	0.000
LLGL1	0.000	0.000	0.000	0.000	0.000	-5.469	0.000	0.000
GPS1	-1.085	0.000	0.000	0.000	0.000	-0.775	0.000	0.000
EFNB1	0.000	0.000	0.000	1.802	0.000	0.000	0.000	0.000
SLC7A5	0.000	3.139	0.000	3.566	0.000	0.000	0.000	0.000
EGR1	0.000	0.000	0.000	0.000	0.000	2.765	0.819	0.000
SUFU	0.000	0.000	0.000	1.130	0.899	0.000	-0.922	1.007
PPP3CC	4.936	0.000	0.000	0.000	0.000	0.000	0.000	0.000
STAM	0.995	0.000	0.000	0.000	0.000	0.000	0.000	0.000
STK4	0.000	0.000	0.000	0.000	0.000	-4.247	0.000	0.000
POLD3	0.000	0.000	0.000	0.692	0.000	0.000	0.000	0.000

BTNL10	0.000	2.873	0.000	2.013	0.000	0.000	0.000	0.000
CUL5	0.982	0.000	0.661	1.095	0.000	0.000	0.000	0.000
ITGA5	1.114	0.798	0.000	0.647	0.000	0.000	0.000	0.000
GAS2L3	0.000	0.000	0.873	0.000	0.000	0.000	0.000	0.000
MAPKAPK2	0.000	1.798	0.000	0.000	0.939	1.812	0.000	0.000
HAS2	0.000	0.000	0.000	2.645	0.000	0.000	0.000	2.358
USP5	0.000	0.000	0.000	1.261	0.000	0.000	0.000	0.000
ACO2	0.000	0.000	0.000	-0.694	0.000	1.437	0.000	0.000
HIF1A	-2.528	0.000	-0.703	-0.605	0.000	0.000	0.000	0.000
POFUT1	-4.157	0.000	0.807	0.000	0.000	0.000	0.000	0.000
FGF17	0.000	2.756	0.000	0.000	0.000	0.000	0.000	0.000
CASC3	0.000	0.000	0.000	0.000	0.000	-1.214	0.000	0.000
GALNT3	3.448	1.227	1.055	2.719	1.701	3.770	-1.320	2.960
NPBWR2	0.000	2.683	0.000	2.849	3.003	3.899	0.000	4.365
TOP2A	0.000	0.000	0.000	0.000	-0.765	0.000	0.000	0.000
MGRN1	0.000	0.000	0.000	0.000	0.000	-5.841	0.000	0.000
CASK	0.000	0.000	0.000	0.000	0.000	-5.166	0.000	0.000
BSG	0.000	0.000	0.000	0.000	0.000	-5.207	0.000	0.000
CUL7	0.000	0.000	0.000	-3.310	0.000	0.000	0.000	0.000
FLNC	0.000	0.000	-0.642	0.000	0.000	0.000	0.000	0.000
SHC1	0.000	0.000	-0.878	-1.287	0.000	2.053	-0.778	0.000
GNG12	-1.394	0.000	0.000	0.000	0.000	0.000	0.000	0.000
ANG	0.000	-2.502	0.000	0.000	0.000	0.000	0.000	0.000
CCNG1	0.000	0.000	0.733	0.000	0.000	0.896	0.000	0.000
WWTR1	0.000	0.000	0.000	0.000	0.000	1.312	0.000	0.000
PRKCI	0.000	0.000	0.741	0.000	0.000	0.000	0.000	0.000
PPP1CC	0.000	0.000	0.000	0.000	0.000	-1.194	0.000	0.000
HSD11B1	0.000	4.913	0.000	0.000	0.000	0.000	0.000	0.000
FABP5	0.000	3.624	0.000	0.000	0.000	0.000	0.000	0.000
HUS1	0.000	0.000	0.000	1.592	0.000	0.000	0.000	0.000
CLDN15	0.000	2.157	0.000	2.433	0.000	0.000	0.000	0.000
AIMP2	0.000	0.000	0.000	0.000	-1.026	0.000	0.000	0.000
PFKFB3	0.000	0.000	0.000	0.000	-5.323	0.000	0.000	0.000
FUT8	-1.298	0.000	0.000	0.000	0.000	0.000	0.000	0.000
LARS	2.950	0.000	0.000	0.000	0.000	0.000	0.000	0.000
ZC3H7A	0.000	0.000	0.000	0.000	0.000	-1.615	0.000	0.000
MBD1	-4.362	0.000	0.000	0.000	0.000	0.000	0.000	0.000
PLEC	0.000	0.000	-0.974	0.000	0.000	0.000	0.000	0.000
CLTCL1	0.000	0.000	-0.587	0.000	0.000	-5.676	0.000	0.000
FGF5	0.000	0.000	0.000	0.000	0.000	-1.031	0.000	0.000
AKR1B1	0.000	0.000	0.000	0.000	0.000	0.000	0.728	0.000
TCF4	0.000	0.000	0.000	0.000	0.000	-4.888	0.000	-1.141
TNFRSF14	0.000	2.034	0.000	0.000	2.770	0.000	0.000	0.000

1-Mar	0.000	2.145	0.000	2.114	0.000	4.962	0.000	3.111
CASP2	0.000	0.000	0.000	0.000	0.000	-4.572	0.000	0.000
GNRH2	0.000	2.783	0.000	0.000	2.066	2.619	0.000	0.000
MET	0.000	0.000	0.000	0.000	-0.752	0.000	0.000	0.000
MLN	0.000	4.579	0.000	0.000	0.000	0.000	0.000	0.000
FOS	0.000	0.000	1.776	0.000	0.000	4.229	0.000	0.000
IL17D	0.000	0.000	0.000	2.316	2.877	4.631	0.000	0.000
LIMK1	0.000	0.000	0.000	0.000	0.000	-3.042	0.000	0.000
PAK3	0.000	1.581	0.000	0.000	0.000	0.000	-3.382	0.000
EXOSC8	0.000	0.000	0.000	0.000	0.000	1.418	0.000	0.000
ATP6V0E1	0.000	0.000	0.000	0.000	0.000	0.698	0.000	0.000
RNF7	0.000	0.000	0.000	0.000	0.000	0.993	0.000	0.000
FXYD4	0.000	0.000	0.000	0.000	0.000	0.000	0.000	3.347
GLUL	0.000	0.000	0.000	-0.689	0.000	0.000	0.000	0.000
VEGFC	0.000	0.000	-1.027	0.748	0.000	0.000	0.000	0.000
ADAM17	0.000	-1.311	0.000	0.000	0.000	0.000	0.000	0.000
DLL1	0.000	0.000	0.000	0.000	0.000	0.000	0.000	-3.585
DKK1	0.000	-0.634	0.000	0.000	0.000	-2.293	-0.728	0.000
CSDE1	-1.938	0.000	0.000	0.000	0.000	0.000	0.000	0.000
E2F5	0.000	0.000	0.000	2.708	0.000	0.000	0.000	0.000
SERPIND1	0.000	0.000	0.000	2.274	0.000	0.000	0.000	0.000
C6	0.000	0.000	0.000	3.302	0.000	3.530	0.000	0.000
IL7R	0.000	-0.683	0.000	0.000	0.000	-3.210	0.000	-0.924
BIRC5	-5.366	0.000	0.000	0.000	0.000	0.000	0.000	0.000
HNRNPA1	0.000	0.000	0.000	0.000	0.000	0.629	0.000	0.000
SLC9A1	0.000	0.000	0.000	0.000	0.000	-4.681	0.000	0.000
EEA1	1.585	0.000	0.000	0.907	0.000	1.846	0.000	1.220
USP54	4.111	0.000	0.000	0.000	0.000	0.000	0.000	0.000
RCHY1	0.000	0.000	1.290	2.258	0.000	2.637	0.000	2.332
NPY5R	0.000	0.000	0.000	4.107	0.000	0.000	0.000	0.000
DSG2	0.000	1.662	1.285	3.050	2.096	2.320	-2.147	3.336
ADI1	0.000	0.000	0.000	0.000	0.000	1.135	0.000	0.000
A2M	0.000	2.012	1.362	2.908	0.000	3.501	-1.997	3.723
LAMB3	0.000	0.000	-0.764	0.000	0.000	0.000	0.000	0.000
KITLG	0.000	0.000	0.764	0.000	0.000	0.000	0.000	0.722
FCER1A	0.000	0.000	0.000	2.718	0.000	3.400	0.000	3.253
IFNA10	3.624	1.544	0.000	2.162	3.419	0.000	-4.573	2.775
ACAD9	0.000	0.000	0.000	0.000	0.000	0.000	0.000	0.888
MAP1LC3B	0.000	0.000	0.000	0.000	0.000	1.076	0.000	0.000
SCTR	0.000	0.000	0.000	4.164	0.000	0.000	0.000	0.000
CCR6	0.000	2.965	0.000	0.000	0.000	5.291	0.000	0.000
GHRH	0.000	0.000	0.000	3.148	0.000	0.000	0.000	0.000
DLX2	0.000	0.000	0.000	0.000	0.000	4.194	0.000	0.000

P2RY14	0.000	4.288	0.000	0.000	0.000	0.000	0.000	0.000
PPIL2	0.000	0.000	0.000	-1.199	0.000	0.000	0.000	0.000
F2RL3	0.000	0.000	0.000	0.000	0.000	7.666	0.000	4.191
IBSP	0.000	0.000	0.000	0.910	0.000	0.000	0.000	0.000
ROCK1	0.000	0.000	0.000	2.910	0.000	0.000	0.000	0.000
PINX1	0.000	0.000	0.000	0.000	0.000	0.000	-3.267	0.000
MEF2A	0.000	0.000	0.000	3.555	0.000	0.000	0.000	0.000
HOXA5	0.000	0.000	0.000	0.000	0.000	2.898	0.000	0.000
PDE5A	0.000	1.751	0.000	0.000	0.000	0.000	0.000	0.000
CTSH	0.000	0.000	0.000	0.000	0.000	-4.379	0.000	0.000
SERPINF1	7.327	0.000	0.000	0.000	0.000	0.000	0.000	0.000
VCL	0.000	0.000	0.000	0.000	0.000	0.000	-0.624	0.000
STAT6	5.403	0.000	0.000	3.610	0.000	0.000	0.000	2.917
UCP2	0.000	0.000	0.000	0.000	0.000	0.000	0.000	-1.141
ADIPOR1	-4.705	0.000	0.000	0.000	0.000	0.000	0.000	0.000
RRAGA	-5.210	0.000	0.000	0.000	0.000	0.000	0.000	0.000
NR4A1	0.000	2.597	0.000	0.000	0.000	0.000	0.000	0.000
MAPK3	1.689	0.000	0.000	0.000	0.000	0.000	0.000	0.000
MYL9	0.000	0.000	0.000	0.000	0.000	-0.764	0.000	0.000
KNTC1	2.177	0.000	0.000	0.000	0.000	0.000	0.000	0.000
HK1	0.000	0.000	0.000	0.000	0.000	-0.634	0.000	0.000
KRT5	0.000	0.000	0.000	2.890	0.000	0.000	0.000	3.805
CLK1	-1.388	0.000	0.000	0.000	0.000	1.413	0.000	0.000
ACADVL	-4.716	0.000	0.000	0.000	0.000	0.000	0.000	0.000
RHOBTB2	0.000	1.311	0.000	1.152	0.000	0.000	0.000	0.000
TIAM1	0.000	0.000	0.000	3.687	0.000	0.000	0.000	0.000
CIR1	1.263	0.000	0.000	0.000	0.000	0.000	0.000	0.000
EZR	0.860	0.000	0.000	0.000	0.000	0.000	0.000	0.000
MAP3K8	4.406	0.000	0.000	0.000	0.000	3.602	0.000	0.000
PSME2	0.000	0.000	0.000	0.000	0.000	-1.317	0.000	0.000
ATP5A1	0.000	0.000	0.000	0.000	0.000	-2.259	0.000	0.000
GNAI3	0.000	0.000	0.000	0.000	0.000	-0.746	0.000	0.000
TNFSF11	2.729	1.329	2.226	3.425	1.428	3.099	0.000	2.320
LHB	0.000	0.000	0.000	3.666	0.000	0.000	0.000	0.000
TAOK3	0.000	0.000	0.000	0.000	0.000	0.000	0.000	0.887
ETV4	0.000	0.000	0.000	0.000	0.000	1.462	0.000	0.000
TBC1D7	1.426	0.000	0.000	0.000	0.000	0.000	0.000	0.000
AFF1	0.000	0.000	0.000	1.148	0.000	0.000	0.000	0.000
MST1R	0.000	0.000	0.000	3.524	0.000	0.000	0.000	0.000
COL1A1	0.000	0.000	0.000	-0.622	0.000	-1.161	0.000	-0.753
PAG1	3.066	2.140	0.000	2.598	2.246	3.151	-2.600	2.719
MGAT5	1.497	0.000	0.000	0.000	0.000	-5.257	0.000	0.000
NIT2	-4.511	0.000	0.000	0.000	0.000	0.000	0.000	0.000

CLIC4	1.834	0.000	0.000	1.143	0.000	0.000	0.000	0.736
HNRNPA0	0.000	-0.654	0.000	0.000	0.000	0.000	0.000	0.000
COX2	0.746	0.000	0.000	0.000	0.000	-1.144	0.000	0.000
WNT3A	0.000	0.000	0.000	0.000	0.000	0.000	0.000	3.825
XRCC1	2.678	0.000	1.386	1.753	0.000	0.000	0.000	0.000
CDC26	0.000	0.000	0.000	0.000	0.000	1.339	0.000	0.000
DDIT3	1.801	0.000	0.803	0.000	0.000	2.075	0.000	0.000
ANAPC1	0.000	0.000	-1.086	0.000	0.000	0.000	0.000	0.000
PRTN3	7.071	0.000	0.000	0.000	0.000	0.000	0.000	0.000
SEPW1	0.000	0.000	-1.480	0.000	0.000	0.000	0.000	0.000
CRH	4.426	0.000	0.000	0.000	0.000	0.000	0.000	0.000
RYK	0.000	-3.001	-2.640	0.000	-4.404	0.000	0.000	0.000
LRP6	0.000	0.000	0.000	0.000	0.000	3.106	0.000	0.000
KCNMB3	4.992	0.000	3.660	4.351	0.000	0.000	0.000	0.000
IDH1	0.000	0.000	0.000	0.000	0.000	0.000	0.000	-2.102
PPAT	1.228	0.993	0.888	2.257	0.000	2.341	0.000	2.116
BCAR1	0.000	0.000	0.000	0.000	0.922	0.000	0.000	0.000
PRDX1	0.000	0.000	-1.086	0.000	0.000	0.000	0.000	0.000
TSHZ3	0.000	0.000	-3.312	0.000	0.000	0.000	0.000	0.000
ATP6V1H	0.000	0.000	0.000	2.053	0.000	2.275	0.000	0.000
DVL2	-4.372	0.000	0.000	0.000	0.000	0.000	0.000	0.000
CDK2	0.000	0.000	0.000	0.944	0.000	1.262	0.000	0.000
ATF2	0.000	0.000	0.807	0.000	0.000	0.000	0.000	0.000
CLPS	0.000	0.000	0.000	0.000	0.000	7.168	0.000	0.000
VCAM1	3.947	0.000	0.000	3.330	0.000	0.000	0.000	2.080
DCTN1	1.024	0.000	0.000	0.000	0.000	0.000	0.000	0.000
TFF3	0.000	3.121	0.000	0.000	0.000	0.000	0.000	0.000
MYL6B	0.000	0.000	0.000	0.000	0.000	1.061	0.000	0.000
TXNIP	0.000	0.000	1.303	1.697	0.000	0.000	0.000	0.000
GNG5	-0.654	0.000	0.000	0.000	0.000	0.000	0.000	0.000
ATP5G2	0.000	0.000	0.000	0.000	-0.879	0.000	-0.725	0.000
PRKAA1	-0.891	0.000	0.000	0.000	0.000	0.000	0.000	0.000
LEPR	0.000	0.000	0.000	2.028	2.887	0.000	0.000	2.414
CD40LG	3.559	0.000	1.693	2.613	0.000	3.179	-3.014	2.732
CASP1	4.201	0.000	0.000	0.000	0.000	0.000	0.000	0.000
CFL1	0.000	0.000	0.000	0.000	0.000	-1.363	0.000	0.000
CLDN12	0.000	1.029	0.000	0.000	0.000	2.053	0.000	0.770
LPAR1	0.000	0.000	0.000	0.000	0.000	0.000	-1.093	0.000
PLA2G2A	7.486	0.000	0.000	0.000	0.000	0.000	0.000	4.595
SET	0.000	0.000	0.000	0.000	-0.841	1.093	0.000	0.000
ACSL4	0.000	0.000	0.000	0.971	0.000	0.882	0.000	0.000
CNGA4	3.711	0.000	0.000	2.177	0.000	0.000	0.000	2.107
IRF5	0.000	0.000	0.000	0.000	0.000	0.000	0.000	3.081

LIG1	1.855	0.000	0.000	0.000	0.000	0.000	0.000	0.000
NFKB1	0.000	0.000	0.742	0.000	0.000	-4.360	0.000	0.000
CDON	0.000	0.000	0.000	0.000	0.000	2.575	0.000	0.000
TAAR8	0.000	0.000	0.000	4.022	0.000	4.923	0.000	4.162
GPR35	0.000	0.000	0.000	1.605	1.344	2.580	0.000	1.643
PYDC1	0.000	0.000	0.000	2.875	2.923	0.000	-4.468	3.506
ALDH18A1	2.544	1.782	1.266	2.881	2.771	2.923	-1.689	4.158
DTX1	0.000	0.000	0.000	2.822	0.000	0.000	0.000	1.588
GAMT	0.000	0.000	0.000	0.000	0.000	0.000	-1.742	0.000
KCNK9	3.148	0.000	0.000	2.330	0.000	0.000	0.000	0.000
SUCLA2	0.000	-0.813	0.000	0.000	0.000	-2.985	0.000	0.000
HTR2C	0.000	0.000	0.000	4.204	0.000	0.000	0.000	0.000
RB1	0.000	0.000	0.000	0.000	-1.326	0.000	0.000	0.000
LTK	6.163	0.000	0.000	0.000	0.000	0.000	0.000	0.000
GADD45A	0.000	0.719	0.000	0.000	0.000	0.000	0.000	0.000
PGLS	0.000	0.000	0.000	2.185	1.539	0.000	-1.930	0.000
CLDN19	0.000	0.000	0.000	0.000	0.000	5.396	0.000	0.000
PTAFR	0.000	0.000	0.000	3.258	0.000	0.000	0.000	0.000
NDUFC2-KCTD14	0.000	2.048	2.132	3.175	0.000	0.000	-1.784	0.000
ATG4B	1.759	0.000	0.000	0.000	0.000	1.792	0.000	0.000
C8A	0.000	2.499	0.000	2.677	0.000	0.000	0.000	2.850
DDX58	0.000	0.000	0.000	0.000	-3.372	0.000	0.000	0.000
LATS1	-4.177	0.000	0.000	0.000	0.000	1.451	0.000	0.000
MT2A	0.000	0.000	-1.524	0.000	0.000	0.000	0.000	0.000
VAV2	0.000	0.000	0.000	0.000	0.000	0.000	0.000	1.995
C1D	-1.022	0.000	0.000	0.000	0.000	0.000	0.000	0.000
ATP6V0A1	0.000	0.833	0.000	0.000	0.000	0.000	0.000	0.000
FAS	0.000	2.910	0.000	2.801	0.000	0.000	0.000	1.404
PPRC1	1.824	0.000	0.000	1.036	0.000	0.000	0.000	-1.197
FJX1	0.000	0.000	-0.768	0.000	0.000	0.000	0.000	0.000
PRKCA	-4.550	0.000	0.000	0.000	0.000	0.000	0.000	0.000
PTPN1	-1.104	0.000	0.000	0.000	0.000	-1.215	0.000	0.000
HPRT1	0.648	0.000	0.000	0.000	0.000	-0.859	0.000	0.000
VEZT	0.000	0.000	0.759	0.000	0.000	0.000	0.000	0.000
BCL2L11	0.000	0.000	0.758	0.000	-0.861	0.000	-0.784	0.000
TIMP4	1.670	0.000	0.000	1.156	0.000	0.000	0.000	0.000
PRKCB	4.808	3.059	0.000	3.144	0.000	0.000	0.000	0.000
TUBA8	0.000	0.000	1.996	3.474	1.792	3.453	-3.902	3.517
NLN	-2.967	0.000	0.000	0.000	0.000	0.000	0.000	0.000
HOXC13	0.000	0.000	0.000	0.000	0.000	3.428	0.000	0.000
ZNF281	3.448	0.000	1.809	3.207	0.000	3.896	0.000	1.871
CD34	0.000	2.974	0.000	0.000	0.000	0.000	0.000	0.000

ANXA1	2.014	0.588	0.000	2.066	0.000	2.570	0.000	1.982
HCAR2	0.000	0.000	0.000	2.076	0.000	3.221	-3.499	0.000
IL20RB	4.243	2.669	2.042	3.532	0.000	3.426	-4.062	0.000
KCNMB4	1.254	0.000	0.000	-1.163	-0.684	0.000	0.000	0.000
HERC3	0.000	-0.868	0.000	0.000	0.000	-4.639	0.000	0.000
PPA1	-1.222	0.000	0.000	0.000	0.000	-0.996	0.000	0.000
MAPK10	0.000	0.000	1.657	2.927	0.000	0.000	0.000	2.085
AKT1S1	0.000	-0.748	0.000	0.000	-0.685	0.000	0.000	0.000
PIP5K1A	0.000	0.000	0.000	0.000	0.000	2.117	0.000	0.000
TOP1	0.000	0.000	0.744	0.000	0.000	0.000	0.000	0.000
IFNA16	0.000	0.000	0.000	2.363	3.591	0.000	0.000	0.000
NDUFAB1	0.000	0.000	-1.719	0.000	0.000	0.000	0.000	0.000
TNFRSF8	0.000	0.000	0.000	0.000	0.000	7.720	0.000	0.000
F2RL2	0.000	4.977	0.000	0.000	0.000	0.000	0.000	0.000
CALM1	-0.785	0.000	0.000	0.000	0.000	0.000	0.000	0.000
CST	3.116	0.000	0.000	2.490	0.000	3.819	0.000	3.772
IFNGR1	0.000	0.000	1.622	1.648	0.000	3.834	0.000	0.000
EIF2S1	0.000	0.000	0.000	0.000	0.000	-0.722	0.000	0.000
ORAI1	0.000	0.000	0.000	0.000	0.000	0.000	0.000	1.223
TNFRSF9	0.000	0.000	0.000	0.000	0.000	2.296	0.000	0.000
MT1A	1.813	0.000	-0.905	0.000	0.000	0.000	0.000	0.000
APOBEC3G	0.000	0.000	3.512	0.000	0.000	0.000	0.000	0.000
GTF2I	-2.104	0.000	0.000	0.000	0.000	0.000	0.000	0.000
COL4A1	0.000	0.000	0.000	0.000	0.000	0.000	-0.826	0.000
PANX2	0.000	0.000	0.000	0.000	0.000	0.000	0.936	0.000
MAP4K2	3.746	1.438	1.640	3.314	1.572	3.364	-1.395	3.744
AGER	3.221	1.929	0.000	1.678	1.199	0.000	0.000	0.000
RAB5C	-6.550	0.000	0.000	0.000	0.000	0.000	0.000	0.000
DUSP5	-5.003	0.000	0.000	0.000	0.000	0.000	0.000	0.978
GNG10	0.000	0.000	0.000	0.000	0.000	-3.296	0.000	0.000
KRAS	1.165	0.000	0.000	0.000	0.000	0.000	0.000	0.000
STIM2	0.000	1.008	0.000	0.000	0.000	0.000	0.000	0.000
IFNA14	3.912	0.000	0.000	2.547	0.000	4.408	0.000	0.000
CDC42EP3	0.000	0.000	-0.695	-1.012	0.000	0.000	0.000	0.000
ITCH	0.000	0.000	0.000	0.000	-1.113	0.000	0.000	0.000
GHSR	0.000	0.000	1.296	2.676	0.000	4.150	-2.290	3.869
CTSA	0.000	0.000	0.000	0.000	0.000	1.124	0.000	0.000
RELN	0.000	0.000	0.000	4.845	0.000	0.000	0.000	0.000
B3GALTL	-4.304	0.000	0.000	0.000	-0.790	0.000	0.000	0.000
TNFSF15	0.000	0.000	0.000	0.000	0.000	0.000	0.000	2.496
ARHGDIA	-2.310	0.000	0.000	0.000	0.000	0.000	0.000	0.000
RHOQ	0.000	0.000	0.000	0.000	0.000	4.571	0.000	0.000
DLC1	0.000	0.000	0.000	0.000	0.000	-1.237	0.000	0.000

ITPR3	0.000	2.735	0.000	3.981	0.000	0.000	0.000	0.000
PSME1	2.350	0.831	1.125	2.430	0.911	3.202	-0.832	2.931
PVR	0.000	1.283	0.000	0.000	0.000	0.000	0.000	0.000
MC1R	0.000	2.815	0.000	0.000	0.000	0.000	0.000	0.000
ADCY6	0.000	-0.823	0.000	0.000	0.000	0.000	0.000	0.000
PRKD3	1.905	0.000	0.000	0.737	0.000	0.000	0.000	0.000
PYCARD	0.000	3.257	0.000	0.000	0.000	4.187	0.000	0.000
GLT8D1	-1.362	0.000	0.000	0.000	0.000	-0.816	0.000	0.000
CCL24	0.000	0.000	0.000	2.099	0.000	3.913	0.000	3.993
BIRC7	0.000	3.616	0.000	3.696	0.000	0.000	0.000	3.068
SLC22A5	-4.260	0.000	0.000	0.000	0.000	0.000	0.000	0.000
ADRA1A	0.000	0.000	0.000	2.044	2.848	0.000	0.000	3.242
HRH2	0.000	0.000	0.000	0.000	0.000	6.135	0.000	0.000
IVD	0.000	0.000	0.000	0.000	-1.271	0.000	0.000	0.692
ACAT1	2.277	0.000	0.000	0.000	0.000	0.000	0.000	0.000
AIMP1	1.553	0.000	0.000	0.000	0.000	0.000	0.000	0.000
IHH	0.000	4.073	0.000	0.000	0.000	0.000	0.000	0.000
HTT	0.000	0.000	0.000	0.000	0.000	-4.252	0.000	0.000
FBXO2	4.026	1.591	0.000	2.859	2.890	3.403	-2.107	3.747
ALDH9A1	0.000	1.053	0.000	0.000	0.000	0.000	0.000	0.000
PLK3	0.000	0.000	0.000	0.000	-4.545	0.000	0.000	0.000
HERC4	0.000	0.000	0.000	0.000	0.000	0.000	0.000	-1.069
AP2A2	0.000	0.000	0.000	0.000	0.000	0.000	0.000	0.722
VIM	0.000	0.000	-0.847	0.000	0.000	-0.751	0.000	0.000
CR1	7.590	0.000	0.000	0.000	0.000	0.000	0.000	0.000
GTF2IRD1	0.000	0.000	0.000	1.910	0.000	0.000	0.000	0.000
EDNRB	0.000	4.282	0.000	3.700	0.000	0.000	0.000	0.000
CLEC6A	8.486	0.000	0.000	0.000	0.000	0.000	0.000	0.000
YWHAQ	0.000	0.000	-0.701	0.000	0.000	-1.034	0.000	0.000
NDUFB10	3.337	0.000	0.000	0.000	0.000	3.248	0.000	0.000
BBC3	0.000	0.000	0.000	0.000	0.000	1.770	0.000	0.000
ENOPH1	0.000	0.000	0.000	0.000	0.000	-4.154	0.000	0.000
GCLC	0.000	0.000	0.898	0.000	0.000	0.000	0.000	0.000
ZYX	-2.849	0.000	0.000	0.000	0.000	-2.808	0.000	0.000
BCAT1	0.000	0.000	0.000	-0.731	0.000	0.000	0.000	0.000
SCN4B	0.000	2.061	0.000	2.052	-3.792	0.000	0.000	0.000
EHD3	0.000	0.000	0.000	0.000	0.000	2.435	0.000	0.000
HLA-DPB1	0.000	0.000	0.000	0.000	0.000	-4.126	0.000	0.000
HGF	0.000	0.000	0.000	2.722	0.000	0.000	0.000	0.000
CHEK1	0.000	0.000	0.000	0.000	0.000	-4.409	0.000	0.000
PFKM	0.000	1.768	0.000	2.290	2.443	0.000	-4.421	0.000
PLEK2	0.000	4.194	0.000	0.000	0.000	0.000	0.000	0.000
INSR	0.000	-2.837	0.000	0.000	0.000	0.000	0.000	0.000

P2RY2	0.000	0.000	0.000	4.428	0.000	0.000	0.000	0.000
NCEH1	0.000	-1.577	0.000	0.000	0.000	2.058	0.000	0.000
GCNT1	-2.302	-0.751	-0.940	-0.858	0.000	0.000	0.000	0.000
KAT2A	2.845	0.000	0.000	0.000	0.000	0.000	0.000	0.000
ARRB2	1.283	0.000	0.000	0.000	0.000	1.941	0.000	0.000
ATG10	0.000	0.000	0.000	2.646	0.000	0.000	0.000	0.000
EIF4A1	-0.855	0.000	-0.772	0.000	0.000	-0.795	0.000	0.000
TRIM32	0.000	1.186	1.909	3.530	1.728	2.898	-3.628	3.588
TGFBI	0.000	0.000	-0.841	0.000	0.000	0.000	0.000	0.000
MAP2K3	1.996	0.000	0.000	0.000	0.000	0.000	0.000	0.000
GOT1	0.000	0.000	0.000	0.000	0.000	0.000	0.000	-3.317
TUBB	0.828	0.000	0.000	0.000	0.000	0.000	0.000	0.000
CX3CL1	0.000	0.000	0.000	3.185	0.000	0.000	0.000	3.569
HADHA	0.000	-0.699	0.000	0.000	0.000	0.000	0.000	0.000
RPTOR	0.000	2.390	0.000	0.000	0.000	0.000	0.000	0.000
SOCS5	2.477	1.520	1.418	3.145	1.459	3.590	-1.701	2.547
CTNNBIP1	0.000	0.000	0.000	-0.692	0.000	0.000	0.000	0.000
MDH1B	3.358	0.000	0.000	1.855	0.000	3.339	0.000	0.000
TRAF2	0.000	0.000	0.000	0.000	0.000	5.020	0.000	0.000
TAOK2	0.000	0.000	1.157	2.076	1.391	2.810	0.000	2.867
CTGF	0.000	0.000	-0.625	0.000	0.000	0.000	-0.626	0.000
UBE2G2	-5.350	0.000	0.000	0.000	0.000	0.000	0.000	0.000
NMB	0.000	1.443	0.000	2.783	1.127	0.000	0.000	1.183
IFNE	0.000	0.000	-0.890	0.618	0.000	-4.694	0.000	0.778
CAMK1	0.000	1.957	1.775	0.000	0.000	3.662	0.000	0.000
GP1BB	0.000	-0.946	0.000	0.000	0.000	0.000	0.000	0.000
RHOB	1.300	0.000	-1.107	0.000	0.000	1.501	0.000	0.000
LOX	0.000	0.000	0.772	0.000	0.000	0.000	-0.650	0.000
CDC25C	1.750	0.000	0.621	0.000	0.000	-4.374	0.000	0.000
AK4	0.000	0.000	0.000	1.877	0.000	0.000	0.000	3.027
SNW1	0.000	0.000	0.000	2.410	0.000	4.013	0.000	0.000
MMP15	0.000	0.000	0.000	0.000	3.496	0.000	0.000	0.000
CCND3	0.000	0.000	0.000	0.000	0.000	1.409	0.000	0.000
TRPM7	1.345	0.000	0.000	0.000	0.000	1.621	0.000	0.000
F10	7.067	0.000	0.000	0.000	0.000	0.000	0.000	0.000
ING1	0.000	0.000	0.000	0.000	0.000	2.428	0.000	0.000
NFATC2	0.000	0.000	-3.434	0.000	0.000	0.000	0.000	1.855
DHCR7	0.000	0.000	0.000	0.000	0.000	-1.175	0.000	0.000
ITGA4	2.914	0.000	0.000	0.000	0.000	3.761	0.000	0.000
BAD	-1.905	0.000	0.000	0.000	0.000	0.000	0.000	0.000
CNGB3	0.000	3.717	0.000	0.000	3.294	0.000	0.000	0.000
DOLK	-1.137	0.000	0.000	0.000	0.000	0.000	0.000	0.000
CTSS	0.000	0.000	0.000	2.236	2.425	0.000	0.000	3.148

ITPKA	-4.314	0.000	0.000	0.000	0.000	-4.340	0.000	-0.729
SOCS1	0.000	0.000	0.000	0.000	0.000	4.185	0.000	0.000
DYNC2H1	0.000	0.000	-1.449	1.127	0.000	0.000	-1.331	0.789
FSHR	0.000	3.164	3.273	4.945	0.000	5.118	0.000	0.000
HRAS	0.000	0.000	0.000	0.000	0.000	0.000	0.000	-1.918
PDE4D	2.685	2.374	1.727	3.377	0.000	0.000	-2.371	2.436
GEMIN5	0.000	0.000	-3.515	0.000	0.000	0.000	0.000	0.000
PPP1R12A	-0.827	0.000	0.000	0.000	0.000	-1.525	0.000	0.000
PML	-3.528	0.000	0.000	0.000	0.000	0.000	0.000	0.000
SLC2A1	5.742	0.000	0.000	0.000	0.000	0.000	0.000	0.000
FOXP2	0.000	0.000	0.000	2.883	0.000	3.622	0.000	0.000
TEAD3	0.000	0.000	0.000	0.000	0.000	0.000	-2.359	-1.563
PLIN5	0.000	0.000	0.000	2.495	0.000	0.000	0.000	0.000
HCRT	0.000	2.264	0.000	0.000	5.049	0.000	0.000	0.000
IL23A	3.464	1.167	1.481	3.095	1.751	3.726	-1.837	3.081
CCNT1	0.000	0.000	0.803	0.000	0.000	-4.275	0.000	0.000
CAV2	0.000	0.000	1.110	1.833	0.000	3.074	0.000	1.971
ING4	0.000	0.000	0.000	0.000	0.000	2.833	0.000	0.000
GSTO2	4.836	0.000	0.000	3.983	0.000	0.000	0.000	0.000
SOX9	0.000	0.000	-1.469	0.000	0.000	2.168	0.000	0.000
DNAJB6	0.000	0.000	0.000	0.000	0.000	1.102	0.000	0.000
SCD5	1.339	0.000	0.000	0.000	0.000	0.000	0.000	0.000
TAB2	0.000	0.000	0.689	0.000	0.000	0.805	0.000	0.000
CTSF	2.914	0.000	0.000	0.000	0.000	0.000	0.000	0.000
EIF4E2	1.656	-0.958	0.000	0.000	-0.830	0.000	0.000	0.000
SELP	0.000	5.190	0.000	0.000	0.000	0.000	0.000	0.000
ITGB2	0.000	0.000	0.000	2.222	0.000	0.000	0.000	0.000
SLC8A3	8.656	0.000	0.000	0.000	0.000	0.000	0.000	0.000
AXL	0.000	0.000	0.000	0.000	0.000	-2.955	0.000	0.000
GPR119	3.406	2.477	0.000	2.428	2.651	3.139	-1.522	4.253
CSNK2B	0.000	0.000	-0.854	0.000	0.000	0.000	0.000	0.000
B4GALT3	2.770	0.000	0.000	0.000	-2.238	0.000	0.000	0.000
PPM1A	0.000	0.000	0.000	0.000	0.000	-5.233	0.000	0.000
TGM2	0.000	-2.798	-1.625	0.000	0.000	0.000	0.000	0.000
IRS1	0.000	-0.736	-1.050	0.000	0.000	1.184	-0.720	0.000
IL18R1	3.518	0.000	0.000	2.319	0.000	0.000	-3.115	2.965
GFPT2	0.000	1.586	0.000	1.409	0.000	0.000	0.000	0.000
PPM1B	-0.734	0.000	0.000	0.000	0.000	0.000	0.000	0.000
MAML3	3.355	1.015	1.245	2.784	2.222	3.066	-2.830	3.851
FN1	-1.276	0.000	0.000	0.000	0.000	-2.195	-0.730	0.000
GABRE	4.504	4.434	0.000	2.690	0.000	0.000	0.000	0.000
MSH2	0.000	0.000	0.000	0.000	-0.772	0.000	0.000	0.000
DIABLO	0.000	0.000	0.000	0.000	0.000	-4.310	0.000	0.000

LAMP1	2.807	0.000	0.000	0.000	0.000	0.000	0.000	0.000
GABRQ	0.000	0.000	-1.364	0.000	0.000	-4.761	0.000	0.000
CXCL11	0.000	0.000	0.000	0.000	0.000	7.753	0.000	0.000
PSMB9	0.000	0.000	0.000	0.000	2.233	0.000	0.000	0.000
MGAT2	-1.685	0.000	0.000	0.000	0.000	0.000	0.000	0.000
SGCB	-0.780	0.000	0.000	0.000	0.000	-0.752	0.000	0.000
MYBPH	0.000	0.000	0.000	3.401	0.000	4.028	0.000	0.000
PKP2	0.000	0.000	0.000	-1.564	0.000	0.000	-0.710	0.000
TGFB3	0.000	0.000	2.659	3.839	0.000	5.513	0.000	3.372
BMP5	7.245	0.000	0.000	0.000	0.000	0.000	0.000	0.000
CORT	0.000	2.861	0.000	2.991	0.000	5.995	0.000	0.000
CEBPD	0.000	0.000	0.000	-0.975	0.000	0.000	0.000	0.000
TLR2	0.000	5.278	0.000	0.000	0.000	6.547	0.000	0.000
HSD3B2	0.000	3.975	0.000	3.806	0.000	5.231	0.000	0.000
QPRT	0.000	0.000	0.000	2.455	0.000	4.464	-2.535	3.916
SP110	1.565	0.000	0.000	2.198	0.000	2.895	0.000	2.660
PLA2G4C	0.000	0.000	0.000	0.000	0.000	0.000	1.252	0.000
TUBA3E	0.000	0.000	0.000	4.923	0.000	0.000	0.000	0.000
PRSS3	0.000	0.000	0.000	2.988	0.000	7.100	0.000	0.000
ITGB1	-0.830	0.000	-0.892	0.000	0.000	-0.878	0.000	0.000
DAO	0.000	0.000	0.000	0.000	0.000	4.765	0.000	0.000
FOXO4	0.000	0.000	0.000	0.000	0.000	3.171	0.000	-2.475
CSNK1G3	2.148	0.000	1.563	3.021	2.567	4.204	-1.923	4.360
PSMG2	2.217	0.000	0.000	0.000	0.000	0.000	0.000	0.000
CAMK1D	4.499	1.844	1.655	3.022	1.663	2.999	0.000	3.154
ITLN1	0.000	3.713	0.000	0.000	0.000	0.000	0.000	0.000
TNNI2	0.000	0.000	0.000	0.000	0.000	6.666	0.000	0.000
RPS5	-2.599	0.000	0.000	0.000	0.000	0.000	0.000	0.000
HCRTR1	3.797	3.222	0.000	2.432	0.000	0.000	0.000	3.239
CUL4B	0.000	0.000	0.000	0.000	0.000	-1.583	0.000	-0.719
CHRNA9	0.000	0.000	0.000	0.000	0.000	3.234	0.000	0.000
EDEM3	-2.334	0.000	0.000	0.000	0.000	0.000	0.000	0.000
PTK7	0.000	0.000	0.000	0.000	-1.391	0.000	0.000	0.000
MAPK8	0.000	0.000	0.000	0.000	0.000	-0.963	0.000	0.000
COX7A2L	0.000	0.000	-1.815	0.000	0.000	0.000	0.000	0.000
RAB11A	0.000	0.000	0.000	0.000	0.000	-0.769	0.000	0.000
FUT11	0.000	0.000	0.000	0.000	-1.214	0.000	0.000	0.000
KIT	0.000	0.000	0.000	0.000	-5.522	0.000	0.000	0.000
PKN3	0.000	0.000	0.000	0.000	0.000	2.830	0.000	1.780
CR2	4.667	0.000	0.000	0.000	0.000	0.000	0.000	0.000
ATM	3.165	0.000	1.935	3.630	1.384	2.800	0.000	1.104
TBC1D4	0.000	0.000	1.690	3.066	2.043	3.900	0.000	0.000
CAMK2N1	0.000	0.989	0.000	0.000	0.000	0.000	0.000	0.000

CTSL	1.660	0.000	0.000	0.000	0.000	0.000	0.000	0.000
HCAR3	0.000	3.645	0.000	0.000	0.000	0.000	0.000	0.000
PRPF19	0.000	0.000	0.000	0.000	0.000	-0.629	0.000	0.000
BAX	0.000	0.671	0.000	0.000	0.000	0.000	0.000	0.000
TGFB2	0.000	4.320	0.000	0.000	0.000	0.000	0.000	0.000
SLK	0.000	0.000	0.000	0.000	0.000	-0.787	0.000	0.000
UBE2W	0.000	0.000	0.872	0.000	0.000	0.000	0.000	0.000
WASL	-4.558	0.000	0.000	0.000	0.000	0.000	0.000	0.000
YWHAG	0.000	0.000	0.000	-1.692	0.000	-4.273	0.000	0.000
BFAR	0.000	0.000	0.000	0.000	0.000	0.000	0.000	0.937
FDFT1	-4.812	0.000	0.000	0.000	0.000	5.500	0.000	0.000
APIP	0.000	0.000	0.000	2.026	0.000	0.000	0.000	0.000
PTCH2	0.000	0.000	0.000	2.618	2.635	0.000	0.000	3.575
IP6K2	1.655	0.000	0.000	0.000	0.000	0.000	0.000	0.000
HCRTR2	0.000	0.000	0.000	0.000	4.851	6.912	0.000	0.000
GBP1	0.000	0.000	0.000	0.000	0.000	0.000	-0.859	-0.930
P2RY4	5.885	0.000	0.000	0.000	0.000	0.000	0.000	0.000
VSIG4	0.000	2.330	0.000	2.861	0.000	0.000	0.000	0.000
TDO2	0.000	0.000	0.000	0.000	0.000	5.563	0.000	0.000
CBLL1	0.000	0.000	0.000	0.000	0.000	0.000	-1.302	0.000
TNFRSF4	3.512	0.000	0.000	3.032	0.000	3.592	0.000	4.034
HTR1E	0.000	1.723	1.879	3.672	1.716	3.879	-2.740	3.944
JPH3	0.000	0.000	-1.667	0.000	0.000	0.000	0.000	-1.412
MAN1A1	-2.052	0.000	0.000	0.000	0.000	0.000	0.000	0.000
VIPR2	0.000	4.583	0.000	0.000	0.000	0.000	0.000	0.000
RICTOR	-0.957	0.000	0.731	0.000	0.000	0.000	0.000	0.000
YWHAH	-5.306	0.000	-0.886	0.000	0.000	-5.285	0.000	0.000
ATP5C1	3.210	0.000	0.000	0.000	0.000	0.000	0.000	0.000
UBA1	-1.108	0.000	0.000	0.000	0.000	-0.870	0.000	0.000
VDAC3	0.000	0.000	0.000	0.000	0.000	0.000	0.000	-2.189
MSX1	0.000	0.000	0.000	0.000	0.000	3.124	0.000	0.000
PVRL3	0.000	0.000	0.000	0.000	0.000	-4.677	0.000	0.000
EP300	0.000	0.000	0.000	0.000	0.000	-1.469	0.000	0.000
OGA	-2.483	0.000	0.000	0.000	0.000	-1.385	0.000	0.000
UBA6	-1.900	0.000	0.000	0.000	0.000	-0.871	0.000	0.000
AZI2	1.685	-1.264	0.000	0.000	0.000	0.000	0.000	0.000
JUP	0.000	-2.057	0.000	0.000	0.000	0.000	0.000	0.000
DFFA	0.000	0.000	-1.678	0.000	0.000	0.000	0.000	0.000
ABHD5	0.000	1.326	0.000	0.000	0.000	0.000	0.000	0.000
AQP1	3.864	0.000	0.000	0.000	0.000	0.000	0.000	0.000
TLE1	1.978	0.000	-0.813	0.000	0.000	0.000	0.000	0.000
SOCS2	0.000	0.000	0.000	2.225	0.000	0.000	0.000	0.000
SARM1	0.000	0.000	0.000	0.000	0.000	0.000	1.102	0.000

SGCD	3.114	0.000	0.000	3.018	0.000	3.207	-3.915	2.594
TRAF6	0.000	0.000	0.000	0.000	0.000	2.430	0.000	0.000
FTH1	1.003	0.000	0.000	0.000	0.000	0.000	0.978	0.000
NUMBL	0.000	1.514	0.000	1.727	0.000	3.562	0.000	0.000
BIRC3	2.294	1.476	0.000	2.158	1.927	2.355	0.000	2.648
ITGA6	0.000	0.000	0.000	0.000	0.000	0.000	-2.351	0.000
ITGB8	3.322	0.000	0.000	3.336	0.000	3.679	0.000	1.831
GABRG3	5.333	0.000	0.000	0.000	0.000	0.000	0.000	0.000
CCNC	-3.183	0.000	0.000	0.000	0.000	1.078	0.000	0.000
AHR	0.000	-2.878	0.000	0.000	0.000	0.000	0.000	0.000
NFKBIA	1.792	0.910	0.000	0.000	0.000	2.474	0.000	0.000
RPS6KA5	0.000	0.000	0.000	0.000	0.000	0.000	-1.174	0.000
AURKA	0.000	0.000	0.000	-1.035	0.000	0.000	0.000	0.000
TLX3	0.000	2.786	0.000	3.315	0.000	0.000	0.000	0.000
ATF1	-0.985	0.000	0.000	0.000	0.000	-1.940	0.000	0.000
BCL2L2	-1.784	0.000	0.000	0.000	0.000	-1.859	0.000	0.000
SMAD9	0.000	0.000	0.000	0.000	-0.912	-4.302	0.000	-0.706
GABRB3	0.000	0.000	0.000	0.000	3.981	0.000	0.000	0.000
GABBR2	0.000	0.000	0.000	0.000	0.000	0.000	0.000	4.637
MCL1	0.000	0.000	0.000	1.646	0.000	0.000	0.000	0.000
TNFRSF21	0.000	0.000	0.000	0.000	0.000	0.000	-3.538	0.000
EIF2B1	-2.352	0.000	0.000	0.000	0.000	0.000	0.000	0.000
PGK1	1.983	0.000	0.000	0.000	0.000	2.445	0.000	0.000
CEL	0.000	0.000	0.000	0.000	-3.796	0.000	0.000	0.000
PAK4	1.517	0.000	0.000	0.000	0.000	0.000	0.000	0.000
GNG11	1.395	0.000	0.000	0.000	0.000	0.000	0.000	0.000
RAC3	3.002	0.000	-1.081	0.000	0.000	0.000	0.000	0.000
CARD16	0.000	0.000	0.000	0.000	-1.097	0.000	0.000	0.000
UBE2Q2	0.000	0.000	0.000	0.000	0.000	-0.990	0.000	0.000
PDLIM7	0.000	0.000	-0.836	0.000	0.000	-1.903	0.000	0.000
DLD	0.000	0.000	0.000	0.000	0.000	-0.745	0.000	0.000
TRPM2	0.000	1.521	0.000	0.000	0.000	0.000	0.000	0.000
TAB3	1.698	0.000	0.000	0.000	0.000	0.000	0.000	0.000
MAPRE2	0.000	0.000	0.000	0.000	0.000	-1.649	0.000	0.000
PSMF1	-0.597	0.000	0.000	0.000	0.000	0.000	0.000	0.000
TCF7	1.411	0.000	0.000	0.000	0.000	0.000	-1.260	-0.742
ADCY4	0.000	0.000	0.000	2.786	0.000	4.613	0.000	3.530
TYMS	-1.207	0.000	0.000	0.000	0.000	0.000	0.000	0.000
WWOX	2.336	1.137	0.000	1.627	0.000	0.000	-1.139	0.000
CMTM4	2.742	0.000	0.000	2.271	0.000	0.000	0.000	3.811
CCNB2	-1.050	0.000	0.642	0.000	0.000	0.000	0.000	0.000
CSNK1D	0.975	0.000	0.000	0.000	0.000	0.000	0.000	0.000
IL11RA	0.000	2.227	0.000	0.000	0.000	0.000	0.000	2.550

FSHB	2.540	0.000	1.663	3.152	2.159	4.134	-2.090	4.785
DGKA	0.000	0.000	0.000	0.000	0.000	-3.136	0.000	0.000
CPA3	6.684	0.000	0.000	0.000	0.000	0.000	-4.231	0.000
CARD11	0.000	1.183	1.211	0.000	0.000	0.000	0.000	0.907
SERTAD1	0.000	0.000	0.000	0.000	0.000	1.093	0.000	0.000
PIP	0.000	0.000	0.000	4.791	0.000	0.000	0.000	0.000
PPL	3.976	1.760	2.108	3.433	1.675	3.095	0.000	2.458
JAM3	1.573	0.000	0.000	0.000	0.000	1.649	0.000	0.000
MCM4	0.000	0.000	0.000	0.000	-0.586	-1.257	0.000	0.000
CCL14	0.000	2.872	0.000	3.017	0.000	0.000	0.000	3.221
EPHA2	0.000	0.000	0.000	-2.008	0.000	0.000	0.000	0.000
CCL7	0.000	2.760	0.000	0.000	0.000	0.000	0.000	0.000
MCEE	3.745	0.000	0.000	2.578	0.000	3.299	-2.861	0.000
PPP1CB	2.786	0.000	0.000	1.996	1.278	0.000	0.000	0.000
C4BPB	0.000	3.727	0.000	0.000	0.000	0.000	0.000	0.000
PXN	0.000	0.000	0.000	0.000	0.000	-1.150	0.000	0.000
B4GALT5	0.000	0.000	0.000	0.000	0.000	1.814	0.000	0.000
CRHR1	0.000	0.000	0.650	1.274	0.000	0.000	0.000	0.000
PRICKLE1	0.000	0.000	-3.428	2.206	0.000	0.000	0.000	0.000
CHRNA10	0.000	0.000	1.449	3.149	3.024	3.668	0.000	4.420
FH	-1.432	0.000	0.000	0.000	0.000	0.000	0.000	0.000
CYP26B1	0.000	0.000	0.000	0.000	0.000	0.000	0.000	-3.591
DBN1	0.000	0.000	0.000	0.000	0.000	-1.127	0.000	0.000
FOXC2	0.000	0.000	0.000	0.000	0.000	0.000	0.000	0.800
MBTPS1	0.000	2.002	0.000	0.000	0.000	4.712	-3.462	0.000
APOC3	4.343	0.000	0.000	2.101	3.633	0.000	0.000	2.873
ARHGEF2	0.000	0.000	0.000	0.000	0.000	-1.075	0.000	0.000
GADD45G	0.000	0.000	0.000	0.000	0.000	3.207	0.000	0.000
LAMTOR1	0.000	-2.026	0.000	0.000	0.000	0.000	0.000	0.000
SMAD6	0.000	0.000	0.000	2.194	0.000	0.000	0.000	2.159
AHCY	0.000	0.000	0.000	0.000	0.000	0.000	-3.866	0.000
MCOLN3	0.000	0.000	0.000	0.000	0.000	-3.345	0.000	0.000
IFIT2	0.000	0.000	0.954	0.000	0.000	-4.740	0.000	0.000
JUN	3.880	0.000	0.000	0.000	0.000	3.862	0.000	0.000
GP1BA	0.000	0.000	0.000	3.852	0.000	0.000	0.000	0.000
AUH	0.000	0.000	0.000	0.000	0.000	0.000	0.000	2.718
PDYN	0.000	0.000	0.000	0.000	0.000	4.752	0.000	0.000
GNAQ	0.000	0.768	1.032	1.753	0.000	2.120	0.000	2.646
CSK	0.000	0.000	0.000	-3.890	0.000	0.000	0.000	0.000
GABARAP	0.000	0.881	0.000	0.000	0.000	0.000	0.000	0.000
HSPA1A	0.000	0.000	0.000	0.000	0.000	2.565	0.000	0.000
PLA2G12B	0.000	0.000	0.000	3.434	0.000	0.000	0.000	0.000
TXN2	0.000	0.000	0.000	-1.983	0.000	0.000	0.000	0.000

ARNT2	2.710	0.000	0.000	1.219	0.000	0.000	0.000	0.000
DYNC1H1	0.000	0.000	-0.806	0.000	0.000	0.000	0.000	0.000
NEIL3	0.000	1.646	1.692	3.160	1.751	2.403	0.000	1.655
NR1H3	5.016	0.000	0.000	0.000	0.000	0.000	0.000	0.000
LAP3	-2.379	0.000	0.000	0.000	0.000	0.000	0.000	0.000
GCLM	0.000	0.000	0.000	0.000	0.000	-1.583	0.742	0.000
JAG1	5.052	0.000	0.000	1.941	0.000	3.793	0.000	0.000
SDHD	0.000	-3.320	0.000	0.000	0.000	0.000	0.000	0.000
UBE2J1	-0.812	0.000	0.000	0.000	0.000	-0.750	0.000	0.000
SDHA	0.000	0.000	0.000	0.000	0.000	-1.532	0.000	0.000
LTBP1	0.000	0.000	0.000	0.000	0.000	-1.623	0.000	0.000
MYLK	-1.763	-0.593	-0.855	-1.074	0.000	-1.395	0.000	-0.767
PTTG1	0.000	0.000	0.000	2.011	1.878	3.189	0.000	2.801
NUMB	0.000	0.000	1.066	0.000	0.000	0.000	1.133	0.000
RARA	0.000	3.667	0.000	0.000	0.000	0.000	0.000	0.000
SNX17	0.000	0.000	0.000	0.000	-3.908	0.000	0.000	-2.019
PKN2	0.000	0.000	0.000	0.000	0.000	2.335	0.000	0.000
NPW	0.000	0.000	0.000	3.717	0.000	0.000	0.000	0.000
IDH3B	0.000	1.344	0.000	1.595	0.000	0.000	0.000	0.000
ALG13	0.000	0.000	0.000	0.000	0.000	-4.228	0.000	0.000
CXCL12	1.161	-0.950	0.000	0.000	0.000	0.000	-0.730	0.000
SOCS3	0.000	0.000	0.000	0.000	0.000	1.644	0.000	0.000
PPP1R14A	3.102	1.980	0.000	1.595	0.000	2.777	0.000	0.000
BIRC2	-0.946	0.000	0.000	0.000	0.000	0.000	0.000	0.000
HCN2	0.000	0.000	0.000	2.286	0.000	0.000	0.000	2.077
CNGA2	0.000	0.000	0.000	0.000	0.000	6.550	0.000	0.000
ANAPC10	-0.706	0.000	0.000	0.000	0.000	0.000	0.000	0.000
MAP3K3	0.000	0.000	0.000	0.000	0.000	-4.792	0.000	0.000
NFATC3	0.000	-3.172	0.000	0.000	0.000	0.000	0.000	-2.251
MECOM	0.000	0.000	0.000	0.000	-4.101	0.000	0.000	0.000
DAGLA	1.893	-1.111	0.000	0.000	0.000	0.000	0.000	0.000
CASP10	0.000	0.000	0.000	1.213	1.611	0.000	0.000	0.000
ITGA1	0.000	0.000	0.000	0.000	0.000	-4.528	0.000	0.000
CACNA1S	4.386	1.587	0.000	2.699	0.000	4.289	-4.291	3.662
VDAC2	0.000	0.000	0.000	0.000	0.000	2.567	0.000	0.000
SYVN1	0.000	0.000	0.000	0.000	0.000	1.693	0.000	0.000
DFFB	0.000	0.000	1.147	0.000	0.000	0.000	0.000	0.000
DAAM1	2.834	0.000	0.000	0.000	0.000	0.000	0.000	0.000
IFI16	3.089	0.000	0.000	0.000	0.000	0.000	0.000	0.000
PRKN	0.000	2.669	0.000	3.332	0.000	3.654	0.000	0.000
SLC22A8	4.682	0.000	0.000	4.359	0.000	0.000	0.000	0.000
CREB3L4	3.568	0.000	0.000	-2.963	0.000	0.000	0.000	0.000
CACNA1I	4.402	0.000	0.000	2.662	0.000	3.694	-3.233	4.339

GNG4	0.000	0.000	0.000	0.000	0.000	-3.216	0.000	0.000
CACNA2D2	0.000	3.720	0.000	0.000	0.000	0.000	0.000	0.000
SNAI1	0.000	2.177	0.000	0.000	0.000	5.172	0.000	0.000
DDIT4L	0.000	2.452	0.000	0.000	-2.333	0.000	0.000	0.000
ATP6V0A2	0.000	0.000	2.246	0.000	0.000	0.000	1.254	0.000
CCT4	-4.921	0.000	0.000	0.743	0.000	-4.975	0.000	-0.886
PKM	0.000	0.000	-0.817	0.000	0.000	0.000	0.000	0.000
PPP5D1	0.000	0.000	0.000	1.784	0.000	2.915	0.000	0.000
FLT3LG	0.000	0.000	0.000	0.000	0.000	2.761	0.000	1.905
TNNT2	3.637	0.000	1.402	3.311	2.702	4.483	-1.573	4.648
ACTA2	-1.476	0.000	0.000	0.852	0.000	0.000	0.000	0.691
IL22RA2	2.176	0.000	0.000	2.944	2.902	4.467	-1.366	3.804
TSHZ1	0.000	0.000	0.000	2.277	0.000	0.000	0.000	0.000
PERP	0.000	0.000	0.000	0.000	0.000	0.000	0.000	-3.237
DCTN5	1.854	0.000	0.000	0.000	0.000	0.000	0.000	0.000
TNFRSF10C	2.943	0.000	0.000	3.168	2.089	4.293	-1.330	4.015
SLC27A5	0.000	0.000	0.000	0.000	0.000	7.666	0.000	0.000
CNOT2	0.000	0.000	0.000	0.768	0.000	1.148	0.000	0.000
TRIP10	0.000	0.000	0.000	0.000	0.000	-1.825	0.000	0.000
COL8A1	0.000	0.000	-0.925	0.000	0.000	0.000	-1.171	-0.824
AQP9	0.000	2.538	0.000	0.000	0.000	0.000	0.000	0.000
P2RX1	0.000	0.000	0.000	0.000	0.000	0.000	0.000	4.843
MVD	0.000	0.000	0.000	0.000	-3.246	0.000	0.000	0.000
FBXO32	0.000	2.514	0.000	0.000	0.000	0.000	0.000	0.000
FGF12	0.000	0.000	0.000	-2.780	0.000	0.000	0.000	0.000
ACP1	3.070	0.000	0.000	0.000	0.000	0.000	0.000	0.000
SLC4A2	0.000	2.441	0.000	2.744	0.000	3.594	0.000	3.038
CLEC3B	0.000	0.000	0.000	0.000	0.000	5.002	0.000	0.000
RXFP3	0.000	0.000	0.000	2.924	0.000	4.298	0.000	0.000
TM6SF1	0.000	0.000	0.000	0.000	-5.126	0.000	0.000	0.000
TTN	0.000	1.874	0.000	3.037	2.149	3.211	0.000	2.734
IL10RB	0.000	0.000	0.000	0.000	-4.104	0.000	0.000	0.000
SNAP23	-1.295	0.000	0.000	0.000	0.000	-1.274	0.000	0.000
MMP8	0.000	4.971	0.000	0.000	0.000	0.000	0.000	0.000
IFIT1	3.370	0.000	1.412	2.839	1.243	4.310	0.000	3.004
UGP2	0.000	0.000	0.000	0.000	0.000	1.876	0.000	0.000
HDAC6	0.000	0.000	0.000	0.000	0.000	4.660	0.000	0.000
PYCR1	0.000	1.115	0.000	1.114	0.000	0.000	0.000	1.929
SFN	0.000	0.000	0.000	0.000	0.000	5.881	0.000	0.000
RAB5A	2.263	0.000	0.000	0.000	0.000	0.000	0.000	0.000
ESR2	3.812	0.000	0.000	2.410	0.000	0.000	0.000	0.000
TFPI	0.000	0.000	0.000	1.901	0.977	0.000	0.000	0.000
P2RX5	0.000	-3.429	0.000	0.000	-4.338	0.000	0.000	0.000

MCCC2	0.000	0.000	0.000	0.000	0.000	-4.341	0.000	0.000
DNAJC3	0.000	0.000	0.586	0.000	0.000	0.000	0.000	0.000
FADD	-1.516	0.000	0.000	0.000	0.000	0.000	0.000	0.000
PPP2CB	0.000	0.785	0.000	0.000	0.000	0.000	0.000	0.000
ARRDC3	0.000	0.000	1.979	0.000	0.000	0.000	0.000	0.000
TRIB3	0.000	0.867	0.983	0.000	0.000	2.029	0.000	0.000
TCL1A	0.000	0.000	0.000	0.000	0.000	7.480	0.000	0.000
IL20RA	3.395	0.000	1.500	3.077	1.385	3.790	-2.519	2.518
HLA-C	0.000	4.225	0.000	0.000	0.000	0.000	0.000	0.000
SLC38A9	0.000	0.000	0.000	0.000	0.000	1.005	0.000	0.000
ICOS	6.194	0.000	0.000	3.123	0.000	0.000	0.000	0.000
NCOA3	3.429	1.495	0.000	2.596	2.146	0.000	0.000	3.526
CFLAR	0.000	0.000	0.000	-0.918	0.000	0.000	0.000	0.000
APBA1	2.326	1.400	0.000	0.000	1.175	0.000	0.000	1.095
STK11	0.000	0.000	0.000	0.000	0.000	1.072	0.000	0.000
NFYA	2.509	0.000	0.000	1.188	1.002	2.165	0.000	1.364
EDIL3	0.000	0.000	0.000	0.000	0.000	0.000	-0.887	-0.989
LDHC	8.422	0.000	0.000	0.000	0.000	0.000	0.000	0.000
SORT1	0.000	0.000	0.000	2.096	0.000	2.025	0.000	0.000
ATP2B1	0.000	0.000	0.000	0.931	0.000	2.064	0.000	0.000
ACLY	0.000	0.000	-0.790	0.000	0.000	0.000	0.000	0.000
RAG1	0.000	3.470	0.000	0.000	4.292	0.000	0.000	0.000
EGLN2	0.000	0.000	0.000	-2.243	0.000	0.000	0.000	0.000
DKK3	0.000	0.000	0.000	0.000	0.000	-0.763	0.000	0.000
ABL2	2.011	1.647	0.000	1.407	2.178	0.000	0.000	0.000
TPM1	0.000	0.000	-0.801	0.000	0.000	-0.796	-0.651	0.000
KLF4	0.000	0.000	0.000	1.125	0.000	2.709	0.000	0.000
HSPB1	0.000	-0.750	0.000	0.000	0.000	0.000	0.000	0.000
CD1D	0.000	4.169	0.000	0.000	0.000	0.000	0.000	0.000
MGST2	2.087	0.000	0.000	0.000	0.000	0.000	0.000	0.000
EIF4E	0.000	0.000	0.000	0.000	-0.896	0.000	0.000	0.000
SCN1B	0.000	0.000	0.000	2.496	1.521	0.000	0.000	1.862
PRCC	0.000	0.000	0.000	0.000	0.000	1.756	0.000	0.000
ACTG1	0.000	0.000	-0.701	-1.115	0.000	0.000	0.000	-1.384
PIK3CD	0.000	0.668	0.000	0.734	0.000	0.000	0.000	0.000
POMC	0.000	2.419	0.000	1.853	2.415	0.000	0.000	0.000
ULK1	0.000	0.000	0.000	0.000	0.000	0.000	1.401	0.000
ZFP91	2.292	0.000	1.074	2.273	0.641	2.156	0.000	0.646
ABCG8	0.000	0.000	0.000	0.000	0.000	7.168	0.000	0.000
MDC1	-4.499	0.000	0.000	0.000	0.000	0.000	0.000	0.000
PRLHR	0.000	0.000	0.000	0.000	0.000	0.000	0.000	4.382
PTH2R	0.000	0.000	0.000	0.000	0.000	5.072	0.000	0.000
ZEB1	2.238	0.000	0.898	0.000	0.000	0.000	0.000	0.000

SEH1L	0.813	0.000	0.000	0.000	0.000	0.000	0.000	0.000
QARS	0.000	0.000	0.000	0.000	0.000	-1.177	0.000	0.000
BRMS1	-2.443	0.000	0.000	0.000	0.000	0.000	0.000	0.000
MAPKAP1	0.000	0.000	0.000	0.617	-0.809	0.000	0.000	0.000
PHLPP2	1.655	0.000	0.817	1.508	0.000	1.817	0.000	0.000
RHOA	0.000	0.000	0.000	0.000	0.000	-0.755	0.000	0.000
PARD6G	0.000	3.032	0.000	0.000	-4.122	0.000	0.000	0.000
CDKN2D	0.000	0.000	0.000	0.000	0.000	2.401	0.000	0.000
S1PR4	0.000	4.771	0.000	4.261	0.000	0.000	0.000	0.000
UBE2D2	-0.611	0.000	0.000	0.000	0.000	0.000	0.000	0.000
C3AR1	0.000	2.724	0.000	3.122	0.000	3.545	0.000	0.000
H2AFX	0.000	0.000	0.000	0.000	-5.518	0.000	0.000	0.000
TSC1	0.000	0.000	0.000	0.000	0.000	1.663	0.000	0.000
NDUFS2	0.000	0.938	0.000	0.000	0.000	0.000	0.000	0.000
EEF2	-0.933	0.000	0.000	0.000	0.000	0.000	0.000	0.000
CYP3A5	5.464	2.225	0.000	4.016	0.000	0.000	0.000	2.461
FARP2	-2.346	-0.605	0.000	0.000	0.000	-1.319	0.000	0.000
SLC27A6	0.000	4.293	0.000	4.715	0.000	0.000	0.000	3.623
COMP	6.138	0.000	0.000	0.000	0.000	0.000	0.000	0.000
PRDM2	0.000	0.000	1.454	3.238	0.000	3.623	-2.545	4.240
ATG4C	2.143	0.000	0.000	0.000	0.000	0.000	0.000	0.000
APH1A	0.000	0.000	0.000	0.000	0.000	0.000	0.000	-1.046
GNA13	0.000	0.000	0.000	0.000	0.000	-5.984	0.000	0.000
ALDH2	0.983	0.000	0.000	0.000	0.000	0.000	0.000	0.000
MYBL1	-1.334	0.000	0.000	-0.720	0.000	-0.898	0.000	0.000
NDUFA6	0.000	0.000	0.000	0.000	0.000	1.397	0.000	0.000
TBC1D1	0.000	0.000	-0.700	0.000	0.000	0.000	0.000	0.000
CDK7	0.000	0.000	0.000	0.000	0.000	0.908	0.000	0.000
FPR1	0.000	3.693	0.000	3.809	0.000	0.000	0.000	0.000
ATRIP	0.000	0.000	0.000	0.000	-1.452	0.000	0.000	0.000
SCD	3.032	1.227	0.000	2.300	1.675	3.118	0.000	0.000
UBE3B	0.000	0.000	0.000	0.000	0.000	1.357	0.000	0.000
PLCE1	0.000	0.000	0.857	2.444	0.845	2.569	0.000	1.754
C1QC	0.000	3.274	0.000	4.324	0.000	0.000	0.000	3.193
CHUK	0.000	0.000	0.000	0.000	0.000	0.779	0.000	0.000
SCNM1	-4.820	0.675	0.000	0.000	0.000	1.464	0.000	0.000
SORBS1	0.000	0.000	0.000	0.000	0.000	7.078	0.000	0.000
PIK3CG	0.000	0.000	1.657	2.856	0.000	0.000	0.000	0.000
IL19	0.000	0.000	0.000	3.037	0.000	0.000	0.000	0.000
HTATIP2	0.000	0.000	0.631	0.000	0.000	0.000	0.000	0.000
HSD3B1	0.000	0.000	0.000	4.879	4.373	0.000	0.000	0.000
UNG	0.000	0.000	0.000	0.000	0.000	-4.794	0.000	0.000
LMO7	0.000	0.000	0.000	0.000	0.000	-4.633	0.000	0.000

CASP8	0.000	0.000	0.780	0.000	0.000	0.000	0.000	0.000
NDUFB1	-1.692	0.000	0.000	0.000	0.000	0.000	0.000	0.000
TLN1	0.000	0.000	0.000	0.000	0.000	0.815	0.000	0.000
FLNB	0.000	-0.678	-0.760	0.000	0.000	-6.274	0.000	0.000
CALM3	-0.641	0.000	0.000	0.000	0.000	0.000	0.000	0.000
MNDA	0.000	3.054	0.000	3.807	0.000	0.000	0.000	0.000
TGFBRAP1	0.000	-0.780	0.000	0.000	0.000	0.000	0.000	0.000
ITPR2	-1.474	0.000	-0.948	0.000	0.000	0.000	0.000	0.000
NEIL2	0.000	0.000	0.000	3.014	0.000	0.000	0.000	0.000
MC5R	0.000	0.000	0.000	3.552	0.000	0.000	0.000	0.000
STXBP2	0.000	0.000	0.000	0.000	0.000	0.000	-3.407	0.000
GLB1	-2.290	0.000	0.000	0.000	0.000	0.000	0.000	0.000
FAT4	2.754	0.000	0.000	0.000	0.000	0.000	0.000	0.000
AGTRAP	0.000	0.800	0.000	2.069	0.993	0.000	0.000	0.674
ENO2	0.000	0.000	0.775	0.000	0.000	0.000	0.000	0.000
TNFAIP3	0.000	0.000	0.000	0.000	0.000	0.000	-3.813	0.000
CYP2S1	3.311	1.904	0.000	2.413	2.418	3.726	0.000	3.384
GPT	0.000	0.000	0.000	0.000	0.000	4.055	0.000	3.935
PFKL	0.000	0.000	0.000	0.000	0.000	-4.512	0.000	0.000
GABRA3	3.069	2.254	0.000	3.078	0.000	4.535	-1.778	2.789
COL3A1	0.000	0.000	0.000	0.000	0.000	0.000	0.000	-2.472
PTPN2	0.000	0.000	0.000	0.000	0.000	-1.445	0.000	0.000
TRIM10	3.644	3.492	0.000	4.317	0.000	0.000	0.000	0.000
CBLC	0.000	0.000	0.000	0.000	5.209	0.000	0.000	0.000
ANAPC11	0.000	0.000	-1.109	0.000	0.000	0.000	0.000	0.000
ESAM	3.505	0.000	0.000	0.000	3.377	0.000	-3.554	3.497
LRP1	1.353	0.000	0.000	0.000	0.000	-3.142	0.000	0.000
PPP2CA	0.000	0.000	-0.623	-0.767	0.000	0.000	0.000	0.000
PDK2	0.000	0.000	0.000	0.000	0.000	1.809	0.000	0.000
NOS2	0.000	0.000	0.000	0.000	0.000	7.753	0.000	0.000
RAD51C	0.000	-0.839	0.000	0.000	0.000	0.000	0.000	0.000
RLN3	0.000	0.000	0.000	0.000	0.000	6.547	0.000	0.000
RARRES2	-2.273	0.000	0.000	0.000	0.000	0.000	0.000	-1.119
CCNG2	1.561	0.798	1.329	1.175	0.671	0.000	0.000	0.000
HLA-DRB3	0.000	0.000	0.000	3.483	0.000	0.000	0.000	4.257
LY86	4.590	0.000	0.000	2.785	2.605	0.000	0.000	0.000
PARVA	0.000	0.000	0.000	0.000	0.000	-0.747	0.000	0.000
LPP	0.000	0.000	-2.478	0.000	0.000	0.000	0.000	0.000
HNRNPUL1	-0.708	0.000	0.000	0.000	0.000	-1.313	0.000	0.000
PPP1R12B	2.558	0.000	0.000	2.039	0.000	0.000	0.000	0.000
CASP12	5.204	1.673	0.000	3.071	2.513	0.000	-1.828	2.796
CD3E	0.000	3.246	0.000	0.000	0.000	0.000	0.000	0.000
SGCE	0.000	0.000	0.828	0.000	0.000	0.000	0.000	0.000

TNFSF13B	4.716	1.723	1.242	3.023	2.975	4.290	0.000	3.191
UGT2B4	0.000	0.000	0.000	0.000	0.000	7.490	0.000	0.000
MGLL	0.000	0.000	-1.320	0.000	0.000	2.050	0.000	0.000
SETBP1	6.707	0.000	0.000	0.000	0.000	0.000	0.000	0.000
LAMA5	0.000	0.000	0.000	1.396	0.000	0.000	0.000	0.000
MLXIP	0.000	0.000	0.000	0.000	0.000	0.000	0.000	-3.619
CACNG5	0.000	0.000	0.000	0.000	0.000	7.490	0.000	0.000
RPA3	2.065	0.000	1.438	3.036	1.714	3.384	0.000	3.078
LDHAL6A	3.398	0.000	2.160	3.520	0.000	3.741	-1.826	2.694
PTPN13	0.000	0.000	0.000	0.000	0.000	-1.962	0.000	0.000
MYC	0.000	0.000	-1.657	0.000	0.000	0.000	0.000	0.000
SDC4	0.000	0.000	0.000	0.000	0.000	0.000	0.000	-1.398
MUTYH	0.000	0.000	0.000	0.000	-2.057	0.000	0.000	0.000
BCL10	0.000	0.000	0.000	0.000	0.000	-1.509	0.000	0.000
PHKA2	0.000	-3.997	0.000	0.000	-5.656	0.000	0.000	0.000
IL1B	0.000	0.000	0.000	0.000	0.000	0.000	0.000	3.147
FOSL1	0.000	0.000	0.000	0.000	0.000	0.000	0.614	0.000
SLC27A2	4.504	2.512	1.400	2.788	1.263	2.355	-1.829	1.474
MRE11A	0.000	-0.771	0.000	0.000	0.000	0.000	0.000	0.000
NEUROD1	9.053	0.000	0.000	0.000	0.000	0.000	0.000	0.000
ENO3	2.076	1.458	0.000	1.497	0.000	1.646	0.000	1.231
NDUFB2	4.458	0.000	0.000	0.000	0.000	0.000	0.000	0.000
FRAT1	0.000	0.000	0.000	0.000	-1.186	0.000	0.000	0.000
VAMP8	0.000	0.000	0.000	2.359	0.000	0.000	0.000	0.000
COL12A1	0.000	0.000	0.000	-0.691	0.000	-4.127	-0.603	0.000
NPR1	0.000	0.000	0.000	0.000	0.000	0.000	0.000	-4.124
PFKP	0.000	0.000	0.000	0.000	0.000	3.149	0.000	0.000
TGIF1	0.000	4.135	0.000	0.000	0.000	0.000	0.000	0.000
NQO1	0.000	0.000	0.000	0.000	0.000	0.000	1.175	0.000
GML	7.337	4.931	0.000	0.000	0.000	0.000	0.000	0.000
TEAD2	0.000	0.000	0.000	1.526	0.000	0.000	0.000	0.000
GNG7	0.000	1.372	0.000	0.000	0.000	0.000	0.000	0.000
CLIP1	0.000	0.000	0.000	-0.645	0.000	0.000	0.000	0.000
FGF14	3.610	0.000	0.000	2.862	1.830	0.000	-4.471	0.000
EIF5B	1.519	0.000	0.000	0.000	0.000	1.703	0.000	0.000
DYNC1I2	-1.023	0.000	0.000	0.000	0.000	-1.076	0.000	0.000
PAK2	0.000	0.000	1.870	0.000	0.000	4.396	0.000	0.000
FABP4	0.000	0.000	0.000	2.992	2.483	0.000	0.000	3.031
NOTCH1	0.000	0.000	0.000	1.604	0.000	0.000	0.000	0.000
CLDN7	0.000	0.000	0.000	3.388	0.000	5.076	0.000	0.000
PLCG1	2.973	0.000	0.000	2.695	0.000	2.887	0.000	2.505
ATF3	0.000	0.000	0.000	1.909	0.000	3.916	0.000	0.000
JAK2	1.197	0.672	0.694	2.042	0.000	1.980	0.000	1.337

ADORA3	2.827	0.000	1.783	2.879	0.000	3.173	-2.401	3.449
PSMD5	-1.854	0.000	0.000	0.000	0.000	-3.239	0.000	0.000
CD59	0.000	0.000	0.000	0.000	0.000	-0.981	0.000	0.000
PPP5C	0.000	0.000	0.000	0.000	-1.649	0.000	0.000	-1.077
HCN4	2.909	0.000	2.058	3.645	0.000	4.030	0.000	2.399
PMS1	0.000	0.000	0.000	0.000	0.000	-1.468	0.000	0.000
FKBP11	3.077	0.000	0.000	0.000	0.000	0.000	0.000	0.000
TACR3	0.000	4.719	0.000	4.499	0.000	0.000	0.000	0.000
ZFYVE16	0.000	0.000	0.641	0.000	0.000	0.000	0.000	0.000
EDN1	0.000	0.000	-1.074	0.000	0.000	-4.427	-1.497	0.000
MTDH	0.000	0.000	0.000	-0.756	0.000	0.000	0.000	0.000
RNF168	1.038	0.000	0.000	0.000	0.000	0.000	0.000	0.000
SRM	0.000	0.000	-0.647	0.000	0.000	0.000	0.000	0.000
RBBP8	0.000	0.000	0.000	0.000	0.000	-1.790	0.000	0.000
MAP2K7	0.000	0.000	0.000	2.131	0.000	3.122	0.000	3.680
TRPV5	0.000	2.583	0.000	2.968	0.000	2.627	-2.330	0.000
PMAIP1	0.000	0.000	0.781	0.000	0.000	0.000	0.000	0.674
CYP11B2	4.184	0.000	0.000	2.577	0.000	0.000	0.000	2.492
PLK2	-1.384	0.000	-0.750	-0.797	0.000	0.000	0.000	-0.704
RND3	0.718	0.000	0.000	0.000	0.000	1.387	0.000	0.000
PHKB	0.000	0.000	0.000	-1.242	0.000	-4.576	0.000	0.000
ADIPOQ	5.929	0.000	0.000	3.253	0.000	0.000	0.000	0.000
LMO1	0.000	0.000	0.000	0.000	3.386	0.000	0.000	0.000
UBE2U	0.000	0.000	0.000	4.069	0.000	0.000	0.000	0.000
IFNA4	3.724	3.156	0.000	2.969	2.553	3.509	0.000	3.299
RDH10	0.000	2.281	1.671	2.413	0.000	0.000	0.000	2.591
ILF2	0.000	0.000	0.000	0.882	0.000	1.070	0.000	1.016
B4GALT1	-2.291	0.000	0.000	0.000	0.000	-2.079	0.000	0.000
LZTR1	2.130	0.000	0.000	0.000	0.000	0.000	0.000	0.000
FGF7	0.000	0.000	0.000	0.000	0.000	0.000	-2.178	0.000
CNR2	0.000	0.000	0.000	0.000	0.000	0.000	-3.951	0.000
PDX1	0.000	0.000	0.000	3.948	0.000	0.000	0.000	0.000
GOT2	0.000	1.466	0.000	0.000	0.000	0.000	0.000	0.000
ILK	-2.138	0.000	0.000	0.000	0.000	0.000	0.000	0.000
BNIP3	0.000	0.000	1.175	1.525	0.843	2.234	0.000	1.327
TJP3	6.300	1.338	0.000	3.640	0.000	2.087	0.000	1.456
GSTM1	0.000	0.000	0.000	4.367	0.000	6.263	0.000	0.000
SLC25A5	0.000	0.000	0.000	0.000	0.000	-0.990	0.000	0.000
CMTM2	0.000	2.553	0.000	0.000	0.000	0.000	0.000	0.000
REV1	0.000	0.000	0.000	0.000	0.000	-0.972	0.000	0.000
PIP5K1C	6.316	0.000	0.000	0.000	0.000	0.000	0.000	0.000
TIE1	2.582	0.829	0.000	0.000	0.000	-4.492	0.000	0.000
GABRA6	0.000	3.231	0.000	2.949	0.000	0.000	0.000	0.000

AMOTL2	0.000	-0.706	0.000	-0.656	0.000	-0.745	0.000	0.000
TGS1	2.904	0.000	0.000	0.000	0.000	0.000	0.000	0.000
CCL23	0.000	3.445	0.000	0.000	0.000	0.000	0.000	0.000
IL1RN	0.000	4.500	0.000	0.000	0.000	7.135	0.000	2.849
SDHB	1.895	0.000	0.000	0.000	0.000	0.000	0.000	1.751
FLNA	0.000	0.000	-0.871	-0.698	0.000	0.000	0.000	0.000
TRIP12	1.111	0.000	0.000	1.109	0.000	1.273	0.000	1.290
DEPTOR	0.000	0.000	0.000	3.205	0.000	5.277	0.000	0.000
SPATA2	0.000	0.000	0.000	0.853	0.000	1.886	0.000	0.000
PTPN14	0.000	0.000	-1.218	0.000	0.000	0.000	0.000	0.000
LAMB4	3.340	0.000	1.749	3.369	2.665	2.741	0.000	2.092
COL18A1	0.000	0.000	0.000	0.000	0.000	1.437	0.000	-0.837
TERF2IP	0.000	0.000	0.000	0.000	0.799	0.000	0.000	0.000
THBS2	0.000	0.000	0.000	0.000	0.000	0.000	-0.701	0.000
GRIA1	4.766	2.622	0.000	3.731	0.000	0.000	0.000	3.373
CUL1	0.000	0.000	0.000	0.000	0.596	0.000	0.000	0.000
NOV	0.000	0.000	0.000	0.000	0.000	0.000	0.000	-2.017
SPG7	0.000	0.000	-2.038	-2.498	0.000	0.000	0.000	-1.477
BNIP2	0.000	0.000	0.000	0.000	0.000	-1.937	0.000	0.000
UBA3	0.000	0.000	0.000	0.000	0.000	0.000	0.000	-0.771
GSTP1	0.000	0.000	0.000	0.000	0.000	0.757	0.000	0.000
LPAR3	6.830	0.000	0.000	0.000	0.000	0.000	0.000	0.000
CSNK1G1	0.000	0.000	0.000	0.000	0.000	2.186	0.000	0.879
WNT5A	0.000	0.000	0.000	0.000	0.000	0.000	0.000	-0.713
GRIN3A	0.000	4.397	0.000	4.396	0.000	0.000	0.000	0.000
DCTN2	1.154	0.000	0.000	0.000	0.000	0.000	0.000	0.000
ENO4	0.000	0.000	0.000	2.740	0.000	0.000	0.000	0.000
TGFBR3	0.000	0.000	0.000	0.000	0.000	-4.426	0.000	0.000
DLX5	0.000	0.000	3.137	0.000	0.000	0.000	0.000	0.000
LEP	5.566	0.000	0.000	2.997	0.000	3.098	0.000	0.000
ANAPC4	-2.159	0.000	-0.647	0.000	0.000	0.000	0.000	0.000
GRIN2A	0.000	0.000	0.000	0.000	0.000	0.000	-3.811	0.000
ANGPTL4	0.000	0.000	0.000	1.503	0.000	0.000	0.000	0.000
SPINK5	2.714	1.688	1.298	2.947	2.582	3.832	-2.622	4.271
PSEN2	0.000	0.000	0.000	0.000	0.000	0.000	0.000	2.069
ID1	-2.233	0.000	-0.924	0.000	0.000	0.000	0.000	0.585
ATP1A4	0.000	0.000	0.000	0.000	0.000	5.629	0.000	0.000
RALB	-2.165	0.000	0.000	0.000	0.000	-1.410	0.000	0.000
RHOJ	2.439	1.698	1.302	1.967	1.004	3.709	-2.161	2.017
PLCD3	-2.181	0.000	0.000	0.000	0.000	-3.013	0.000	0.000
UBE2D4	-5.728	-0.752	0.000	0.000	0.000	0.000	0.000	0.000
PDGFRB	0.000	0.000	0.000	0.000	0.000	0.000	-4.559	0.000
CXADR	5.022	0.000	0.000	0.000	0.000	0.000	0.000	0.000

CHRNA4	3.741	0.000	1.929	2.522	0.000	0.000	0.000	2.321
GFAP	2.997	0.000	0.000	3.367	0.000	4.163	0.000	3.197
PLA2G10	0.000	1.497	1.732	2.917	1.807	4.358	-3.030	3.848
ENO1	-0.862	0.000	0.000	0.000	0.000	0.000	0.000	0.000
SGK1	1.849	0.000	0.000	0.000	0.000	0.000	0.000	0.000
LAMP2	-2.884	0.000	0.000	0.000	-0.704	0.000	0.000	0.000
UBASH3B	0.000	0.000	0.000	0.000	0.000	1.536	0.000	0.000
G6PD	1.093	0.000	0.000	0.000	0.000	0.000	0.000	0.000
FADS1	1.745	0.000	0.000	0.000	0.000	0.000	0.000	0.000
GALNT7	2.320	0.000	0.000	0.000	0.000	0.000	0.000	0.000
PIAS1	2.724	0.000	0.000	0.000	0.000	2.883	0.000	0.000
GABARAPL1	0.000	0.000	1.025	0.000	0.000	0.000	0.000	0.000
MAPK11	0.000	0.000	0.000	0.000	-3.406	0.000	0.000	0.000
CD24	0.000	-2.530	0.000	0.000	0.000	0.000	0.000	0.000
GBX2	0.000	0.000	0.000	1.278	0.000	0.000	-1.488	0.000
RASSF1	0.000	-0.685	0.734	0.000	0.000	0.000	0.000	0.000
LPIN1	0.000	0.000	0.000	0.000	0.000	2.220	0.000	0.000
INHBB	0.000	0.000	0.000	0.000	0.000	0.000	0.000	-4.778
FBXO38	0.000	0.000	0.000	0.000	0.000	-1.920	0.000	0.000
CDR2	0.000	0.000	0.000	0.000	0.000	1.351	0.000	0.000
LMNA	1.959	0.593	0.000	1.335	0.000	2.735	0.000	1.592
SMURF2	-2.147	0.000	-0.740	0.000	0.000	-1.445	0.000	0.000
MMP1	0.000	0.000	0.000	1.773	0.000	0.000	2.112	0.000
BDH2	0.000	0.000	0.000	0.000	0.000	0.000	0.000	-1.220
HSPB7	4.103	0.000	0.000	2.974	0.000	5.650	0.000	2.868
VEGFB	0.000	0.000	0.000	0.000	0.000	-1.450	0.000	0.000
NOG	0.000	0.000	0.000	0.000	0.000	0.000	-0.741	0.000
CDKN2C	1.068	0.000	0.000	0.000	0.000	0.000	0.000	0.000
MED1	0.000	0.000	1.119	0.000	0.000	0.000	0.000	0.000
NLRP1	3.303	0.000	0.000	1.696	0.000	0.000	0.000	0.000
NTRK2	6.757	0.000	0.000	0.000	0.000	0.000	0.000	0.000
HCLS1	0.000	1.837	0.000	2.771	1.880	4.274	-2.288	3.888
HK2	1.691	0.000	0.000	0.000	0.000	0.000	0.000	0.000
EIF4EBP1	0.000	0.000	0.000	0.723	0.000	-2.188	0.000	0.000
RAP1B	0.000	0.000	0.000	-3.472	0.000	1.921	0.000	0.000
CYP51A1	0.000	-1.231	0.000	-1.596	0.000	0.000	0.000	0.000
PPARG	0.000	3.094	0.000	2.857	3.728	0.000	0.000	0.000
DAPK1	3.463	1.061	1.254	2.832	0.000	2.368	-1.782	2.690
SIRT2	2.007	0.000	0.000	1.748	0.000	3.128	0.000	0.000
GRM1	5.376	0.000	0.000	2.458	0.000	4.195	0.000	0.000
PRC1	0.000	0.000	0.000	0.000	0.000	0.000	0.000	1.559
SLC25A6	0.000	0.000	0.000	-1.931	0.000	0.000	0.000	0.000
MAP3K7	0.000	0.000	0.000	0.000	0.000	-1.508	0.000	0.000

CEACAM3	4.964	0.000	0.000	2.792	0.000	0.000	0.000	2.634
BNIP3L	0.000	0.000	0.797	0.000	0.000	-4.618	0.000	0.000
TSC22D1	2.227	0.000	1.390	2.760	1.923	3.767	0.000	3.410
TRPM4	4.888	0.000	0.000	3.271	0.000	0.000	0.000	0.000
RGS2	0.000	0.000	0.000	0.000	0.000	3.508	0.000	0.000
CLIC5	0.000	0.000	0.000	4.208	0.000	0.000	0.000	0.000
SOAT1	0.000	0.000	0.000	0.000	0.000	-4.836	0.000	0.000
PLAUR	0.000	0.000	0.000	0.000	0.000	0.000	-2.636	0.000
ISL1	0.000	0.000	0.000	2.718	0.000	0.000	0.000	2.287
CBS	0.000	0.000	0.000	0.000	1.403	0.000	0.000	0.000
LLGL2	5.567	0.000	0.000	0.000	0.000	0.000	0.000	0.000
GTSE1	0.000	0.000	0.794	0.000	0.000	0.000	0.000	0.000
TCF20	1.672	0.000	0.000	0.000	0.000	0.000	0.000	0.000
RFXAP	0.000	0.000	0.000	0.000	0.000	-1.295	0.000	0.000
TTC9	0.000	-0.788	0.000	0.000	0.000	0.000	0.000	0.000
TFDP2	0.000	0.000	0.000	0.000	0.000	0.884	0.000	0.000
MFGE8	0.000	0.000	0.000	0.000	0.000	1.595	0.000	0.773
SH2D1A	0.000	0.000	0.000	0.000	0.000	6.756	0.000	0.000
CMTM3	0.000	0.000	0.000	0.000	0.000	0.000	-3.907	0.000
UBA7	0.000	3.611	0.000	0.000	0.000	5.145	0.000	0.000
ACVR1B	2.215	0.000	0.000	1.225	0.000	1.555	0.000	0.000
RPA1	0.000	0.000	0.000	0.000	0.000	1.065	0.000	0.000
CALB1	2.845	1.352	1.782	3.013	2.061	4.224	-1.277	3.584
MPO	7.071	0.000	0.000	0.000	0.000	0.000	0.000	0.000
LAMB1	0.000	0.000	0.000	1.778	0.000	1.871	0.000	0.000
GRIK4	0.000	0.000	0.000	0.000	0.000	6.344	0.000	0.000
E2F3	3.117	0.000	0.000	0.000	0.000	0.000	0.000	0.000
SLCO1B1	0.000	0.000	0.000	2.758	0.000	5.155	0.000	0.000
WTIP	0.000	0.000	0.000	0.000	-1.278	0.000	0.000	0.000
MAP2K5	0.000	0.000	0.000	0.000	-4.203	0.000	0.000	0.000
SKP1	0.735	0.000	0.000	0.000	0.000	0.000	0.000	0.000
CACNG4	0.000	0.000	0.000	0.000	0.000	0.000	0.000	-0.685
EXOC7	1.689	0.000	0.000	0.000	0.000	0.000	0.000	0.000
GLUD1	-4.585	0.000	0.000	0.000	0.000	0.000	0.000	0.000
UBE2A	-1.186	0.000	0.000	0.000	0.000	0.921	0.000	0.000
TP53INP1	0.000	0.000	0.000	0.000	-2.297	0.000	0.000	0.000
CDH2	0.000	0.000	0.000	0.000	0.000	-0.938	0.000	0.000
CEACAM1	0.000	0.000	0.000	0.000	0.000	6.513	0.000	0.000
UQCRC1	-5.033	0.000	-0.642	0.000	0.000	0.000	0.000	0.000
CHRM2	0.000	2.410	0.000	3.057	0.000	0.000	0.000	3.296
PRKD2	2.748	1.370	1.186	2.418	0.701	2.519	0.000	0.000
ACVR1C	0.000	0.000	0.000	2.924	0.000	0.000	0.000	0.000
PPP3CB	0.000	0.000	0.000	0.000	0.000	-0.659	0.000	0.000

GNB3	0.000	0.000	-2.094	0.000	0.000	0.000	0.000	0.000
TPCN2	2.051	0.000	0.000	0.000	0.000	0.000	0.000	0.000
DBT	0.000	0.000	0.000	0.758	0.000	0.000	0.000	0.000
IL27RA	0.000	2.082	0.000	0.000	0.000	0.000	0.000	0.000
EFNA1	0.000	4.093	0.000	0.000	0.000	0.000	0.000	0.000
NKX3-1	0.000	0.000	0.000	0.000	0.000	3.798	0.000	0.000
HGS	-2.512	0.000	0.000	0.000	0.000	0.000	0.000	0.000
GHRL	0.000	3.951	0.000	1.940	0.000	0.000	-2.271	0.000
SIX1	0.000	0.000	0.000	0.000	0.000	0.000	-0.952	0.000
TTC1	0.000	0.000	0.000	0.000	-1.037	0.000	0.000	0.000
HMOX1	4.165	1.501	0.000	0.932	1.055	2.337	1.453	1.197
DTX3L	0.000	0.000	0.000	0.000	0.000	0.000	-3.812	0.000
HSPA4	0.976	0.000	0.798	1.706	0.000	2.696	0.000	1.606
VASP	1.931	0.000	0.000	0.000	0.000	0.000	0.000	0.000
ACTB	0.000	0.000	-0.585	0.000	0.000	0.000	0.000	0.000
TERF2	-3.335	0.000	0.000	0.000	0.000	-1.890	0.000	0.000
ITGB6	6.710	0.000	0.000	0.000	0.000	0.000	0.000	0.000
ATP6AP2	0.000	0.000	0.000	0.000	0.000	-1.249	0.000	0.000
PLIN4	0.000	0.000	2.026	3.173	0.000	0.000	0.000	0.000
COX4I1	0.000	0.000	0.000	0.000	-0.766	0.000	0.000	0.000
PAK5	-0.622	0.000	0.000	0.000	0.000	-0.624	0.000	0.000
TSPO	0.000	0.000	0.000	0.000	0.000	4.905	0.000	0.000
OGDH	0.000	0.000	-1.089	0.000	0.000	0.000	0.000	0.000
TSLP	0.000	0.000	0.000	3.314	2.205	2.349	0.000	3.386
STMN1	0.000	0.000	0.000	-0.630	0.000	-1.119	0.000	0.000
AXIN1	1.546	0.000	0.000	0.000	0.000	0.000	0.000	0.000
APOL1	0.000	0.000	0.000	0.000	2.759	0.000	0.000	0.000
TFDP1	-1.668	0.000	0.000	0.000	0.000	0.000	0.000	0.000
FGF21	0.000	0.000	0.000	0.000	0.000	4.694	0.000	0.000
FGF4	0.000	4.319	0.000	0.000	0.000	0.000	0.000	0.000
CALD1	0.000	0.000	0.000	-0.722	0.000	-2.055	-0.986	0.000
CCL11	0.000	4.919	0.000	0.000	0.000	6.940	0.000	0.000
LMNB2	0.000	0.000	0.000	0.000	0.000	1.342	0.000	0.000
GRID1	0.000	3.778	0.000	4.032	0.000	0.000	0.000	0.000
SERPINB2	0.000	0.591	0.000	1.432	0.787	1.108	2.212	0.690
ACADM	3.079	0.000	1.160	2.563	1.922	2.803	0.000	2.541
VGLL4	0.000	1.312	0.000	0.000	0.000	0.000	0.000	0.000
FABP3	0.000	0.000	0.000	3.382	0.000	0.000	0.000	0.000
PRKCQ	0.000	0.000	0.000	2.569	0.000	4.026	0.000	0.000
FYN	-5.878	0.000	0.000	0.000	0.000	-2.333	0.000	0.000
APC	0.000	0.000	0.000	0.000	1.065	0.000	0.000	0.000
TSNAX	1.438	0.922	0.754	1.525	0.000	1.381	0.000	1.679
C2	3.984	0.000	0.000	0.000	0.000	2.648	0.000	0.000

ITGB3	0.000	0.000	-1.681	0.000	0.000	0.000	0.000	0.000
CIB1	2.448	0.000	0.000	0.000	0.000	0.000	0.000	0.000
CYB5R3	0.000	0.000	0.000	0.000	0.000	0.000	-0.728	0.000
JAG2	0.000	0.000	0.000	3.979	2.525	3.531	0.000	2.708
MAN2A1	-4.553	0.000	0.000	0.000	0.000	-4.433	0.000	0.000
IFIT3	0.000	0.000	0.000	0.000	0.000	5.929	0.000	0.000
CD44	0.000	0.000	-2.275	0.000	0.000	0.000	0.000	0.000
MAPK6	-2.970	0.000	0.000	0.000	0.000	0.000	0.000	0.000
NHLRC1	0.000	0.000	0.000	0.000	-4.153	0.000	0.000	0.000
PTGER2	0.000	0.000	2.330	3.589	0.000	0.000	0.000	2.058
SQSTM1	0.000	0.000	0.000	0.000	0.000	0.000	1.233	0.000
P2RY6	0.000	0.000	0.000	2.401	0.000	0.000	0.000	0.000
HLA-DMA	0.000	2.241	0.000	2.262	0.000	0.000	0.000	0.000
TXLNA	1.057	0.000	0.000	0.000	0.000	-2.526	0.000	0.000
LRPAP1	0.000	0.000	0.000	0.000	0.000	1.898	0.000	0.000
PCNA	0.000	0.000	0.000	0.000	0.000	-1.356	0.000	0.000
CCL26	0.000	0.000	0.000	0.000	0.000	2.660	0.000	0.000
ASPH	0.000	1.558	0.000	2.605	1.991	4.765	0.000	2.851
PARVB	0.000	0.000	-0.691	0.000	0.000	0.000	0.000	0.000
KIF5B	0.000	0.000	0.000	0.000	0.000	-1.121	0.000	0.000
SCT	5.708	0.000	0.000	0.000	0.000	0.000	0.000	0.000
LOXL1	2.752	0.000	0.000	0.000	0.000	0.000	0.000	0.000
ADORA2B	2.977	0.000	0.000	0.000	0.000	0.000	0.000	0.000
NFKBIB	0.000	0.000	0.000	0.000	0.000	4.731	0.000	0.000
FXYD2	7.379	0.000	0.000	0.000	0.000	0.000	0.000	0.000
WT1	0.000	2.311	0.000	0.000	0.000	0.000	0.000	0.000
UBE2R2	0.000	0.000	0.000	0.000	0.000	2.680	0.000	0.000
KRT18	1.584	1.219	0.000	0.000	0.000	2.562	0.000	0.000
PPP2R2A	0.000	0.000	0.000	0.000	0.000	-2.105	0.000	0.000
POU2F1	0.000	0.000	0.000	1.868	0.000	3.640	0.000	1.535
WNT2B	0.000	0.000	0.000	3.188	0.000	3.696	0.000	-3.882
TIMP3	-6.029	0.000	0.000	0.000	0.667	1.100	0.000	0.000
ZFP36	0.000	0.000	0.000	0.000	0.000	0.000	-0.895	0.000
MMS19	0.000	0.000	0.000	0.000	0.000	0.000	0.000	-3.275
AGAP2	0.000	0.000	0.000	0.000	0.000	6.872	0.000	0.000
ABCC3	2.346	1.508	0.000	2.342	0.000	0.000	0.000	0.000
WARS	1.978	1.162	1.443	2.545	1.501	3.213	-1.118	3.094
IL2RB	0.000	0.000	0.000	1.915	0.000	4.843	0.000	0.000
SUMO1	-1.466	0.000	0.000	0.000	0.000	0.000	0.000	0.000
FRS2	0.000	0.000	0.000	0.000	0.000	0.000	0.000	-1.034
BHLHE40	0.000	0.000	0.000	1.805	0.000	3.282	0.000	3.465
CDC42	0.000	0.000	0.000	0.000	0.000	-1.099	0.000	0.000
UQCRFS1	1.971	0.000	0.000	0.000	0.000	3.694	0.000	0.000

CD1A	3.885	0.000	0.000	3.129	0.000	0.000	0.000	0.000
STAB1	0.000	4.275	0.000	0.000	0.000	0.000	0.000	0.000
HOXD4	0.000	0.000	0.000	3.569	0.000	0.000	0.000	0.000
RERE	0.000	0.000	0.000	0.000	0.000	0.831	0.000	0.000
GK	0.000	0.000	0.000	0.000	0.000	0.000	0.688	0.000
PIAS3	0.000	0.000	0.598	0.000	0.000	0.000	0.000	0.000
PRKAG2	0.000	0.000	0.000	0.000	-1.299	0.000	0.000	0.000
CLDN11	0.000	0.000	-0.697	0.000	0.000	-0.879	0.000	0.000
APOC2	6.389	0.000	0.000	2.592	0.000	0.000	0.000	0.000
DUSP8	3.259	1.911	1.061	2.638	1.989	3.538	-1.109	2.906
COL5A1	0.000	0.000	-0.901	0.000	0.000	0.000	-0.593	0.000
CSNK2A1	3.042	0.000	0.000	0.000	0.000	0.000	0.000	0.000
FER	0.000	0.000	0.000	0.000	0.000	2.191	0.000	1.742
RASA1	0.000	0.000	0.000	-2.147	0.000	0.000	0.000	-1.264
CCL20	0.000	3.094	0.000	0.000	0.000	0.000	0.000	0.000
PPP2R3C	-1.177	0.000	0.000	0.000	0.000	0.839	0.000	0.000
AKT3	0.000	0.000	0.000	0.000	0.000	-3.190	0.000	0.000
CHSY1	0.000	0.000	-1.797	0.000	0.000	0.000	0.000	0.000
MKI67	-0.841	0.000	0.000	0.000	0.000	0.000	0.000	0.000
LDHA	3.116	0.000	1.317	2.878	0.000	2.339	0.000	1.529
PTPN6	0.000	1.953	1.342	2.268	1.480	2.562	-2.641	2.846
VEGFA	0.000	0.808	0.798	2.232	1.667	3.431	0.000	3.037
FGF11	0.000	0.000	0.000	2.684	0.000	0.000	0.000	0.000
ALDOA	-2.635	0.000	0.000	0.000	0.000	0.000	0.000	0.000
SLC27A4	2.302	0.000	1.493	2.821	1.845	3.066	-1.468	3.627
CLCA1	0.000	0.000	0.000	3.758	0.000	0.000	0.000	0.000
FOXO1	0.000	0.000	0.000	2.344	0.000	0.000	0.000	0.000
NPY	0.000	0.000	0.000	0.000	0.000	7.753	0.000	0.000
GBP5	3.610	0.000	0.000	0.000	0.000	3.325	0.000	0.000
CHRNA5	0.000	0.000	0.000	0.000	0.000	0.000	0.000	1.189
ACY1	0.000	1.202	0.000	1.666	0.000	0.000	0.000	0.000
PRKACA	0.000	0.000	0.000	0.000	0.000	-2.706	0.000	0.000
ATG3	0.000	0.000	0.000	0.000	0.000	-0.959	0.000	0.000
NAP1L1	0.000	0.000	0.000	0.000	0.000	0.608	0.000	0.000
TXNRD1	0.000	0.911	0.946	0.000	0.000	0.000	1.263	0.000
PDE10A	0.000	0.000	0.000	2.455	0.000	0.000	0.000	0.000
EPHB4	5.884	0.000	0.000	0.000	0.000	0.000	0.000	0.000
RASA3	-4.704	0.000	-0.905	0.000	0.000	1.206	0.000	0.000
ATP6V1A	2.039	0.000	0.733	1.687	0.000	1.545	0.000	1.314
OCLN	0.000	0.000	0.000	1.097	0.000	2.064	0.000	0.000
SLCO1B7	0.000	0.000	0.000	3.869	0.000	0.000	0.000	0.000
ARF6	0.000	0.000	-1.088	0.000	0.000	0.000	0.000	0.000
CTNND1	0.000	0.000	0.000	0.000	0.000	-1.679	0.000	0.000

FUCA1	-1.145	0.000	0.000	0.000	0.000	0.000	0.000	0.000
BTRC	0.000	0.876	1.093	1.784	0.000	1.673	0.000	0.000
CD47	1.666	2.037	0.000	2.135	1.233	1.781	0.000	0.000
E2F1	0.000	0.000	0.691	0.000	0.000	1.069	0.000	0.000
HDLBP	-4.992	0.000	-0.887	-0.930	0.000	0.000	0.000	0.000
HTR1D	0.000	1.605	0.000	3.102	2.472	2.866	-2.168	1.877
NPM1	0.000	0.000	0.000	0.000	0.000	0.752	0.000	0.000
ACVR2A	0.000	1.049	0.000	1.897	1.231	4.109	0.000	3.013
MYH10	0.000	0.000	0.000	0.000	0.000	-1.923	0.000	0.000
CPE	0.000	1.409	0.000	0.000	0.000	0.000	0.000	0.000
CD1C	4.037	2.457	1.688	3.465	2.052	0.000	-3.818	3.165
MARK2	3.957	0.000	0.000	0.000	0.000	0.000	0.000	2.689
EBI3	0.000	0.000	0.000	0.000	0.000	5.713	0.000	0.000
NRP2	3.702	0.000	0.000	1.450	0.000	0.000	0.000	0.000
TRPV2	0.000	0.000	-2.661	0.000	0.000	0.000	0.000	0.000
STX4	0.000	0.000	0.000	0.000	-4.076	0.000	0.000	0.000
NEK7	0.000	0.000	0.000	-1.054	0.000	-1.334	0.000	0.000
FGB	0.000	0.000	0.000	2.267	0.000	0.000	0.000	0.000
TIMP1	0.000	0.000	0.000	0.000	0.000	1.054	0.000	0.000
UBE2E2	0.000	0.000	0.000	0.000	0.000	-4.673	0.000	0.000
BCL9	0.000	2.028	0.000	0.000	0.000	4.036	0.000	0.000
PTGFR	0.000	1.403	1.227	1.998	1.266	2.277	-1.598	0.000
FZD2	0.000	0.000	0.000	-0.734	0.000	0.000	0.000	-0.759
ELOB	0.000	0.000	-0.592	0.000	0.000	0.000	0.000	0.000
IDH3A	0.000	0.000	0.000	0.000	0.000	-5.296	0.000	0.000
ERCC8	0.000	0.000	0.000	0.000	0.000	3.684	0.000	0.000
APP	0.000	0.000	0.000	0.000	0.000	-3.179	0.000	0.000
NDUFB8	0.000	2.891	0.000	3.742	0.000	0.000	-3.753	2.756
GABBR1	0.000	0.000	0.000	-0.991	0.000	0.000	0.000	0.000
SLC22A1	0.000	0.000	0.000	2.843	2.357	4.578	0.000	1.751
PDPR	0.000	0.000	0.000	0.000	0.000	0.000	0.000	-0.759
CACNA2D1	3.022	2.483	0.000	2.481	0.000	0.000	0.000	0.000
LPAR4	0.000	0.000	0.000	2.477	1.547	4.030	0.000	3.998
CHMP2A	0.000	0.000	0.000	0.000	0.000	0.000	0.000	1.270
HDAC2	0.000	0.000	-0.689	0.000	0.000	0.000	0.000	0.000
MCM3	0.000	0.000	0.000	0.000	0.000	-4.552	0.000	0.000
NRG1	0.000	0.000	0.000	-1.527	0.000	0.000	0.000	0.000
TLR1	0.000	0.000	0.000	2.047	0.000	0.000	0.000	0.000
TJP1	0.000	0.000	0.000	0.000	0.000	-1.353	0.000	0.000
PDE4B	0.000	0.000	0.000	0.000	2.934	0.000	0.000	0.000
EFNA3	0.000	3.087	0.000	0.000	0.000	5.732	0.000	0.000
COX3	3.142	0.000	0.000	0.000	0.000	3.133	0.000	0.000
RFNG	4.783	0.000	0.000	2.145	0.805	0.000	0.000	0.917

NEDD4	0.000	0.000	0.000	0.000	0.000	-1.170	0.000	0.000
DEPDC5	1.527	0.000	0.000	0.000	0.000	0.000	0.000	0.000
WNT7B	0.000	2.722	0.000	0.000	0.000	4.460	0.000	0.000
HERPUD1	0.000	0.000	0.690	0.000	0.000	0.000	0.000	0.000
STRADA	1.855	1.236	0.000	1.761	0.000	2.200	-0.888	2.417
SPATA20	0.000	0.000	0.000	2.397	0.000	0.000	0.000	0.000
SMAD3	0.000	0.000	0.000	1.472	0.000	0.000	0.000	0.000
IL6	0.000	0.000	0.000	0.000	0.000	4.025	0.000	0.000
TICAM1	0.000	0.000	0.000	0.000	0.000	0.000	0.000	2.885
RARB	0.000	0.000	0.000	0.000	0.000	0.000	-3.580	0.000
PTGER3	4.795	2.698	2.628	3.854	0.000	3.422	-3.455	1.769
CCNB3	4.855	0.000	0.000	3.722	0.000	0.000	0.000	0.000
PTX3	0.968	0.000	-0.897	0.589	0.000	0.000	0.000	1.038
PCGF2	3.084	0.000	0.000	0.000	0.000	0.000	0.000	0.000
UBOX5	0.000	0.000	0.000	0.000	0.000	-3.302	0.000	0.000
ADORA2A	0.000	4.718	0.000	0.000	0.000	0.000	0.000	0.000
GNA12	0.000	0.000	0.000	0.000	0.000	-4.238	0.000	0.000
RPS6KA6	-4.173	0.000	0.000	-1.344	-0.955	0.000	0.000	0.000
ENPEP	4.189	0.000	0.000	4.487	0.000	0.000	0.000	3.119
FGF9	0.000	0.000	0.000	2.806	0.000	0.000	0.000	0.000
AGL	3.767	0.000	0.000	0.000	0.000	0.000	-1.627	0.000
ACTN1	0.000	0.000	-0.592	0.000	0.000	0.000	0.000	0.000
MICB	2.039	0.000	1.272	2.037	0.000	0.000	0.000	2.240
CCL13	0.000	3.626	0.000	0.000	0.000	0.000	0.000	0.000
MAP1LC3A	0.000	0.000	0.000	0.000	0.000	2.018	0.000	0.000
TNR	0.000	0.000	1.942	3.379	0.000	4.066	-3.873	3.057
SLC9A3	0.000	0.000	2.067	3.736	2.625	3.076	0.000	2.592
VAMP2	0.000	1.286	0.000	0.000	0.000	0.000	0.000	0.000
CD8B	0.000	5.085	0.000	0.000	0.000	0.000	0.000	0.000
MYD88	0.000	2.566	0.000	2.007	0.000	3.432	0.000	0.000
ATP1B3	-3.423	0.000	0.000	0.000	0.000	0.000	0.000	0.000
RNASEL	1.525	0.000	0.000	1.522	0.825	0.000	0.000	0.000
ERCC3	0.000	0.000	0.000	0.000	0.000	0.000	0.000	-1.404
CDKN1B	1.324	0.000	0.000	-1.077	0.000	0.000	0.000	0.000
LIPC	0.000	0.000	0.000	0.000	-4.177	0.000	0.000	0.000
POMGNT1	-4.256	0.000	0.000	0.000	0.000	0.000	0.000	0.000
IRS2	0.000	0.000	0.000	0.000	0.000	1.306	-0.887	0.000
DTX4	3.581	0.000	0.000	2.372	0.000	0.000	0.000	1.767
TICAM2	0.000	0.000	0.000	1.164	0.000	2.588	0.000	0.000
CDK8	0.000	2.528	0.000	0.000	0.000	0.000	0.000	2.193
XIAP	-3.847	0.000	0.000	0.000	0.000	-1.805	0.000	0.000
PPP2R5E	0.000	0.000	0.000	1.121	0.000	0.000	0.000	0.000
ROCK2	-1.694	0.000	0.000	0.000	0.000	-2.663	0.000	0.000

NGFR	0.000	0.000	0.000	0.000	-4.048	0.000	0.000	0.000
NBN	0.000	0.000	0.677	0.000	0.000	0.000	0.000	0.000
DDX3X	0.000	0.000	0.000	0.000	-3.764	0.000	0.000	0.000
SERPINE1	0.000	0.000	-1.211	0.000	0.000	-0.805	0.000	0.601
UQCRB	0.000	-1.735	0.000	0.000	0.000	0.000	0.000	0.000
CAMSAP1	0.000	0.913	0.000	1.505	0.000	1.721	0.000	2.095
CKLF	0.000	0.000	0.000	0.000	0.000	1.362	0.000	0.000
NDUFA2	0.000	0.000	0.000	0.000	-0.829	0.000	0.000	0.000
CTSW	0.000	1.595	0.000	3.292	1.826	0.000	-2.784	2.422
PVRL2	0.000	0.000	0.000	2.757	2.193	2.992	0.000	3.502
NOSTRIN	4.160	0.000	2.077	3.255	0.000	2.439	-2.007	3.469
FLG	0.000	1.914	0.000	2.718	0.000	0.000	0.000	1.628
IFNAR1	0.000	0.000	-0.658	0.000	0.000	0.000	0.000	0.000
GSR	0.000	0.000	0.000	0.000	0.000	-1.080	0.682	0.000
ARID5B	0.000	-0.643	0.000	0.000	0.000	-1.714	-0.696	0.000
GPC1	0.000	0.000	0.000	0.000	0.000	0.000	0.000	-0.687
CAV1	0.000	0.000	0.000	0.000	0.000	-0.993	0.000	0.000
ATP1A1	0.953	0.000	0.000	0.000	0.000	0.000	0.000	0.000
TNFRSF10B	4.464	0.000	0.000	0.000	0.000	0.000	0.000	0.000
KIF2C	0.000	0.000	1.230	0.000	0.000	0.000	0.000	0.000
FLT3	0.000	0.000	0.000	4.619	0.000	0.000	0.000	0.000
STIM1	3.943	0.000	1.531	0.000	0.000	0.000	0.000	0.000
XPA	0.000	0.000	0.000	0.000	0.000	3.608	0.000	0.000
LFNG	0.000	1.928	0.000	1.735	1.887	0.000	0.000	0.000
TAOK1	-1.128	0.000	0.000	0.000	0.000	0.000	0.000	0.000
CHEK2	0.000	1.984	0.000	0.000	0.000	3.049	0.000	2.431
CREG1	-2.120	0.000	0.000	0.000	0.000	0.000	0.000	0.000
CHRNE	0.000	2.713	0.000	0.000	0.000	0.000	0.000	0.000
GNB5	-4.864	0.619	0.000	0.000	0.000	0.000	0.000	-0.732
RAB10	0.000	0.000	0.000	0.000	0.000	-1.367	0.000	0.000
NPR2	0.000	0.000	0.000	1.251	0.000	0.000	0.000	0.000
PARP3	0.000	0.000	0.000	0.000	0.000	0.000	0.000	-0.687
CYLD	0.000	0.000	0.000	0.000	0.000	2.762	0.000	0.000
AJUBA	1.228	0.000	0.000	0.000	0.000	0.000	0.000	-0.976
ANGPT2	0.000	0.000	0.000	3.993	0.000	6.939	0.000	0.000
IL17RC	0.000	0.000	0.000	0.000	0.000	2.556	0.000	0.000
MT1E	0.000	0.000	-2.101	0.000	0.000	0.000	0.000	0.000
SOS2	0.000	0.000	0.000	0.000	0.000	-1.593	0.000	0.000
ACOX1	1.413	0.000	0.000	0.000	0.000	0.000	0.633	0.000
SLC11A1	0.000	0.000	0.000	4.620	0.000	0.000	0.000	0.000
MRAS	-4.461	0.000	-0.722	0.000	0.000	0.000	0.000	0.000
BMP2	3.747	1.257	1.762	3.147	1.178	3.598	0.000	2.764
CREM	0.000	0.000	-2.419	0.000	0.000	0.000	0.000	0.000

APBB1IP	3.734	1.635	0.000	2.809	2.862	4.645	-4.641	2.942
PDPK1	0.000	1.794	0.000	0.000	0.000	0.000	0.000	0.000
NDUFA3	-0.744	0.000	0.000	0.000	0.000	0.000	0.000	0.000
MAP2K6	0.000	0.000	0.000	4.222	0.000	0.000	0.000	0.000
BIRC8	0.000	3.585	0.000	4.315	0.000	5.494	0.000	2.650
TNKS2	0.000	0.000	0.000	0.000	0.000	2.042	0.000	0.000
GRIA2	3.444	2.185	1.687	3.489	2.863	2.874	-2.642	1.835
WNT5B	0.000	0.000	0.000	-0.610	0.000	-1.962	0.000	0.000
RPS6KA3	0.000	1.380	0.000	1.940	0.000	2.702	0.000	2.800
CSH1	8.464	0.000	0.000	0.000	0.000	0.000	0.000	0.000
TYK2	0.000	0.000	0.000	0.894	0.000	1.780	0.000	0.000
CAMKK2	0.000	0.000	0.000	0.000	0.000	-5.554	0.000	-0.640
MFSD11	0.000	2.521	0.000	0.000	0.000	3.725	0.000	0.000
RAD21	0.000	2.581	0.000	3.315	2.527	0.000	0.000	1.906
PECR	0.000	0.000	0.000	0.000	0.000	0.000	0.000	0.901
DGKZ	0.000	0.000	0.000	0.000	0.000	2.116	0.000	0.000
LRP10	0.000	0.000	0.000	0.000	0.000	-1.755	0.000	0.000
FXYD5	0.000	0.000	-1.033	0.000	0.000	0.000	0.000	0.000
STK3	2.537	0.000	0.000	0.000	0.000	0.000	0.000	0.000
UBE2E3	-4.414	0.000	0.000	0.000	0.000	-4.320	0.000	0.000
CFI	2.615	0.000	0.000	2.833	0.000	2.929	-1.856	2.486
CNBP	4.142	2.175	0.000	0.000	0.000	0.000	0.000	0.000
HDAC1	2.551	0.000	0.000	0.000	-1.220	0.000	0.000	0.000
XRCC2	0.000	0.000	0.000	-1.248	0.000	0.000	0.000	0.000
LAT2	5.240	0.000	0.000	0.000	0.000	0.000	0.000	0.000
ATG9A	0.000	0.000	0.000	0.000	0.000	0.000	0.000	-3.238
CLTC	0.000	0.000	0.000	0.000	0.000	-0.771	0.000	0.000
RORC	4.511	0.000	0.000	2.543	0.000	4.056	0.000	0.000
TGFBR1	0.000	0.000	0.000	0.000	0.000	0.000	0.000	-1.533
ABL1	-2.017	0.000	0.000	0.000	0.000	0.000	0.000	0.000
SHARPIN	0.000	0.000	-1.792	1.254	0.000	0.000	0.000	0.000
HLA-F	0.000	0.000	0.000	0.000	0.000	0.000	0.000	2.878
PIK3R4	0.000	0.000	1.314	2.299	0.000	2.543	0.000	1.748
CPS1	0.000	0.000	0.000	2.984	0.000	0.000	0.000	0.000
GSTA4	0.000	0.000	0.000	2.354	0.000	0.000	0.000	0.000
MAP3K12	0.000	1.493	0.000	0.000	-1.906	0.000	0.000	0.000
TIFA	0.000	0.000	0.000	2.324	0.000	0.000	0.000	0.000
ABHD3	1.915	0.000	0.000	0.000	0.000	0.000	0.000	0.000
MCM5	-1.197	0.000	-0.923	0.000	0.000	-1.081	0.000	0.000
NDUFB6	0.000	0.000	0.000	0.000	0.000	-0.786	0.000	0.000
GNL3	0.000	-1.216	0.000	0.000	0.000	0.000	0.000	0.000
PNKP	2.135	0.000	0.000	0.000	0.000	0.000	0.000	0.000
UGGT2	0.000	0.000	-1.084	0.000	0.000	0.000	0.000	0.000

BAMBI	0.000	0.000	0.000	0.000	0.000	2.189	0.000	0.000
ME1	0.000	0.000	0.000	0.000	0.000	1.076	0.000	0.000
ACTG2	0.000	0.000	-0.731	0.000	0.000	-1.538	0.000	0.000
CXCL5	0.000	0.000	0.000	1.264	0.000	0.000	0.000	0.000
SLC2A4	0.000	0.000	0.000	0.000	0.000	7.770	0.000	0.000
NR4A2	0.000	0.000	0.000	0.000	0.000	4.479	0.000	0.000
WEE1	-1.057	0.000	0.000	0.000	0.000	0.000	0.000	0.000
NDUFB4	1.802	0.000	0.000	0.000	0.000	2.165	0.000	0.000
TNFRSF11B	0.000	0.000	0.000	2.414	0.000	0.000	0.000	0.000
UQCR10	1.781	0.000	0.000	0.000	0.000	0.000	0.000	0.000
IFRD2	0.000	0.000	-0.854	0.000	0.000	0.000	0.000	0.000
RPL19	0.000	0.000	0.000	0.000	0.000	1.201	0.000	0.000
CYTB	0.948	0.000	0.000	0.000	0.000	0.000	0.000	0.000
ALDH1A1	0.000	0.000	0.000	2.715	0.000	0.000	0.000	0.000
GLUD2	0.000	0.000	0.000	0.000	0.000	-1.906	0.000	0.000
TOX	3.060	0.000	0.000	2.011	0.000	0.000	0.000	1.936
LMNB1	0.000	0.000	0.000	0.000	0.000	0.000	0.000	-0.585
CPT1C	3.122	0.000	0.000	1.663	0.000	2.565	0.000	0.000
MTA1	0.000	0.000	0.000	0.000	0.000	-4.383	0.000	0.000
CAMK1G	2.599	2.072	1.352	2.924	0.000	3.776	0.000	4.075
NDUFV1	0.000	0.000	0.000	0.000	0.000	4.839	0.000	0.000
F5	4.908	1.360	2.245	3.641	0.000	4.182	-2.296	2.335
CXCL2	0.000	0.000	0.000	2.350	0.000	0.000	0.000	0.000
TNS1	0.000	0.000	0.000	0.000	0.000	2.172	0.000	0.000
ANKRA2	0.000	0.000	0.606	-1.055	0.000	0.000	0.000	0.000
PGAM4	0.000	0.000	0.000	0.000	0.000	0.000	-3.726	0.000
RASGRP1	0.000	0.000	0.000	0.000	-1.802	0.000	0.000	0.000
APOD	0.000	0.000	2.024	3.841	0.000	4.016	-1.860	3.638
DAD1	2.358	0.000	0.000	0.000	0.000	0.000	0.000	0.000
MCPH1	2.930	0.000	0.000	0.000	0.000	0.000	0.000	0.000
SMAD1	0.000	0.000	0.000	1.303	0.000	0.000	0.000	0.000
GABRB1	0.000	4.500	0.000	0.000	0.000	0.000	0.000	0.000
PRLR	0.000	1.721	0.000	2.271	0.000	2.820	0.000	1.979
IFNG	2.657	2.130	0.000	2.695	2.938	3.021	-3.107	4.589
CXCR2	0.000	0.000	0.000	3.256	0.000	0.000	0.000	0.000
DOCK2	0.000	2.776	0.000	0.000	0.000	0.000	0.000	0.000
HMGB1	1.222	0.000	0.590	1.293	0.000	2.367	0.000	1.399
TIMP2	2.388	0.000	0.000	0.852	0.000	1.682	-0.747	0.000
SFTPD	5.582	0.000	0.000	0.000	0.000	0.000	0.000	0.000
MASP1	3.608	0.000	0.000	3.062	0.000	4.197	0.000	0.000
PRKACB	0.000	0.000	0.000	0.000	0.000	-1.099	0.000	0.000
ULK2	0.000	0.000	0.000	0.000	0.000	7.564	0.000	0.000
IDH3G	0.000	0.000	0.000	-0.656	0.000	0.000	0.000	0.000

GBP2	2.883	0.000	0.000	0.000	0.000	0.000	0.000	-4.181
PTCH1	0.000	0.000	0.000	0.000	0.000	3.464	1.480	0.000
GRB7	4.425	0.000	0.000	2.655	2.336	4.135	0.000	0.000
PTGER1	0.000	0.000	0.000	0.000	0.000	0.000	0.000	3.828
EFNB2	0.000	0.000	0.000	0.000	0.000	2.226	0.000	0.000
PIM1	3.428	2.189	0.000	2.616	0.000	0.000	-2.461	2.835
MT1F	1.813	0.000	-0.905	0.000	0.000	0.000	0.000	0.000
IL22RA1	0.000	0.000	0.000	0.000	0.000	0.000	-4.675	0.000
NEU1	0.000	1.314	0.000	0.000	0.000	0.000	0.000	0.000
GUCY1A2	4.197	0.000	0.000	3.375	0.000	4.876	0.000	2.507
ATRX	0.000	0.000	1.630	3.026	2.019	4.240	-2.549	3.625
TCIRG1	0.000	0.000	0.000	2.597	0.000	4.294	0.000	2.536
TLN2	0.000	0.000	-0.736	0.000	0.000	0.000	-0.940	0.000
GATM	0.000	0.000	0.000	2.957	0.000	0.000	0.000	0.000
RPS6KB1	2.595	0.000	0.000	0.000	0.000	0.000	0.000	0.000
ENAH	0.000	2.939	0.953	2.402	1.973	3.077	-1.113	0.000
FGF19	0.000	0.000	0.000	0.000	0.000	6.547	0.000	0.000
EFNA5	0.000	0.000	0.000	0.000	0.000	-5.033	0.000	0.000
AK2	0.000	0.000	-1.345	0.000	0.000	0.000	0.000	0.000
NTRK1	3.036	0.000	0.000	2.603	0.000	0.000	0.000	2.177
RNASE3	0.000	0.000	0.000	2.611	0.000	0.000	0.000	0.000
ACOT7	0.000	0.000	0.000	0.000	0.000	1.591	0.000	0.593
HLA-DPA1	0.000	2.646	0.000	0.000	0.000	0.000	0.000	0.000
MEIS1	0.000	0.000	0.000	0.000	0.000	2.290	0.000	0.000
MLST8	0.000	0.000	0.000	1.615	0.000	0.000	0.000	0.000
PRSS1	0.000	2.528	0.000	3.341	1.997	3.589	0.000	3.604
NRAS	0.000	0.000	0.000	2.626	0.000	0.000	0.000	0.000
PSTS	4.201	0.000	0.000	3.015	0.000	5.056	0.000	3.235
YWHAZ	0.000	0.000	0.000	0.000	0.000	-1.279	0.000	0.000
TNC	0.000	0.000	0.000	0.000	0.000	0.000	-1.558	0.000
G6PC2	0.000	0.000	2.156	3.230	3.093	4.312	-4.063	3.901
EI24	2.527	0.000	0.000	0.000	0.000	0.000	0.000	0.000
PLAT	3.837	0.000	0.000	0.000	0.000	0.000	0.000	0.000
NSF	0.000	0.000	0.000	0.000	0.000	0.000	-0.959	0.000
ERCC5	0.000	0.000	0.000	0.000	0.000	2.465	0.000	0.000
DUSP14	0.000	0.000	0.000	0.000	0.000	0.000	-0.700	0.000
TRH	0.000	5.274	0.000	0.000	0.000	0.000	0.000	0.000
XRCC4	0.000	0.000	0.000	0.000	0.000	-4.208	0.000	0.000
MMP9	0.000	0.000	0.000	0.000	0.000	4.509	0.000	0.000
MGAT4B	3.114	1.188	1.440	2.890	2.065	3.591	-0.970	3.821
F3	1.763	-1.393	0.000	-2.720	0.000	0.000	-1.105	0.000
EHHADH	0.000	0.000	0.000	2.348	0.000	0.000	0.000	0.000
KCNJ1	0.000	0.000	0.000	2.637	2.546	0.000	0.000	0.000

PABPC1	-0.686	0.000	0.000	0.000	0.000	-0.676	0.000	0.000
MRPL57	0.720	0.000	0.000	0.000	0.000	0.000	0.000	0.000
PPARGC1A	2.963	0.000	1.617	3.143	1.842	3.975	-1.839	4.071
DDB1	0.000	0.000	0.000	0.000	0.000	-3.097	0.000	0.000
PLA2G2D	0.000	0.000	0.000	4.399	0.000	8.564	0.000	0.000
AIRE	6.997	0.000	0.000	4.152	0.000	5.507	0.000	0.000
GH1	0.000	0.000	0.000	4.177	0.000	0.000	0.000	0.000
BLNK	0.000	2.193	0.000	0.000	0.000	0.000	0.000	0.000
F13B	0.000	3.109	0.000	2.741	3.293	0.000	0.000	0.000
GNB4	0.000	0.000	0.000	0.000	0.000	-4.955	0.000	0.000
TNFRSF19	4.175	0.000	0.000	0.000	0.000	0.000	0.000	0.000
OSMR	0.000	0.000	0.000	0.000	0.000	-4.584	0.000	0.000
IL17B	5.964	0.000	0.000	0.000	0.000	0.000	0.000	0.000
ID2	0.000	0.000	0.000	0.000	0.000	2.891	0.000	0.000
FLOT2	3.017	0.000	0.000	2.626	0.000	0.000	0.000	0.000
ADCY5	0.000	0.000	1.909	3.276	1.940	3.713	-2.701	3.145
EGLN1	0.000	0.000	0.000	0.000	-2.572	0.000	0.000	-1.168
CYP4A22	0.000	2.565	2.292	3.735	2.509	0.000	-4.876	2.452
CCND1	0.000	0.000	0.000	0.000	0.000	0.000	0.000	0.861
PSIP1	0.000	0.000	0.000	0.000	0.000	-0.665	0.000	0.000
PA2G4	-4.864	0.000	0.000	0.000	0.000	1.288	0.000	0.000
HMGA1	0.000	0.000	-1.263	0.000	0.000	0.000	0.000	1.187
ERCC1	-4.977	0.000	0.000	0.000	0.000	0.000	0.000	0.000
PRL	0.000	1.770	0.000	2.616	0.000	0.000	0.000	0.000
IL5RA	5.066	3.730	0.000	3.351	0.000	0.000	0.000	3.213
SOD1	0.000	0.000	0.000	0.000	0.000	3.825	0.000	0.000
SLC3A2	0.000	0.000	1.558	0.000	0.000	0.000	0.000	0.000
UBE4B	0.000	0.000	0.000	0.000	0.000	-5.111	0.000	0.000
IFITM3	1.373	0.000	0.000	0.000	0.000	2.022	0.000	0.000
C7ORF50	0.000	0.000	0.000	0.000	0.000	1.293	0.621	0.000
DET1	3.068	1.940	0.000	2.496	1.907	2.540	0.000	0.000
CALCR	7.071	0.000	0.000	0.000	0.000	0.000	0.000	0.000
ACO1	0.000	0.000	0.000	1.092	0.000	0.000	0.000	0.000
NR1H2	0.000	0.000	0.000	0.000	0.000	-4.213	0.000	0.000
PTPRC	0.000	0.000	0.000	0.000	0.000	4.445	0.000	0.000
IFI6	0.000	0.000	0.000	0.000	0.000	1.103	0.000	-0.637
FBXO4	0.000	0.000	0.000	0.000	0.000	0.000	-0.744	1.305
SOS	0.000	0.000	0.000	0.000	0.000	1.552	0.000	-0.828
RAMP1	0.000	0.000	0.000	2.854	0.000	0.000	0.000	3.170
GSK3B	0.000	0.000	0.000	0.000	0.000	-5.483	0.000	0.000
ATF6	0.000	0.000	0.000	0.000	0.000	-4.781	0.000	0.000
DENND4A	0.000	0.000	0.000	0.000	0.000	-4.663	0.000	0.000
NF2	-1.259	0.000	0.000	0.000	0.000	0.000	0.000	0.000

PTK2B	0.000	0.000	1.064	2.835	0.000	2.634	0.000	2.998
PDGFA	-4.187	0.000	-1.101	0.000	0.000	0.000	0.000	0.000
PARP4	-0.712	0.000	0.000	0.000	0.000	0.000	0.000	0.000
CDC25B	0.000	0.000	0.000	-0.626	0.000	0.000	0.000	0.000
BAG1	2.040	0.000	0.000	0.000	0.000	2.182	0.000	0.000
HLA-E	0.000	1.372	0.000	0.000	0.000	0.000	0.000	0.000
ACAD10	0.000	0.000	0.000	0.000	1.791	0.000	0.000	0.000
TJAP1	4.301	0.000	0.000	2.647	0.000	0.000	-4.535	2.089
TPM4	0.000	0.000	0.000	0.000	0.000	0.000	-3.971	0.000
GPR161	3.975	0.000	0.000	2.692	1.408	0.000	0.000	3.113
NMU	0.000	0.000	0.000	0.000	0.000	-4.309	0.000	-0.743
OAT	0.000	0.000	0.000	0.000	1.840	0.000	0.000	3.504
PPAP2B	7.071	0.000	0.000	0.000	0.000	0.000	0.000	0.000
COX5A	-0.958	0.000	0.000	0.000	0.000	0.000	0.000	0.000
ZFP36L1	1.806	0.000	0.000	0.000	0.000	0.000	0.000	0.826
ATG5	-1.670	0.000	0.000	0.000	0.000	0.869	0.000	0.000
COLEC12	0.000	0.000	0.000	0.000	0.000	5.929	0.000	0.000
PARP2	0.000	0.000	0.000	0.000	0.000	0.000	-3.398	0.000
CCL2	0.000	0.000	0.000	1.398	0.000	0.000	0.000	0.000
RBM23	0.000	0.000	0.000	-0.875	0.000	0.000	0.000	0.000
FAM3B	5.655	0.000	0.000	2.474	0.000	0.000	0.000	0.000
HERC2	0.000	0.000	0.000	0.000	0.000	0.000	-1.029	0.000
PRKAR2B	0.000	0.000	0.000	0.000	0.000	-2.163	0.000	0.000
IL10	0.000	-4.451	0.000	0.000	3.793	0.000	0.000	0.000
TKT	0.000	0.000	0.000	0.000	0.000	-2.125	0.607	0.000
GALNT10	0.000	0.000	0.000	0.000	0.000	-2.101	0.000	0.000
CALM2	-1.172	0.000	0.000	0.000	0.000	0.000	0.000	0.000
DVL1	0.000	0.000	0.000	0.000	0.000	-4.647	0.000	0.000
DYNC1LI2	-0.632	0.000	0.000	0.000	0.000	0.000	0.000	0.000
KLF2	0.000	0.000	-1.142	-0.725	0.000	0.000	0.000	0.000
CD274	2.229	0.000	0.000	0.000	0.000	0.000	0.000	0.000
PRCP	-0.869	0.000	0.000	0.000	0.000	0.000	0.000	0.000
HTR4	0.000	0.000	0.000	2.604	0.000	0.000	0.000	0.000
RNF31	0.000	0.000	0.000	0.629	0.000	0.000	0.000	0.000
BTG1	0.000	-2.443	0.000	0.989	0.000	0.000	0.000	0.000
PIK3R3	1.885	0.000	0.000	0.000	0.000	2.219	0.000	-2.104
CXCL1	0.000	0.000	-0.899	1.785	0.000	0.000	1.089	1.276
ACSM3	0.000	0.000	0.000	3.175	0.000	0.000	0.000	2.432
MBD4	0.000	0.000	0.000	0.000	0.000	-0.839	0.000	0.000
SSTR3	0.000	3.473	0.000	3.763	0.000	0.000	0.000	0.000
TBXA2R	0.000	0.000	0.000	0.000	-2.012	6.746	0.000	0.000
SLC39A6	0.000	0.000	0.000	0.903	0.000	0.000	0.000	0.000
SLC7A11	0.000	0.908	1.431	0.000	0.000	1.591	1.263	0.000

VAMP3	-1.372	0.000	0.000	0.000	0.000	-3.510	0.000	0.000
TBX3	0.000	0.000	0.000	0.601	0.860	0.000	0.000	0.746
GNAI1	1.603	0.000	0.646	0.957	0.688	1.309	0.000	1.148
RGS12	0.000	0.000	0.000	0.000	0.000	0.000	0.000	-5.316
ATP1B1	0.000	0.000	0.000	0.593	0.000	0.000	0.000	0.000
WNT2	0.000	0.000	0.000	0.000	0.000	5.926	0.000	0.000
PGM2	0.000	0.000	0.000	0.000	0.000	-2.793	0.000	0.000
STK24	1.769	0.930	0.000	1.290	0.000	1.412	0.000	0.000
CAT	0.000	0.000	0.000	-1.580	0.000	0.000	0.943	0.000
GNB1	0.000	0.000	0.000	-1.034	0.000	0.000	0.000	0.000
PIK3R1	4.112	0.000	0.000	1.490	1.336	0.000	0.000	2.281
MAP3K2	0.000	0.000	-1.502	0.000	0.000	0.000	0.000	0.000
ASB2	0.000	4.181	0.000	3.625	0.000	0.000	0.000	0.000
SCP2	0.000	0.000	0.000	0.000	0.000	0.000	0.000	-1.621
P4HA1	0.000	0.000	0.000	0.000	-1.390	0.000	0.000	0.000
GPNMB	4.033	1.625	0.000	2.087	2.470	4.067	0.000	3.857
CTLA4	0.000	0.000	0.000	0.000	2.559	0.000	0.000	2.829
CAMP	0.000	0.000	0.000	0.000	0.000	5.541	0.000	0.000
AP2M1	0.000	0.000	0.000	0.000	0.000	0.763	0.000	0.876
SLC25A13	0.000	0.667	0.000	0.000	0.000	0.000	0.000	0.000
GAS1	5.387	0.000	0.000	0.000	0.000	0.000	0.000	0.000
GNGT2	2.348	1.240	0.000	3.197	1.439	2.571	0.000	1.476
SEPTIN7	0.000	0.000	0.000	0.000	0.000	-4.784	0.000	0.000
ADAR	-4.976	0.000	0.000	0.000	0.000	0.000	0.000	0.000
TNNI3	0.000	-3.664	0.000	0.000	0.000	0.000	0.000	0.000
NOLC1	2.204	0.674	0.815	1.983	0.587	2.648	0.000	2.021
TUBA4A	0.000	0.000	0.000	0.000	0.000	0.000	0.000	4.122
PRKCD	1.761	0.000	0.000	0.000	0.000	0.000	0.000	0.000
MYL12A	0.000	0.000	-0.735	0.000	0.000	0.000	0.000	0.000
KIF7	2.761	1.402	1.370	2.450	1.238	2.715	0.000	2.398
PDE2A	2.482	1.765	1.740	3.302	0.000	3.875	-2.143	4.231
WNT7A	0.000	0.000	0.000	3.034	0.000	0.000	0.000	2.252
ALDH1A2	3.138	2.070	1.488	3.023	2.432	3.459	-1.980	4.200
ATP6V1D	0.000	0.000	0.000	0.000	0.000	-1.192	0.000	0.000
ATP2A3	0.000	3.685	0.000	2.991	4.037	0.000	0.000	0.000
NDUFV2	-1.297	0.000	0.000	0.000	0.000	0.000	0.000	0.000
IL3	0.000	0.000	0.000	2.922	0.000	4.693	0.000	2.506
BMP6	0.000	0.000	0.000	3.517	0.000	0.000	-3.370	2.718
CLCNKB	0.000	0.000	0.000	2.809	0.000	0.000	0.000	0.000
FZD1	2.166	0.000	0.000	0.000	0.000	0.000	0.000	0.000
GLRA1	0.000	0.000	0.000	3.814	0.000	0.000	0.000	0.000
LIG4	0.000	0.000	0.000	0.000	0.000	2.788	0.000	0.000
IL32	2.046	0.000	0.000	0.000	0.000	0.000	0.000	0.000

PLA2G16	0.000	0.000	0.000	2.623	2.378	3.673	0.000	0.000
ATP6V1B1	2.465	1.799	1.120	2.997	1.760	3.866	0.000	3.698
TWIST1	0.000	0.000	0.000	0.000	0.000	0.000	-0.726	0.000
STAT5B	-1.530	0.000	0.000	0.000	0.000	0.000	0.000	0.000
TADA3	4.593	2.263	0.000	3.202	2.176	0.000	0.000	0.000
COX6A2	0.000	0.000	0.000	0.000	0.000	5.950	0.000	0.000
INPPL1	0.000	0.000	0.000	0.000	0.767	2.472	0.000	0.000
SCN5A	0.000	0.000	0.000	3.631	0.000	0.000	0.000	0.000
MC4R	4.576	0.000	0.000	2.278	0.000	0.000	0.000	0.000
NPPC	0.000	2.184	0.000	2.741	0.000	0.000	-3.302	4.486
THBS1	0.992	0.000	0.000	0.000	0.000	0.000	0.000	0.000
GANAB	1.046	0.000	0.000	0.918	0.000	2.037	0.000	1.310
AOC3	4.268	0.000	0.000	2.368	0.000	3.710	0.000	3.551
RASSF2	-2.204	0.000	0.000	0.000	0.000	0.000	0.000	0.000
SETD7	2.835	0.000	0.000	0.000	0.000	0.000	0.000	0.000
MAF	0.000	0.000	0.000	0.000	0.000	6.756	0.000	0.000
KCNMA1	0.000	0.000	-0.614	0.000	0.000	-1.637	0.000	0.000
ABCB4	0.000	0.000	0.000	0.000	0.000	0.000	0.000	4.400
TRAF5	0.000	0.000	0.000	0.000	0.000	0.000	-3.419	0.000
NR3C1	3.779	2.827	0.000	0.000	0.000	0.000	0.000	0.000
NR1H4	0.000	2.225	1.615	3.146	2.291	0.000	0.000	2.596
TINF2	2.655	0.000	0.000	0.000	0.000	0.000	0.000	0.000
TNFSF18	4.056	1.938	2.838	3.582	0.000	4.767	-4.357	3.498
CACNG3	0.000	0.000	0.000	2.843	0.000	0.000	0.000	3.804
ATP6V1C1	1.442	0.000	0.000	0.000	0.000	-4.670	0.000	0.000
CXCR3	6.159	0.000	0.000	0.000	0.000	0.000	0.000	0.000
HES1	0.000	0.000	0.000	0.887	0.000	2.757	0.000	0.000
PLCB4	0.000	0.000	0.000	-0.669	0.000	0.000	0.000	0.000
P2RY13	0.000	0.000	0.000	2.926	0.000	0.000	-3.514	0.000
NOX1	4.120	3.867	0.000	3.377	0.000	0.000	0.000	0.000
LCAT	0.000	3.129	0.000	0.000	0.000	0.000	0.000	0.000
IL1R2	0.000	0.000	0.000	3.056	0.000	0.000	0.000	0.000
MFN2	-4.768	-0.732	0.000	0.000	0.000	0.000	0.000	0.000
CTSE	0.000	3.365	0.000	0.000	0.000	0.000	0.000	0.000
FOXD3	4.862	2.531	0.000	3.013	0.000	0.000	-3.612	0.000
COL4A5	2.254	0.000	0.000	0.000	0.000	0.000	0.000	0.000
PTGDR2	0.000	0.000	0.000	2.987	3.032	3.592	0.000	4.511
FLOT1	0.000	1.167	0.000	0.000	0.000	0.000	0.000	0.000
CCT5	-0.966	0.000	0.000	0.000	0.000	0.000	0.000	0.000
PRPF8	0.000	0.000	0.000	0.000	-0.634	0.000	0.000	0.000
HOMER2	3.290	0.000	0.000	0.000	0.000	0.000	0.000	2.665
HOXB1	3.416	2.307	0.000	2.339	1.890	2.515	0.000	2.207
DMD	0.000	1.367	0.000	0.000	0.000	0.000	0.000	0.000

RIMS2	2.708	1.900	0.000	2.864	1.881	3.625	0.000	3.498
CREB1	-1.055	0.000	0.000	0.000	0.000	0.000	0.000	0.000
DCTN6	-1.005	0.000	0.000	0.000	0.000	0.000	0.000	0.000
KDR	4.538	0.000	0.000	1.848	0.000	0.000	0.000	0.000
SAE1	0.000	0.000	0.000	0.000	0.000	-0.966	0.000	0.000
OTX2	0.000	0.000	0.000	3.973	0.000	0.000	0.000	0.000
TEK	0.000	0.000	0.000	1.720	0.000	0.000	0.000	1.013
RBCK1	0.000	-3.786	0.000	0.000	0.000	0.000	0.000	0.000
LEF1	0.000	0.000	0.837	1.463	0.000	1.806	0.000	1.501
RAD52	0.000	0.000	0.000	0.000	0.000	0.000	-2.973	0.000
ELK1	0.000	2.512	0.000	0.000	0.000	4.128	0.000	0.000
MAP3K20	-5.004	0.000	0.000	0.000	0.000	-4.715	0.000	0.000
TUBB3	0.000	0.000	0.000	0.000	0.000	-4.948	0.000	0.000
ST6GAL1	0.000	0.000	0.000	4.082	0.000	0.000	0.000	0.000
PREP	2.300	0.000	0.000	0.000	0.000	-4.607	0.614	0.000
CDKN2A	0.000	0.000	0.000	0.000	0.000	-0.868	0.000	0.000
DAG1	0.000	0.000	0.000	0.000	0.000	-2.114	0.000	0.000
CISH	0.000	0.000	0.000	2.979	2.339	3.509	0.000	3.216
CD81	0.000	0.000	0.950	1.747	0.843	1.611	0.000	0.788
ANAPC5	0.661	0.000	0.000	0.000	0.000	0.000	0.000	0.000
ATP6V0E2	0.000	0.000	0.000	1.188	0.000	0.000	0.000	0.901
G6PC3	0.000	0.000	0.000	0.000	0.000	6.779	0.000	0.000
ARNT	2.753	0.000	0.000	1.981	0.000	0.000	0.000	0.000
DUSP10	0.000	1.669	0.000	0.000	0.000	0.000	0.000	0.000
MMP3	0.000	1.634	0.000	1.978	0.000	0.000	0.000	0.000
CARD18	5.581	0.000	0.000	3.114	0.000	0.000	-3.500	0.000
FOXO3	0.000	0.000	0.000	0.000	0.000	-4.338	0.000	0.000
CD1B	4.831	0.000	0.000	3.435	0.000	5.204	0.000	0.000
COL6A3	0.000	0.000	-1.679	0.000	0.000	0.000	0.000	0.000
RAPGEF3	0.000	0.000	0.000	3.059	0.000	4.828	0.000	2.878
NPPB	0.000	0.000	0.000	0.000	0.000	0.000	-4.808	0.000
CDH4	0.000	0.000	-1.141	0.000	0.000	0.000	-0.681	0.000
ADAM23	0.000	0.000	-3.620	0.000	0.000	0.000	0.000	0.000
MCM2	0.000	0.000	0.000	-0.609	0.000	0.000	0.000	0.000
CXCL8	0.000	0.000	0.000	2.745	0.000	3.852	0.000	2.795
EIF4G3	0.000	0.000	0.000	0.000	0.000	-1.017	0.000	0.000
IKBKG	0.000	1.323	0.000	1.487	1.473	2.255	0.000	2.123
GNPTAB	0.000	0.000	0.000	0.000	-1.521	0.000	0.000	0.000
IDE	2.391	0.000	0.000	0.000	0.000	0.000	0.000	0.000
BTG3	0.000	0.000	0.000	0.000	0.738	0.000	0.000	0.000
F2RL1	3.702	0.000	2.387	3.056	0.000	0.000	0.000	0.000
MAPRE1	0.000	0.000	0.000	0.000	0.000	-2.473	0.000	0.000
UBE2B	0.709	0.000	0.762	1.233	0.000	1.298	0.000	0.000

ADA	0.000	0.000	0.000	0.000	0.000	0.000	0.000	1.555
ACTN2	0.000	2.387	0.000	0.000	2.533	0.000	0.000	0.000
MMP7	0.000	0.000	0.000	0.000	3.315	0.000	0.000	0.000
RXFP1	0.000	0.000	0.000	2.918	0.000	5.563	0.000	0.000
MCM6	0.000	0.000	0.000	-1.662	0.000	3.345	0.000	0.000
DDX11	0.000	0.000	0.000	0.000	0.000	0.000	0.000	1.995
RHEB	-1.792	0.000	0.000	0.000	0.000	0.000	0.000	0.000
SOCS4	0.000	1.690	0.000	0.000	0.000	0.000	0.000	0.000
MKNK1	0.000	2.084	0.000	0.000	0.000	0.000	0.000	0.000
MGST1	0.000	0.000	0.000	3.759	0.000	0.000	0.000	0.000
GALNT6	0.000	0.000	0.000	2.349	0.000	3.567	-1.778	3.216
ANKRD1	0.000	0.000	-0.695	-1.118	0.000	-2.116	-1.165	0.000
UBE2NL	1.445	0.000	0.000	0.000	0.000	1.947	0.000	0.000
INHBA	0.000	0.000	0.000	1.061	0.000	2.040	1.143	0.000
CCNF	0.982	0.000	0.000	0.000	0.000	0.000	0.000	0.000
TGIF2	0.000	1.107	0.000	0.000	0.000	0.000	0.000	0.000
AHNAK	0.000	0.000	-0.667	0.000	0.000	0.000	-0.711	0.000
AP1B1	-1.171	0.000	0.000	0.000	0.000	0.000	0.000	0.000
MIF	0.000	0.000	0.000	0.000	0.000	1.453	0.000	0.000
CST3	1.963	0.000	0.000	1.592	0.588	2.355	0.000	1.874
CAB39	0.000	0.000	0.000	0.000	0.000	2.627	0.000	0.000
UBE2M	0.000	0.000	0.000	0.000	0.000	-0.795	0.000	0.000
CYC1	0.000	0.000	0.000	0.000	0.000	-2.352	0.000	0.000
YBX3	0.000	-1.735	0.000	1.360	-2.487	0.000	0.000	0.000
IRF7	0.000	0.000	0.000	0.000	0.000	0.000	-3.658	0.000
KAT2B	4.099	1.869	1.665	3.302	2.299	3.305	-1.977	3.452
RBL1	0.000	0.000	0.000	0.000	0.000	-1.186	0.000	0.000
BMP3	0.000	0.000	0.000	3.603	0.000	0.000	0.000	0.000
MLLT4	1.459	0.000	0.000	0.000	0.000	0.000	0.000	0.000
CPT1A	3.515	0.000	0.000	0.000	0.000	0.000	0.000	0.000
TELO2	0.000	0.000	-1.295	0.000	0.000	0.000	0.000	0.000
SEPTIN4	0.000	3.453	0.000	3.579	0.000	0.000	0.000	0.000
CEBPB	1.112	0.000	0.000	0.000	0.000	1.034	0.000	0.000
NDUFS4	0.000	0.000	0.000	1.245	0.000	1.136	0.000	0.749
TM7SF2	0.000	0.000	0.000	0.000	0.000	2.949	0.000	0.000
P2RY8	0.000	0.000	0.000	3.116	0.000	0.000	0.000	3.057
KIAA0947	0.000	0.000	0.762	0.000	0.000	0.000	0.000	0.000
IRF6	0.000	1.856	0.000	2.628	0.000	3.826	-2.758	2.792
VLDLR	0.000	1.234	0.000	2.307	0.000	2.674	0.000	3.468
LCP2	0.000	0.000	0.000	3.682	0.000	0.000	0.000	0.000
NRF1	1.728	0.000	0.000	0.000	0.000	0.000	0.000	0.000
PPP1R3D	0.000	0.000	0.000	0.000	0.000	7.770	0.000	0.000
ATP4A	0.000	0.000	0.000	3.706	0.000	0.000	0.000	0.000

COL6A2	0.000	0.000	-0.711	0.000	0.000	0.000	0.000	0.000
STAT2	0.000	0.000	0.820	0.000	0.000	0.000	0.000	0.000
DYNLL2	0.000	0.000	0.000	0.955	0.000	0.000	0.000	0.000
LAMC1	-4.331	-1.062	0.000	0.000	0.000	0.000	0.000	0.000
DAB2	0.000	0.000	0.000	0.000	0.000	-0.649	0.000	-0.626
PPP3CA	0.000	0.000	0.000	0.000	0.000	-3.609	0.000	0.000
GATA3	1.847	0.000	0.000	0.000	0.000	0.000	0.000	0.000
IQGAP1	0.000	0.000	0.000	0.000	0.000	-5.686	0.000	0.000
ERBB2IP	0.000	0.000	0.000	0.000	0.000	-1.730	0.000	0.000
CLEC7A	0.000	2.375	0.000	1.968	0.000	0.000	0.000	3.122
UGT1A1	0.000	0.000	0.000	2.394	0.000	0.000	0.000	1.816
NRIP1	-2.851	0.000	0.000	0.000	0.000	-2.582	0.000	0.000
ICOSLG	5.097	2.958	0.000	2.949	0.000	0.000	0.000	0.000
TFPI2	1.569	0.000	-0.862	0.868	0.000	1.441	0.000	0.934
CLN3	0.000	0.000	-1.622	-2.291	0.000	2.920	0.000	0.000
PTEN	0.000	0.982	0.672	1.509	0.000	2.459	-1.237	2.896
CD70	5.020	0.000	0.000	0.000	0.000	0.000	0.000	0.000
OSM	6.528	0.000	0.000	0.000	0.000	0.000	0.000	0.000
VDAC1	0.000	0.000	0.000	0.000	0.000	1.762	0.000	0.000
AQP4	0.000	0.000	1.587	2.634	0.000	3.935	-1.631	3.856
NFKB2	3.199	0.000	0.000	0.000	0.000	0.000	0.000	0.000
UQCRH	1.826	0.000	0.000	0.000	0.000	0.000	0.000	0.000
DERA	0.000	0.000	0.000	0.000	-1.056	0.000	0.000	0.000
HRH4	0.000	0.000	0.000	0.000	0.000	7.088	0.000	0.000
CAPN10	0.000	0.000	0.000	2.057	0.000	0.000	0.000	2.853
SRSF1	0.000	0.000	0.000	-0.984	0.000	0.000	0.000	0.000
ZHX3	-1.942	0.000	0.000	0.000	0.000	0.000	0.000	0.000
HCK	0.000	4.474	0.000	0.000	0.000	7.756	0.000	0.000
GADD45B	0.000	0.000	0.000	0.000	0.000	2.216	0.000	0.000
FNIP1	0.000	0.000	0.000	0.000	-3.763	0.000	0.000	0.000
RAD50	0.000	0.000	0.000	0.000	-1.122	-3.093	0.000	0.000
GCG	4.316	0.000	0.000	3.233	0.000	4.435	0.000	2.312
IDO1	2.807	1.856	0.000	2.870	0.000	3.037	-2.633	3.927
MSN	0.000	0.000	-0.644	0.000	0.000	0.000	0.000	0.000
PIK3CA	-4.483	0.000	0.000	0.000	0.000	-4.665	0.000	0.000
UQCRC2	2.253	0.000	0.000	1.261	0.000	0.000	0.000	0.000
DGAT1	0.000	0.000	0.000	2.966	0.000	0.000	0.000	3.253
EEF2K	0.000	2.315	0.000	2.447	2.293	0.000	0.000	2.307
TXNL4B	3.721	0.000	1.637	3.253	2.646	3.793	-1.400	2.711
FHIT	0.000	2.368	0.000	2.465	1.827	3.884	0.000	3.989
F13A1	0.000	0.000	0.000	0.000	0.000	6.135	0.000	0.000
CSNK1A1	0.000	0.000	0.000	0.000	0.000	-4.557	-0.666	0.000
